# Direct synthesis of amides from nonactivated carboxylic acids using urea as nitrogen source and Mg(NO_3_)_2_ or imidazole as catalysts†
†The paper is dedicated to the memory of Prof. J. M. J. Williams.
‡
‡Electronic supplementary information (ESI) available: Experimental procedures and spectroscopic characterisation of all organic compounds. See DOI: 10.1039/d0sc01317j


**DOI:** 10.1039/d0sc01317j

**Published:** 2020-05-19

**Authors:** A. Rosie Chhatwal, Helen V. Lomax, A. John Blacker, Jonathan M. J. Williams, Patricia Marcé

**Affiliations:** a Department of Chemistry , University of Bath , Claverton Down , Bath , BA2 7AY , UK . Email: pmv21@bath.ac.uk; b Centre for Sustainable Chemical Technologies , University of Bath , Claverton Down , Bath , BA2 7AY , UK; c Institute of Process Research & Development , School of Chemistry , University of Leeds , Woodhouse Lane , Leeds LS2 9JT , UK

## Abstract

A new method for the direct synthesis of primary and secondary amides from carboxylic acids is described using Mg(NO_3_)_2_·6H_2_O or imidazole as a low-cost and readily available catalyst, and urea as a stable, and easy to manipulate nitrogen source. This methodology is particularly useful for the direct synthesis of primary and methyl amides avoiding the use of ammonia and methylamine gas which can be tedious to manipulate. Furthermore, the transformation does not require the employment of coupling or activating agents which are commonly required.

## Abstract 2

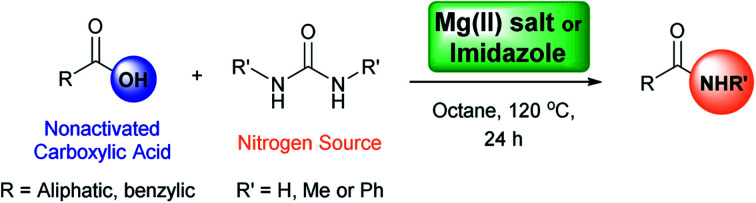
This methodology is particularly useful for the direct synthesis of primary and *N*-methyl amides using urea as a stable and easy to manipulate nitrogen source.

## Introduction

The importance of the amide functional group emerges from their presence in many crucial compounds such as proteins, fabrics, fertilizers, insecticides, plastics, drugs, and in a vast number of synthetic structures. For this reason, it is very relevant to develop new methods for the efficient synthesis of amides.

Traditional methods require the transformation of the acid into the corresponding acid chloride, to use the Schotten–Baumann reaction, or coupling reagents commonly used in peptide synthesis.^1^–^4^ Although these methods produce amides under mild reaction conditions and good yields, stoichiometric amounts of activating reagent are required, and an equivalent of waste is generated, making these low-atom economy processes. Besides, the removal of the corresponding by-product can be tedious increasing the cost of the transformation. New methodologies described for the synthesis of amides^5^,^6^ involve the use of catalysts, and employ starting materials such as esters,^7^–^17^ aldehydes,^18^–^27^ alcohols,^28^–^33^ nitriles,^34^–^45^ and oximes.^46^–^56^ The catalysts are mainly based on expensive metals such as rhodium, ruthenium, iridium, and palladium. Although the use of cheaper metals such as copper, iron, titanium, hafnium and zirconium have been recently reported.^5^,^6^,^44^,^56^–^60^ Transamination^61^–^64^ reactions to convert primary amides into more complex amides, and the acylation of amines to produce secondary amides are also important transformations reported in this field.^65^

The direct synthesis of secondary amides from nonactivated carboxylic acids is an important transformation that has been less exploited and studied.^57^,^64^,^66^ Secondary and tertiary amides can be obtained by condensation of the acid and the amine, but the competing acid–base reaction makes this coupling challenging, overcome by forcing conditions.^4^ Thermal amidations in the absence of a catalyst have been previously reported^67^–^72^ and are favoured by the use of apolar solvents such as toluene.^71^

**Scheme 1:**
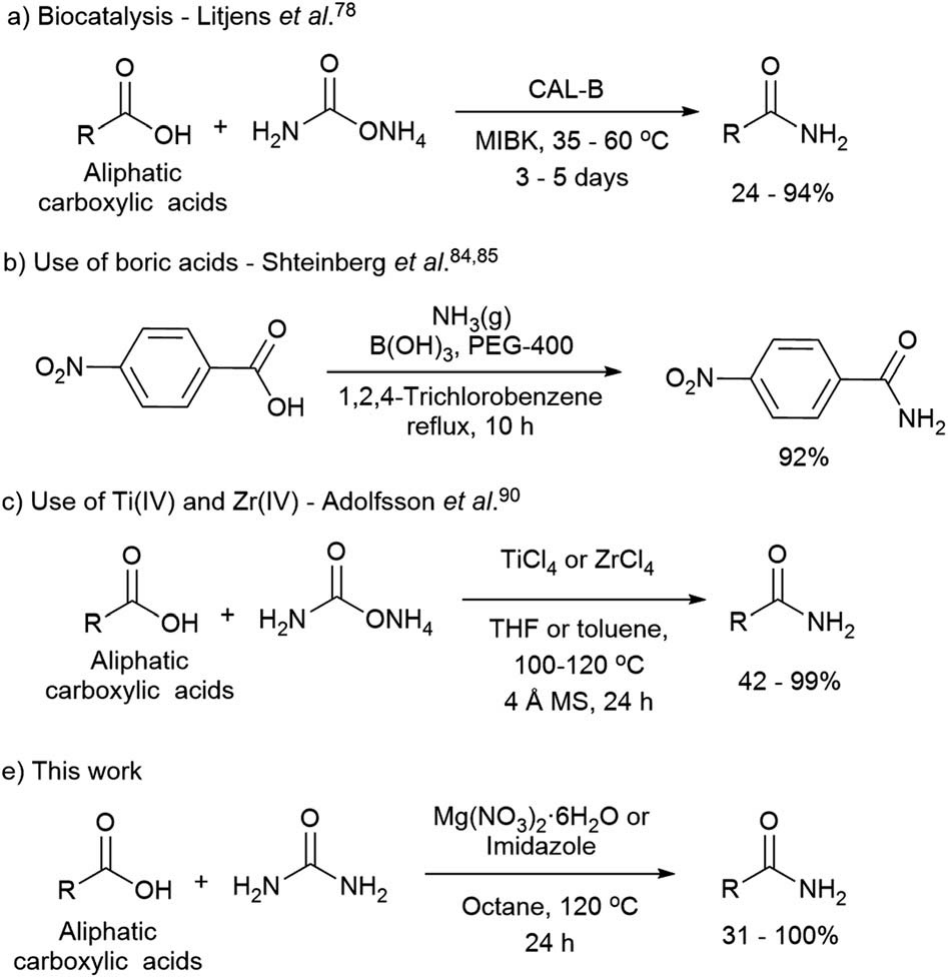
Relevant direct primary amide formation from carboxylic acids.

The direct synthesis of primary amides by this methodology is more challenging due to the low nucleophilic nature of the nitrogen source, and the use of coupling reagents is often required. The use of catalysts is an attractive approach for the direct formation of primary amides. The most relevant methodologies reported in this regard involve the use of enzymes such as lipases,^73^–^82^ boric acids,^83^–^89^ Group IV metals such as zirconium, titanium,^90^,^91^ and heterogeneous catalyst as ZrOCl_2_·8H_2_O and CAN combined with microwave radiation (Scheme 1). Although, the latter methodologies have been reported to be difficult to reproduce.^90^

The number of catalytic protocols reported for the synthesis of primary amides is still limited.^43^,^51^,^92^–^98^ In this work, we present a new protocol for the synthesis of amides from nonactivated carboxylic acids by direct coupling using a low-cost, readily available, and easy to manipulate catalyst and nitrogen source.

## Results and discussion

Our initial investigation identified urea as able to transform phenylacetic acid (**1**) into 2-phenylacetamide (**3**) (Table S1, see ESI‡). A catalyst screen was carried out to meet the abovementioned requirements (Table 1). Compared to the control, Group IV metals such as titanium and zirconium (Table 1, entries 2 and 3) improved the conversion,^90^ whilst others such as Ni(NO_3_)_2_, ZnCl_2_, iodide salts and protic acids showed little effect. Interestingly, Mg(OAc)_2_, imidazole and DMAP presented similar activities to those found with Zr(iv) and Ti(iv). Considering these results, and the low cost, availability and stability of magnesium salts and imidazole, the reaction conditions were further optimised using these two catalysts.

**Table 1:** Catalyst screen^*a*^

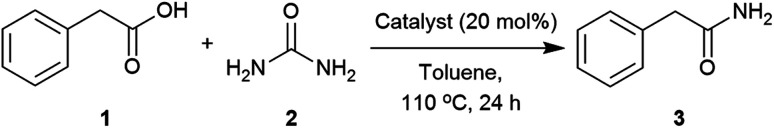		
Entry	Catalyst	Conversion^*b*^ (%)
1	—	12
2	**Cp**_**2**_ **ZrCl**_**2**_	**57**
3	**Ti(O** ^**i**^ **Pr)**_**4**_	**57**
4	Ni(NO_3_)_2_·6H_2_O	32
5	ZnCl_2_	10
6	LiBr	17
7	Sc(OTf)_3_	20
8	**Mg(OAc)**_**2**_ **·4H**_**2**_ **O**	**54**
9	AgI	8
10	KI	15
11	pTSA	8
12	Zn(OAc)_2_·2H_2_O	18
13	InCl_3_	7
14	NaI	11
15	Acetic acid	12
16	Nitric acid	9
17	CaI_2_	10
**18**	**Imidazole**	**58**
**19**	**DMAP**	**56**

^*a*^Reaction conditions: phenylacetic acid (1 mmol), urea (1 mmol), catalyst (20 mol%), PhMe (1 mL), 110 °C, 24 h.

^*b*^Conversions were determined by analysis of the crude ^1^H NMR spectra.

### Magnesium salts as catalyst

Applying the conditions used in the initial catalyst screen, the most suitable solvent was determined (Table S2, see ESI‡). Dipolar aprotic solvents, DMF and DMSO, showed low conversions,^71^,^99^ whilst polar solvents such as CPME (cyclopentylmethylether), isoamyl alcohol and butyronitrile revealed reasonable conversions. The use of non-polar solvents such as toluene and octane showed the highest conversions into the corresponding amide.^100^ These solvents enabled higher temperatures, and melting of the starting materials (**1** = 76 °C and **2** = 133 °C) was observed to give a second liquid phase. This polar dispersed phase might dissolve the magnesium salt catalyst, and the high concentrations in the droplets are expected to facilitate the reaction.^101^–^103^ Octane was selected to screen other magnesium salts (Table 2). All those tested showed a good catalytic activity with the best conversions achieved using Mg(OAc)_2_·4H_2_O, MgCl_2_ and Mg(NO_3_)_2_·6H_2_O. When magnesium acetate was used, detailed analysis of the crude reaction revealed the formation of acetamide as a by-product.^104^ Mg(NO_3_)_2_·6H_2_O was chosen as the most appropriate catalyst for further optimisation of the reaction conditions. The use of 2 equivalents of urea at 120 °C were found to give an optimal 93% conversion to the amide (Table 3, entries 3). Three equivalents of urea had a detrimental effect on the formation of 2-phenylacetamide (Table 3, entries 3 and 4). Varying the catalyst loading and reaction concentration, the best conditions identified were using 10 mol% of Mg(NO_3_)_2_·6H_2_O with a 1 M concentration in octane (Tables S6 and S7, see ESI‡).

**Table 2:** Screen of magnesium salts^*a*^

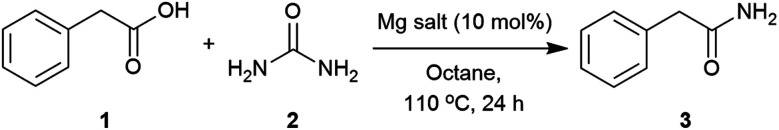		
Entry	Mg catalyst	Conversion^*b*^ (%)
1	—	26
2	**Mg(OAc)**_**2**_ **·4H**_**2**_ **O**	**68**
3	Mg turnings	51
4	**Mg(NO**_**3**_ **)**_**2**_ **·6H**_**2**_ **O**	**64**
5	MgO	54
6	Mg(OTf)_2_	61
7	**MgCl**_**2**_ **·6H**_**2**_ **O**	**65**
8	MgSO_4_	50

^*a*^Reaction conditions: phenylacetic acid (1 mmol), urea (1 mmol), Mg catalyst (10 mol%), octane (1 mL), 110 °C, 24 h.

^*b*^Conversions were determined by analysis of the crude ^1^H NMR spectra.

**Table 3:** Urea stoichiometry^*a*^

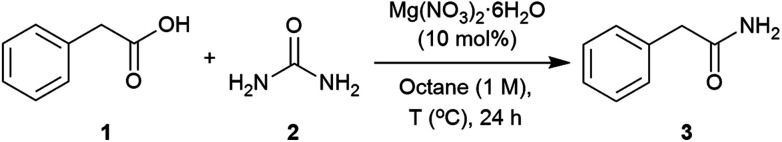			
Entry	Urea (equiv.)	Conversion^*b*^ (%) 110 °C	Conversion^*b*^ (%) 120 °C
1	0.5	52	51
2	1	64	69
**3**	**2**	**72**	**93**
4	3	55	85

^*a*^Reaction conditions: phenylacetic acid (1 mmol), Mg(NO_3_)_2_·6H_2_O (10 mol%), octane (1 mL), 24 h.

^*b*^Conversions were determined by analysis of the crude ^1^H NMR spectra.

The scope of the reaction was subjected to study. This methodology turned out to be effective for a wide range of aliphatic and phenylacetic acids (Table 4). Phenylacetic acids bearing electron-withdrawing and electron-donating groups were converted into the corresponding amides (**3**, **4**, **5**, and **6**) in 81–87% isolated yields. The sterically demanding substrate diphenylacetic acid was also successfully transformed into diphenylacetamide (**7**) in 92% yield, and aliphatic acids (**8**, **9**, **13, 14**, and **15**), with internal (**16**) and terminal (**17**) double bonds were also well-tolerated. Aliphatic acids containing conjugated double bonds (**18**) were more challenging substrates and lower conversions were observed even at elevated temperatures. The precursor benzoic acid (**10**) and substituted acids **11** and **12** showed very low reactivity under the reaction conditions, perhaps due to the delocalisation of electrons and subsequent decrease of electrophilicity of the carboxyl group. Surprisingly, heterocycles such as 2-picolinic acid and benzothiophene-2-carboxylic acid gave the amides **19** and **20** in excellent yields. Furthermore, the hydroxyl group in glycolic acid was also tolerated to give **21** in 68% yield.

**Table 4:** Substrate scope for the formation of primary amides from carboxylic acids using Mg(NO_3_)_2_·6H_2_O^*a*^ ^,^^*b*^

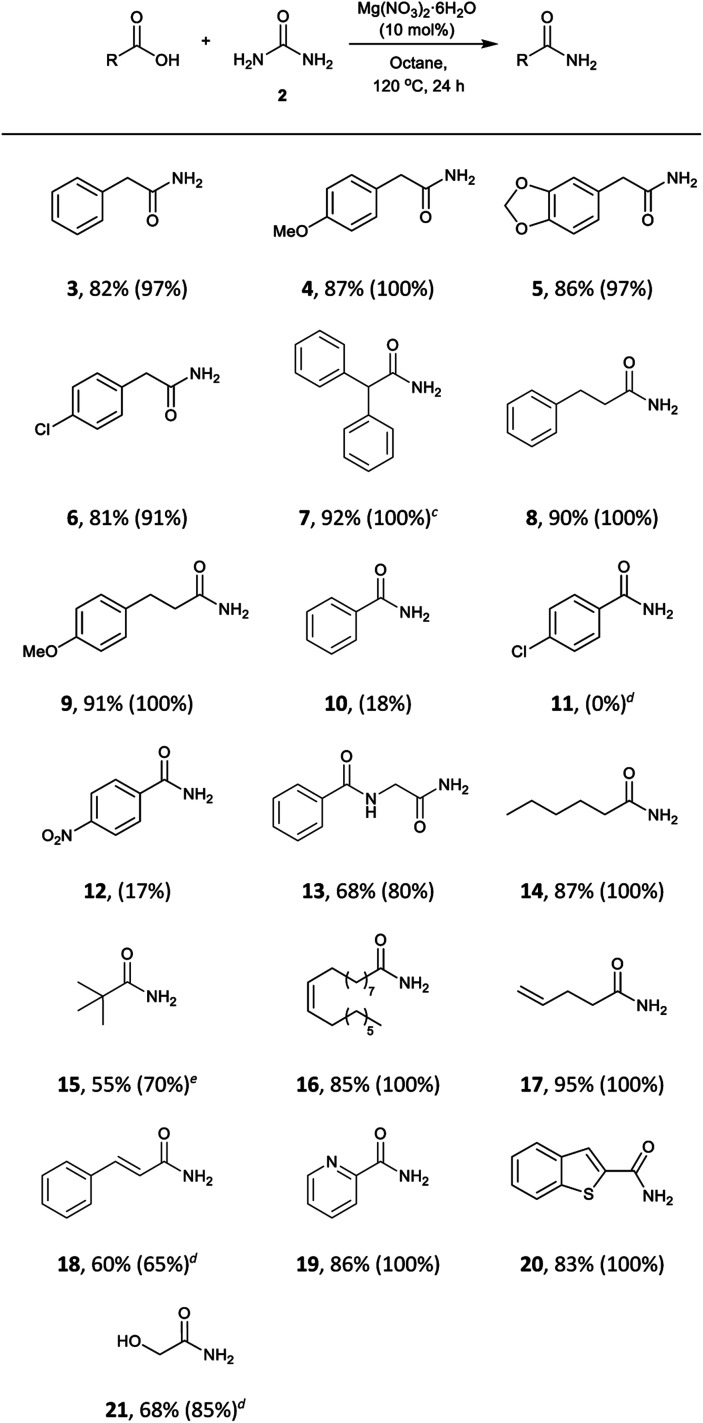

^*a*^Reaction conditions: carboxylic acid (3 mmol), urea (6 mmol), Mg(NO_3_)_2_·6H_2_O (10 mol%), octane (3 mL), 120 °C, 24 h.

^*b*^Isolated yields, conversions were determined by analysis of the crude ^1^H NMR spectra and are shown in parentheses.

^*c*^1 mmol scale.

^*d*^130 °C.

^*e*^Reaction at 130 °C did not improve the conversion. Longer reaction times did not show a substantial increase in the conversion.

We envisaged that our methodology could be also applied to the synthesis of secondary amides. *N*-Methyl amides are commonly obtained by direct coupling with methylamine gas and few alternatives methods are available.^105^–^108^ The use of *N*,*N*′-dimethylurea could be particularly useful, due to its availability and simpler handling. The scope of the reaction with this and *N*,*N*′-diphenylurea was tested with aliphatic and phenylacetic acids (Table 5). A wide range of aliphatic and phenylacetic acids was converted into the corresponding amides. For instance, *N*-methyl amides **22**, **23**, **24** and **25** were isolated in 89–96% yield. The method could be extended to aliphatic acids, and amides **26**, **27**, **29**, **30** and **31** were obtained in good yields. On the other hand, less electrophilic benzoic acid (**28**) was unreactive under these conditions. Three acids were also tested with *N*,*N*′-diphenylurea giving satisfactory yields of *N*-phenylacetamides **33**, **34** and **35**.

**Table 5:** Substrate scope for the formation of secondary amides from carboxylic acids using Mg(NO_3_)_2_·6H_2_O^*a*^ ^,^^*b*^

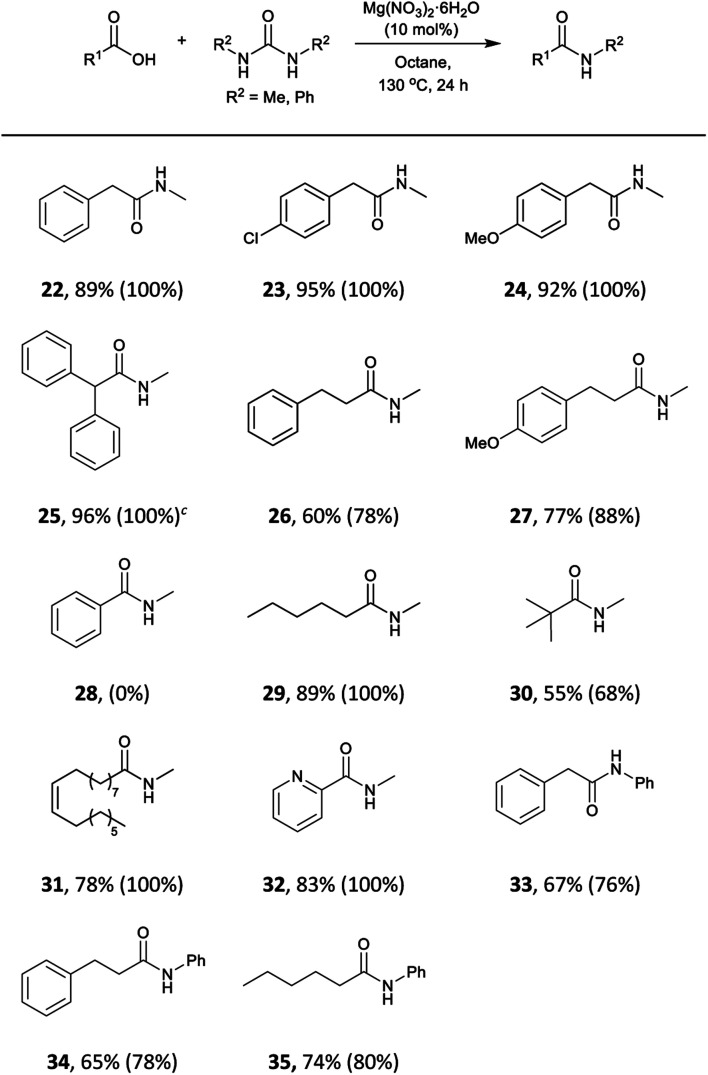

^*a*^Reaction conditions: carboxylic acid (3 mmol), urea (6 mmol), Mg(NO_3_)_2_·6H_2_O (10 mol%), octane (3 mL), 130 °C, 24 h.

^*b*^Isolated yields, conversions were determined by analysis of the crude ^1^H NMR spectra and are shown in parentheses.

^*c*^1 mmol scale.

### Imidazole as catalyst

The initial screen showed the potential of imidazole and DMAP as catalysts for this transformation (Table 1). So, our attention was turned into the inexpensive and readily available imidazole organocatalyst. Using the previously determined conditions, phenylacetic acid and urea were reacted to give phenylacetamide (**3**) in 78% conversion (Table 6, entry 4). Further improvement was achieved with 1.5 equivalents of urea, but higher loadings failed to obtain better conversions. Increasing the temperature to 120 °C led to the optimal reaction conditions (Table 6, entry 5).^109^,^110^

**Table 6:** Optimisation of the imidazole loading and amount of urea^*a*^

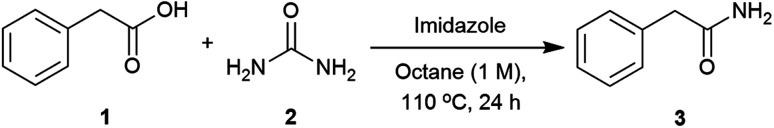				
Entry	Imidazole (equiv.)	Conversion^*b*^ (%)		
		Urea		
		1.0 equiv.	1.5 equiv.	2.0 equiv.
1	0		24	26
2	0.1		71	72
4	0.2	78	86	84
5	0.3		85	
6^*c*^	0.2		96	

^*a*^Reaction conditions: phenylacetic acid (1 mmol), urea and catalyst in octane (1 mL), 110 °C, 24 h.

^*b*^Conversions were determined by analysis of ^1^H NMR spectra.

^*c*^The temperature was increased to 120 °C.

Using the optimised conditions, the substrate scope of the carboxylic acid was investigated (Table 6). Phenylacetic acid and hydrocinnamic acid proceed to their corresponding amides **3** and **8** in 91% and 97%, respectively. The presence of electron-donating and -withdrawing groups at the *para* position had little effect on the conversion, and amides **4**, **5**, **6**, and **9** were obtained in high yields. Aliphatic groups were also well-tolerated and hexanamide (**13**) was produced in 89% yield. The presence of bulky substituents in the aromatic or aliphatic chain had a detrimental effect, and diphenylacetamide (**7**) was produced in 65% yield, 27% less than with Mg(NO_2_)_2_·6H_2_O. On the other hand, pivalamide (**15**) was obtained in 60% yield. Oleic acid gave **16** in 91% yield with no alteration of the double bond. Similar to the observations in Tables 4 and 5, the conjugated carboxylic acids did not perform well, with benzoic and cinnamic acids giving **10**, **11**, **12** and **18** in low conversions. 2-Picolinic acid and benzothiophene-2-carboxylic acid showed conversions into amides **19** and **20** of 65% and 100%, respectively, whilst glycolic acid was also converted into **21** in 61% yield, showing this catalyst also tolerates hydroxyl groups.

*N*,*N*′-Dimethylurea and *N*,*N*′-diphenylurea were also explored to synthesise secondary amides using imidazole (Table 7). In this case, 2 equivalents of urea and 130 °C were required to drive the reaction towards the formation of the amide (Table S13, see ESI‡). Both phenylacetic acid and hydrocinnamic acid gave **22** and **23** in 84% and 80% yields, respectively. In contrast, amidation of hexanoic acid into *N*-methylhexamide (**29**) gave only 70% yield, 19% less than the metal catalyst. Pivalic acid was converted into the amide **30** in only 38% yield, again indicating a steric problem. As with the other reactions, benzoic acid did not perform well in these conditions. Imidazole was an effective catalyst for making 2-picolinamide (**32**), obtained in 76% yield.

**Table 7:** Substrate scope for the formation of primary and secondary amides from carboxylic acids using imidazole^*a*^ ^,^^*b*^

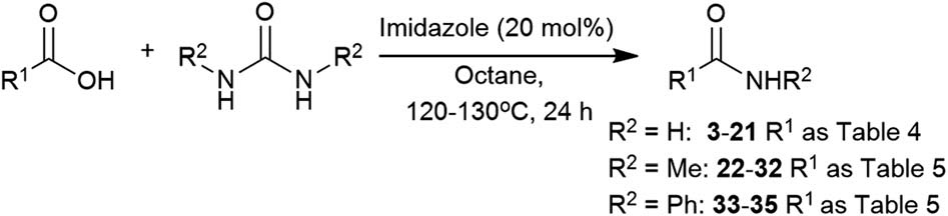			
Compound R^2^ = H^*a*^	Yield^*c*^ % (conv. %)	Compound R^2^ = Me or Ph^*b*^	Yield^*c*^ % (conv. %)
**3**	91 (100)	**22**	84 (100)
**4**	91 (100)	**23**	93 (100)
**5**	90 (97)	**24**	95 (100)
**6**	96 (100)	**25**	89 (100)
**7**	65 (73)	**26**	80 (100)
**8**	97 (100)	**27**	93 (100)
**9**	90 (97)	**28**	(25)
**10**	(30)	**29**	70 (88)
**11**	(18)	**30**	38 (50)
**12**	(33)	**31**	60 (84)
**13**	74 (86)	**32**	76 (84)
**14**	89 (100)	**33**	55 (72)
**15**	60 (72)	**34**	68 (85)
**16**	91 (100)	**35**	63 (78)
**18**	52 (63)		
**19**	42 (65)		
**20**	79 (100)		
**21**	61 (78)		

^*a*^Reaction conditions: carboxylic acid (3 mmol), urea (4.5 mmol), imidazole (20 mol%), octane (3 mL), 120 °C, 24 h.

^*b*^Reaction conditions: carboxylic acid (3 mmol), urea (6 mmol), imidazole (20 mol%), octane (3 mL), 130 °C, 24 h.

^*c*^Isolated yields, conversions were determined by analysis of the crude ^1^H NMR spectra and are shown in parentheses.

When *N*,*N*′-diphenylurea was used to synthesise *N*-phenylamides with imidazole catalyst, lower conversions were obtained. Phenylacetic acid, hydrocinnamic acid and hexanoic acid gave the corresponding amides **33**, **34** and **35** in 55%, 68% and 63% yields respectively.

### Mechanistic insights

**Scheme 2:**
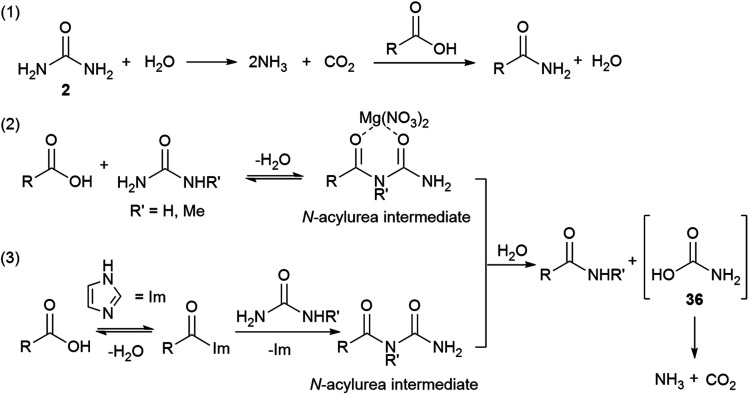
Plausible mechanisms for the formation of amides.

A slow uncatalysed reaction between phenylacetic acid and urea was observed (Tables 1 and 2, entries 1), however, the addition of a suitable Lewis acid or an organocatalyst considerably improves the rates and conversions. Since the reaction mechanism was unclear, three models were proposed (Scheme 2): (1) decomposition of urea and direct amidation; (2) magnesium salt facilitates the formation of an *N*-acylurea intermediate which can be hydrolysed to produce the amide, and an unstable carbamic acid, followed by decarboxylation of the later. To explain the observed products, reaction with the more substituted urea nitrogen is required; (3) condensation of the carboxylic acid with imidazole to form *N*-acyl imidazolium, this activated amide would then react with urea to produce an *N*-acylurea intermediate, again, breaking down to form ammonia and carbon dioxide.

**Scheme 3:**
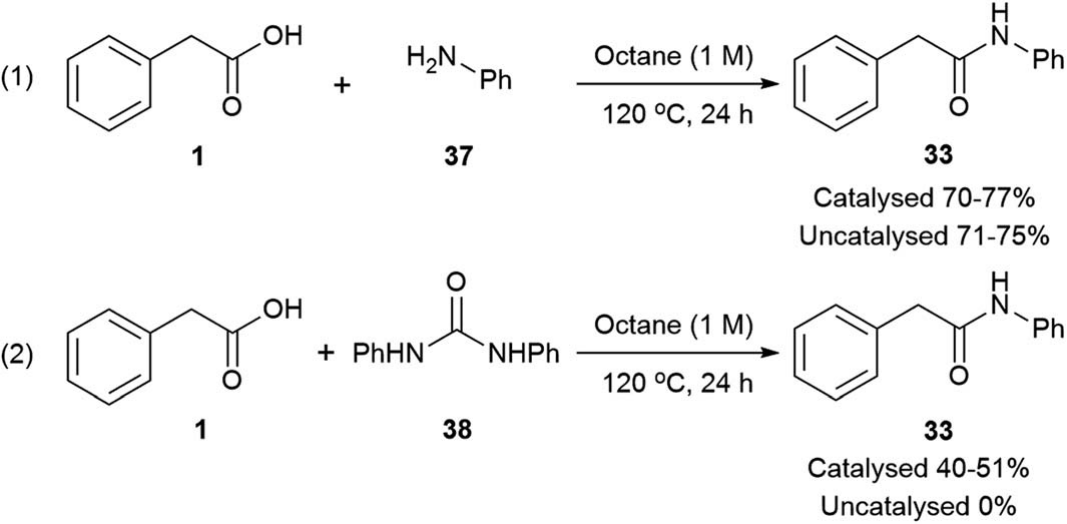
Conversions on the direct amidation of phenylacetic acid *vs.* amidation using *N*,*N*′-diphenylurea.

According to mechanism (1), the decomposition of urea into ammonia and CO_2_ is reported to take place at temperatures above 152 °C;^111^ even though the temperatures in our reactions are not as high as these, the possibility of a catalysed urea degradation was investigated. Urea was treated with either imidazole or Mg(NO_3_)_2_·6H_2_O under the reaction conditions for 24 hours. The analysis of the reaction crudes by ^1^H NMR showed the presence of urea indicating no degradation. Furthermore, gravimetric analysis, before and after reaction, gave 96% urea recovery, suggesting no degradation had taken place. The reaction was repeated with *N*-methylurea, *N*,*N*′-dimethylurea and *N*,*N*′-diphenylurea, and in each case >92% of the starting material was recovered. To investigate this further, phenylacetic acid (**1**) was exposed to the reaction conditions using aniline as the nitrogen source, which might be formed during the degradation of *N*,*N*′-diphenylurea (Scheme 3, eqn (1)). Similar conversions were observed when the reaction was carried out with and without imidazole or magnesium catalyst, suggesting it is not involved in the direct coupling.^112^ However, when phenylacetic acid (**1**) was reacted with *N*,*N*′-diphenylurea, the uncatalysed reaction gave no product, but in the catalysed reactions conversions of 40–51% were observed (Scheme 3, eqn (2)); notably less than direct amidation (70–77% conversion) but expected, as anilines are well known to undergo direct amidation.^72^

A previous report describes the CO_2_ evolved in the acidolysis of ureas using ^13^C-labelled carboxylic acids derives from the urea, and support other research that invokes a carbamic acid intermediate.^113^,^114^

**Scheme 4:**
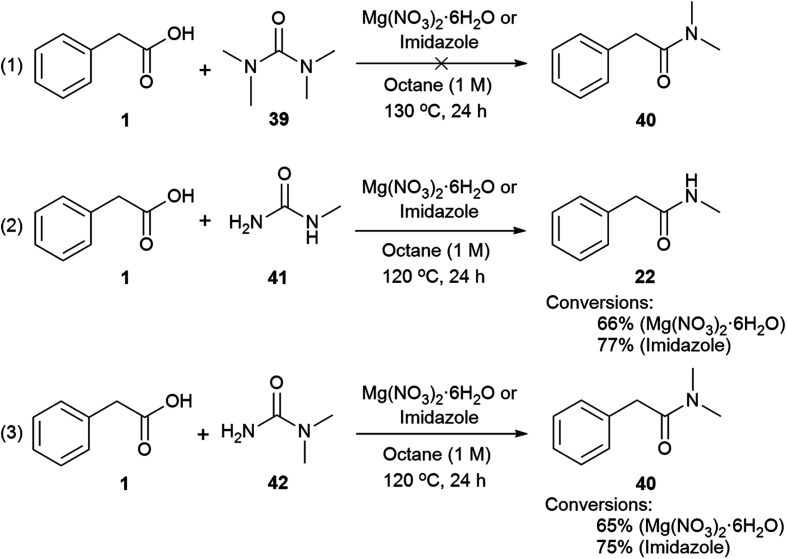
Use of tetrasubstituted and asymmetric ureas.

Mechanisms (2) and (3) were tested with unsymmetrical substituted ureas. The highly hindered *N*,*N*,*N*′*N*′-tetramethylurea did not form the tertiary amide **40** even at 130 °C, indicating that the steric bulk interferes (Scheme 4, eqn (1)). However, the reaction between phenylacetic acid and *N*-methylurea or *N*,*N*-dimethylurea gave the secondary amides **22** an **40** in 66% conversion with MgNO_3_·6H_2_O and 77% with imidazole, along with traces of the primary amide (Scheme 4, eqn (2) and (3), Table S14, see ESI‡). In order to check the formation of ammonia during the reaction a litmus paper test was conducted. A colour change from yellow to blue was observed confirming the formation of a basic gas. These results might indicate that unsymmetrical ureas react to give the most substituted amide, and presumably carbamic acid which decomposes liberating carbon dioxide and ammonia.^113^,^114^ Water is required for this reaction and may come from the MgNO_3_·6H_2_O, or during condensation of acid with the imidazole. In either case a thermally unstable (*N*-alkyl)carbamic acid is implicated,^115^ (Scheme 2, eqn (2) and (3)). MgNO_3_·6H_2_O might coordinate to the 1,3-dicarbonyl, activating it to urea condensation.^116^ With imidazole, the direct reaction with urea was discounted, however its reaction with carboxylic acids and esters, in solvent under thermal conditions is reported.^117^–^119^ Protonated *N*-acyl imidazoles are known to react with amines and thiols, so it is reasonable that urea, may react to form the *N*-acylurea intermediate.^115^,^120^

The reaction mixture was analysed by HRMS after 6 hours and a species with *m*/*z* of 201.0636 was found, which corresponds with the sodium adduct of the *N*-acylurea intermediate (theoretical *m*/*z* 201.0640 [M + Na]^+^). To test the reactivity of the *N*-acylurea intermediate, *N*-pivaloylurea was synthesised and subjected to different reaction conditions,^121^ and conversion to pivalamide was analysed by ^1^H NMR (Table 8).

**Table 8:** Decomposition of *N*-pivaloylurea into pivalamide^*a*^

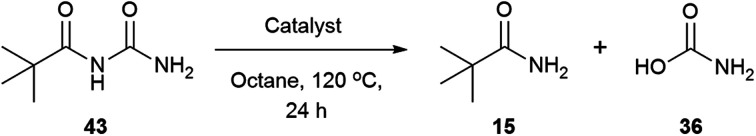				
Entry	Mg(NO_3_)_2_·6H_2_O (10 mol%)	Imidazole (20 mol%)	Water (2 equiv.)	Conversion^*b*^ (%)
1	✗	✗	✗	0
2	✗	✗	✓	6
3	✓	✗	✗	6
4	✗	✓	✗	4
5	✓	✗	✓	25
6	✗	✓	✓	14

^*a*^Reaction conditions: *N*-carbamoylpivalamide (1 mmol), water (2 mmol), imidazole (20 mol%) or Mg(NO_3_)_2_·6H_2_O (10 mol%), octane (1 mL), 120 °C, 24 h.

^*b*^Conversions were determined by analysis of ^1^H NMR spectra.

In the absence of the catalyst and water, only the starting material was recovered (Table 8, entry 1). The addition of two equivalents of water produced the pivalamide with 6% conversion (Table 8, entry 2). The presence of the catalyst did not improve the reaction outcome (Table 8, entries 3 and 4), whilst either catalyst alone failed to improve the conversions (Table 8, entries 5 and 6). However, when the catalyst and water were combined, 25% and 14% of amide were detected (Table 8, entries 5 and 6). The conversions are less than pivalic acid and urea, (55% and 60% to **15**, Tables 4 and 7). To dismiss a substrate effect, *N*-phenylacetylurea was synthesised and subjected to the same study, and similar behaviour was observed.^121^,^122^ Since *N*-alkylcarbamic acids decompose readily in acidic media,^114^ 1 equivalent of hydrocinnamic acid was added, but also gave similar low conversions. These results suggested that other physico-chemical effects may be playing a role. Solubility studies indicated that phenylacetic acid is soluble in octane at 120 °C while urea and 2-phenylacetamide (**3**) are not. When all the starting materials were mixed together in octane at 120 °C a biphasic system was obtained, and at the end of the reaction a white solid corresponding to 2-phenylacetamide (**3**) was formed. In these conditions an on-solvent system may be occurring in which the urea, carboxylic acid and catalyst are at high concentration and form a polar, hydrogen bonded structure that facilitates the reaction.^101^,^102^ Besides, the liberation of ammonia and CO_2_ along with the precipitation of 2-phenylacetamide (**3**) might be the driving force of this transformation. The lack of solubility of the *N*-acylurea intermediate in octane could explain the slow reactivity observed in Table 8.

To investigate further the role of imidazole in this transformation, *N*-1-methylimidazole and 2-methylimidazole were used as catalysts (Table 9). A significant decrease in conversion, from 86% to 37%, was observed when 2-methylimidazole was employed (Table 9, entry 4). Whereas, *N*-1-methylimidazole gave 66% conversion, only slightly less than imidazole (86%) (Table 9, entry 3). These results suggest that the mode of activation is through the nitrogen lone pair, as the 2-methyl group blocks this position. This is supported by the similar catalytic activity of DMAP (Table 9, entry 5), that also has a sp^2^ nitrogen with a lone pair of electrons. The mechanism by which the reaction proceeds is still unclear and further studies are still undergoing to understand the reaction pathway.

**Table 9:** Use of *N*-1-methylimidazole, 2-methylimidazole and DMAP as catalysts^*a*^

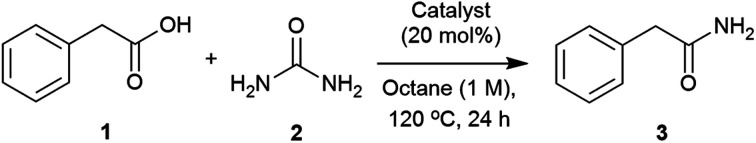		
Entry	Catalyst	Conversion^*b*^ (%)
1	—	24
2	Imidazole	86
3	*N*-1-Methylimidazole	66
4	2-Methylimidazole	37
5	DMAP	84

^*a*^Reaction conditions: phenylacetic acid (1 mmol), urea (1 mmol), catalyst (20 mol%), octane (1 mL), 120 °C, 24 h.

^*b*^Conversions were determined by analysis of ^1^H NMR spectra.

## Conclusions

A new method for the direct synthesis of primary amides from nonactivated carboxylic acids has been described, in which wasteful activating reagents are avoided. The methodology reports the use of cheap and readily available catalysts such as Mg(NO_3_)_2_·6H_2_O and imidazole, with urea as an atom-efficient nitrogen source. The process has been shown to produce not only primary, but secondary and a tertiary amides from readily available ureas. The method shows a broad scope of reaction, although conjugated carboxylic acids do not perform well. The reaction mechanism has been studied, and initial results point to the involvement of an *N*-acylurea intermediate, although, the pathway of its decomposition to the product remains unclear. Further studies are still undergoing to propose a more plausible mechanism.

## Conflicts of interest

There are no conflicts to declare.

## Supplementary Material

Supplementary information
