# Supplementary material for: Direct synthesis of amides from nonactivated carboxylic acids using urea as nitrogen source and Mg(NO3)2 or imidazole as catalysts
Source: Chem Sci. 2020 May 19;11(22):5808–18. doi: 10.1039/d0sc01317j (PMC7416778; doi:10.1039/d0sc01317j)
Supplement: Supplementary file 1 [file SC-011-D0SC01317J-s001.pdf]

Electronic Supplementary Material (ESI) for Chemical Science.
This journal is © The Royal Society of Chemistry 2020

SUPPORTING INFORMATION

Direct Synthesis of Amides from Nonactivated Carboxylic Acids using Urea as Nitrogen Source and $Mg(NO_3)_2$ or Imidazole as Catalysts

A. Rosie Chhatwal,[a] Helen V. Lomax,[b] A. John Blacker,[c*] Jonathan M. J. Williams,[a] Patricia Marcé[a*]

[a]*Department of Chemistry, University of Bath, Claverton Down, Bath, BA2 7AY, UK*

[b]*Centre for Sustainable Chemistry Technologies, University of Bath, Claverton Down, Bath, BA2 7AY, UK*

[c]*Institute of Process Research & Development, School of Chemistry, University of Leeds, Woodhouse Lane, Leeds, LS2 9JT, UK*

Table of Contents

1. General Remarks ... 3
2. Nitrogen Source Screen ... 3
3. Catalyst Screen ... 4
4. Optimisation of the Reaction Conditions using Magnesium Salts as Catalyst ... 4
   4.1. Solvent screen ... 4
   4.2. Screen of the magnesium salt ... 5
   4.3. Study of the optimal temperature and optimal concentration of urea ... 5
   4.4. Optimisation of the catalyst loading ... 6
   4.5. Optimisation of the concentration ... 6
   4.6. General procedure for the synthesis of primary amides using $Mg(NO_3)_2{\cdot}6H_2O$ ... 7
   4.7. General procedure for the synthesis of *N*-methylamides from using *N*,*N*'- dimethyl urea ... 7
5. Optimisation of the Reaction Conditions using Imidazole as Catalyst ... 7
   5.1. Imidazole and DMAP screen ... 7
   5.2. Nitrogen source screen ... 8
   5.3. Solvent screen ... 8
   5.4. Temperature screen ... 9
   5.5. Optimisation of the concentration ... 9
   5.6. General procedure for the synthesis of primary amides using imidazole ... 10
   5.7. Optimisation of the reaction conditions for the synthesis of secondary amides using imidazole ... 10
   5.8. General procedure for the synthesis of secondary amides using imidazole ... 11
6. Use of Other Ureas for the Synthesis of Amides ... 11
   6.1. Use of *N*,*N*,*N*',*N*'-tetramethylurea ... 11
   6.2. Use of *N*-methylurea ... 11
   6.3. Use of *N*,*N*-dimethylurea ... 12
7. Mechanistic Insights ... 13
   7.1. Decomposition of urea ... 13
   7.2. Direct amidation using aniline[1] ... 13
   7.3. Synthesis of *N*-carbamoylpivalamide[2] ... 13
   7.4. Synthesis of *N*-carbamoylphenylacetamide[3] ... 14
   7.5. Formation of amides from the *N*-acylurea intermediates ... 14
8. Synthesis of Primary Amides ... 15
9. Synthesis of Secondary Amides ... 24
10. ^{1}H and ^{13}C NMR. ... 31

11. References..60

1. General Remarks

All chemicals and solvents used were reagent grade and used as supplied unless otherwise specified. Chemicals were purchased from Acros Organics, Alfa Aesar or Sigma-Aldrich. Analytical thin layer chromatography (TLC) was performed on Merck® silica gel 60 F254 plastic plates. Organic compounds were visualized by UV (254 nm) irradiation or dipping the plate in a phosphomolybdic acid (PMA) solution. Flash column chromatography was carried out using forced flow or by gravity of the indicated solvent on Aldrich silica gel 60 (230 - 400 mesh). ^1H and ^{13}C NMR spectra were recorded on Agilent 500, Bruker AV 400 or Bruker AV 300 spectrometer in $CDCl_3$ or d^6-dmso as solvents. Chemical shifts (δ) were referenced internally to residual protic solvent signal for $CDCl_3$ (7.26 ppm) and d^6-dmso (2.5 ppm). Multiplicities are presented as singlet (s), broad singlet (br s), doublet (d), triplet (t), triplet of triplets (tt), quadruplet (q), quintet (quint), and multiplet (m). Coupling constants (*J*) were expressed in Hertz (Hz). HRMS-ESI were run on an Agilent® 1200 Series LC/MSD coupled to a micrOTOF electrospray time-of-flight (ESI-TOF) mass spectrometer (Bruker Daltonik). Infra-red spectra were recorded on a Perkin Elmer Spectrum 100 FT-IR spectrometer, using an universal ATR accessory for sampling with relevant absorbance quoted as ν in cm^{-1}. Uncorrected melting points were determined using Stuart SMP10 melting point equipment using closed end glass capillary.

2. Nitrogen Source Screen

A carousel tube was charged with phenylacetic acid (136 mg, 1.0 mmol) and the nitrogen source (2.0 mmol). Toluene (1 mL) was then added and the reaction was stirred at 110 °C for 24 hours. The reaction was allowed to cool to room temperature and redissolved in methanol. The solvent was removed *in vacuo* on a rotary evaporator. Conversions were determined by analysis of the crude ^1H NMR by comparison of the peaks at 3.57 (s, 2H, CH_2, **1**) and 3.36 (s, 2H, CH_2, **3**). The samples were prepared using d^6-dmso as a solvent.

Table S1.

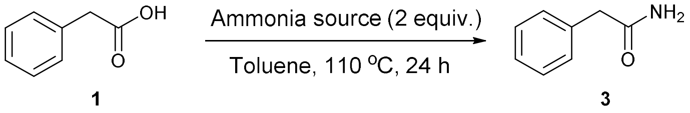

Entry	Ammonia source	Conversion (%)
1	Ammonium carbamate	12
2	Ammonium formate	12
3	Ammonium acetate	0
4	Ammonium iodide	0
5	Ammonium chloride	0
6	Formamide	3
7	Urea	17
8	Malonamide	0
9	NH_3 in Dioxane	0

3. Catalyst Screen

A carousel tube was charged with phenylacetic acid (1.0 mmol), urea (1.0 mmol) and the appropriate catalyst (20 mol%). Toluene (1 mL) was used as the solvent, and the reaction was stirred at 110 °C for 24 hours. After being allowed to cool to room temperature, the crude reaction was redissolved in methanol and the solvent was removed *in vacuo*. Conversions were determined by analysis of the crude ^{1}H NMR spectra using d^{6}-dmso as a solvent.

Table S2.

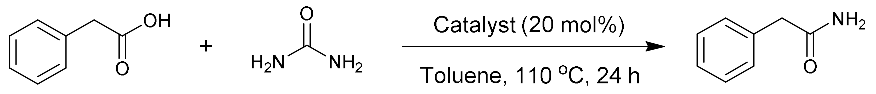

Entry	Catalyst	Conversion (%)
1	-	12
2	Cp_2ZrCl_2	57
3	$Ti(O^iPr)_4$	57
4	$Ni(NO_3)_2{\cdot}6H_2O$	32
5	$ZnCl_2$	10
6	LiBr	17
7	$Sc(OTf)_3$	20
8	$Mg(OAc)_2{\cdot}4H_2O$	54
9	AgI	8
10	KI	15
11	*p*TSA	8
12	$Zn(OAc)_2{\cdot}2H_2O$	18
13	$InCl_3$	7
14	NaI	11
15	Acetic acid	12
16	Nitric acid	9
17	CaI_2	10
18	Imidazole	58
19	DMAP	56

4. Optimisation of the Reaction Conditions using Magnesium Salts as Catalyst

4.1. Solvent screen

Following the general procedure described in section 3, $Mg(OAc)_2{\cdot}4H_2O$ (10 mol%) was used as the catalyst species and the corresponding solvent (1 mL) was added. After removal of the solvent *in vacuo*, the resulting crude reaction mixtures were analysed by their ^{1}H NMR spectra.

Table S3.

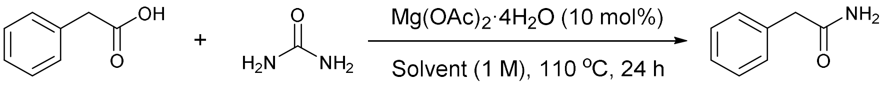

Entry	Solvent	Conversion (%)
1	Toluene	52
2	*p*-Xylene	42
**3**	**Octane**	**68**
4	Cyclopentyl methyl ether	43
5	Butyronitrile	45
6	Isoamyl alcohol	43
7	DMF	4
8	DMSO	3

4.2. Screen of the magnesium salt

Following the general procedure described in section 3, the corresponding magnesium salts (10 mol%) were added in the reaction using octane (1 mL) as solvent. After removal of the solvent *in vacuo*, the resulting crude reaction mixtures were analysed by their ^{1}H NMR spectra.

Table S4.

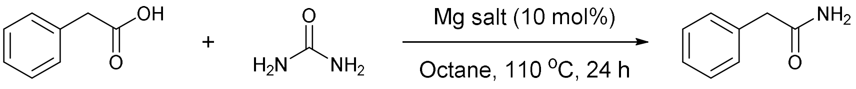

Entry	Mg catalyst	Conversion (%)
1	-	26
**2**	**$Mg(OAc)_2·4H_2O$**	**68**
3	Mg turnings	51
**4**	**$Mg(NO_3)_2·6H_2O$**	**64**
5	MgO	54
6	$Mg(OTf)_2$	61
**7**	**$MgCl_2·6H_2O$**	**65**
8	$MgSO_4$	50

4.3. Study of the optimal temperature and optimal concentration of urea

Following the general procedure described in section 3, a carousel tube was charged with $Mg(NO_3)_2·6H_2O$ (10 mol%) and the corresponding amount of urea. Octane (1 mL) was added and the reaction was stirred at 110 °C or 120 °C for 24 hours. After removal of the solvent *in vacuo*, the resulting crude reaction mixtures were analysed by their ^{1}H NMR spectra.

Table S5.

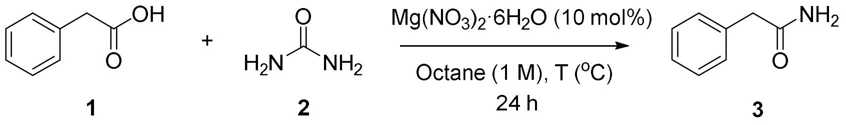

Entry	Urea (equiv.)	Conversion (%) 110 °C	Conversion (%) 120 °C
1	0.5	52	51
2	1	64	69
**3**	**2**	**72**	**93**
4	3	55	85

4.4. Optimisation of the catalyst loading

Following the general procedure described in section 3, a carousel tube was charged with the corresponding amount of $Mg(NO_3)_2{\cdot}6H_2O$, urea (2 equiv.) and octane (1 mL). The reaction mixture was stirred at 120 °C for 24 hours. After removal of the solvent *in vacuo*, the resulting crude reaction mixtures were analysed by their ^1H NMR spectra.

Table S6.

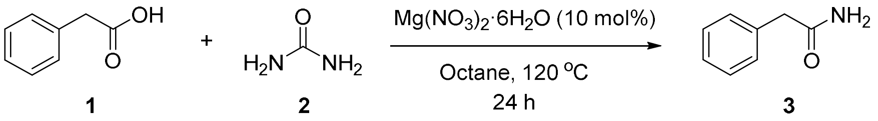

Entry	Catalyst loading (mol%)	Conversion (%)
1	5	69
**2**	**10**	**93**
3	15	94

4.5. Optimisation of the concentration

Following the general procedure described in section 3, a carousel tube was charged with $Mg(NO_3)_2{\cdot}6H_2O$ (10 mol%), urea (2 equiv.) and the corresponding amount of octane. The reaction mixture was stirred at 120 °C for 24 hours. After removal of the solvent *in vacuo*, the resulting crude reaction mixtures were analysed by their ^1H NMR spectra.

Table S7.

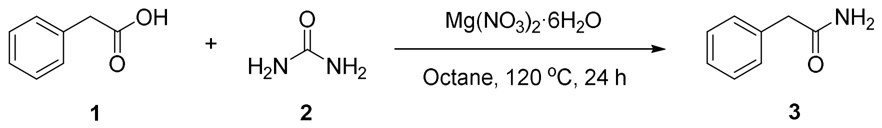

Entry	Concentration (M)	Conversion (%)
1	0.5	55
**2**	**1**	**78**
3	2	76
**4**	**neat**	**79**

4.6. General procedure for the synthesis of primary amides using $Mg(NO_3)_2 \cdot 6H_2O$

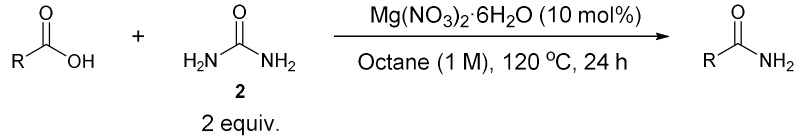

A carousel tube was charged with the acid species (3.0 mmol), urea (6.0 mmol), $Mg(NO_3)_2 \cdot 6H_2O$ (10 mol%) and octane (3 mL). The reaction mixture was stirred at 120 °C for 24 hours. The reaction was allowed to cool to room temperature, redissolved in methanol and the solvent was removed *in vacuo*. The resulting crude reaction was analysed by their ^1H NMR spectra using d^6-dmso as a solvent. When high conversions were achieved, the crude products were dissolved in ethyl acetate and washed with $NaHCO_3$ (3 x 10 mL). The organic layers were combined and dried over $MgSO_4$. The solvent was removed *in vacuo* on a rotary evaporator to yield the pure primary amides.

4.7. General procedure for the synthesis of *N*-methylamides from using *N,N'*- dimethyl urea

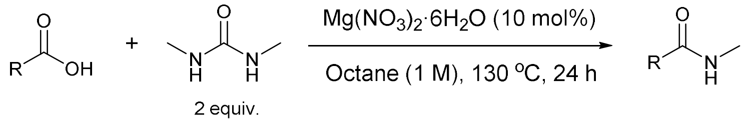

A carousel tube was charged with the acid species (3.0 mmol), *N,N'*-dimethylurea (6.0 mmol), $Mg(NO_3)_2 \cdot 6H_2O$ (10 mol%) and octane (3 mL). The reaction mixture was stirred at 130 °C for 24 hours. After being allowed to cool to room temperature, the crude reaction was redissolved in methanol and the solvent was removed *in vacuo*. The resulting crude reaction mixtures were analysed by their ^1H NMR spectra using d^6-dmso as solvent. The methylamides were purified by column chromatography using DCM:MeOH as eluent.

5. Optimisation of the Reaction Conditions using Imidazole as Catalyst

5.1. Imidazole and DMAP screen

Phenylacetic acid (136 mg, 1.0 mmol), urea (1.0, 1.5 or 2.0 mmol), the catalyst (10, 20 or 30 mol%) and octane (1 mL) were added to a carousel tube. The reaction mixture was stirred at 110 °C for 24 hours. After being allowed to cool to room temperature, the crude reaction was redissolved in

methanol and the solvent was removed *in vacuo*. The resulting crude reaction mixtures were analysed by their ^{1}H NMR spectra using d^{6}-dmso as a solvent.

Table S8.

Entry	Catalyst	Catalyst (mol%)	1 equiv. urea Conversion (%)	1.5 equiv. urea Conversion (%)	2 equiv. urea Conversion (%)
1	Background	0	24	24	25
2	Imidazole	10	59	71	72
3	Imidazole	20	78	**86**	84
4	Imidazole	30	--	85	--
5	DMAP	10	58	74	71
6	DMAP	20	71	**84**	84

5.2. Nitrogen source screen

Phenylacetic acid (136 mg, 1.0 mmol), nitrogen source (1.5 mmol) and imidazole (14 mg, 0.2 mmol) were added to a carousel tube followed by octane (1 mL). The reaction mixture was stirred at 110 ^{o}C for 24 hours. After being allowed to cool to room temperature, the crude reaction was redissolved in methanol and the solvent was removed *in vacuo*. The resulting crude reaction mixtures were analysed by their ^{1}H NMR spectra using d^{6}-dmso as a solvent.

Table S9.

Entry	Nitrogen source	Conversion (%)
**1**	**Urea**	**86**
2	Ammonium chloride	0
3	Ammonium formate	19
4	Ammonium acetate	20
5	Ammonium iodide	0
6	Ammonium carbamate	13
7	Formamide	32

5.3. Solvent screen

Phenylacetic acid (136 mg, 1.0 mmol), urea (90 mg, 1.5 mmol), imidazole (14 mg, 0.2 mmol) and the solvent (1 mL) were added to a carousel tube. The reaction mixture was stirred at 80 ^{o}C for 24 hours. After removal of the solvent *in vacuo*, the resulting crude reaction mixtures were analysed by their ^{1}H NMR spectra.

Table S10.

Entry	Solvent	Conversion (%)
1	Water	0
2	1-Propanol	0
3	Ethanol	0
4	Ethyl acetate	0
**5**	**Octane**	**10**
6	Cyclohexane	8
7	Toluene	5
8	2-Methyltetrahydrofuran	0

5.4. Temperature screen

Phenylacetic acid (136 mg, 1.0 mmol), urea (90 mg, 1.5 mmol) and imidazole (14 mg, 0.2 mmol) and octane (1 mL) were added to a carousel tube. The reaction mixture was stirred at the corresponding temperature for 24 hours. After being allowed to cool to room temperature, the crude reaction was redissolved in methanol and the solvent was removed *in vacuo*. The resulting crude reaction mixtures were analysed by their ^1H NMR spectra using d^6-dmso as a solvent.

Table S11.

Entry	Temperature (°C)	Conversion (%)	Background Conversion (%)
1	80	10	3
2	90	27	7
3	100	50	15
4	110	86	24
**5**	**120**	**96**	**33**
6	126	96	37

5.5. Optimisation of the concentration

Phenylacetic acid (136 mg, 1.0 mmol), urea (90 mg, 1.5 mmol) and imidazole (14 mg, 0.2 mmol) and octane were added to a carousel tube. The reaction mixture was stirred at 120 °C for 24 hours. After

being allowed to cool to room temperature, the crude reaction was redissolved in methanol and the solvent was removed *in vacuo*. The resulting crude reaction mixtures were analysed by their ^{1}H NMR spectra using d^6-dmso as a solvent.

Table S12.

Entry	Concentration (M)	Conversion (%)	Background Conversion (%)
1	0.5	87	28
**2**	**1**	**98**	**33**
3	2	95	34

5.6. General procedure for the synthesis of primary amides using imidazole

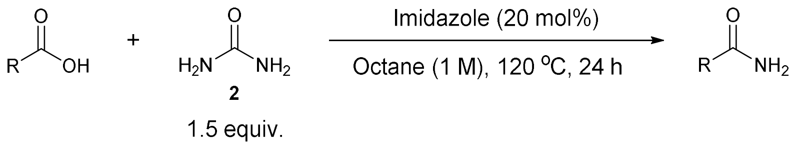

Carboxylic acid (3.0 mmol), urea (4.5 mmol) and imidazole (20 mol%) were added to a carousel tube followed by octane (3 mL), the reaction mixture was stirred at 120 °C for 24 hours. After being allowed to cool to room temperature, the crude reaction was redissolved in methanol and the solvent was removed *in vacuo*. The resulting crude reaction mixtures were analysed by their ^{1}H NMR spectra using d^6-dmso as a solvent. When high conversions were achieved, the reaction mixture was dissolved in ethyl acetate and extracted with 1 M solution of $NaHCO_3$ (x 2). The aqueous layers were combined and extracted with ethyl acetate. The organic fractions were combined and dried over $MgSO_4$. The solvent was removed *in vacuo* on a rotary evaporator to yield the pure primary amides.

5.7. Optimisation of the reaction conditions for the synthesis of secondary amides using imidazole

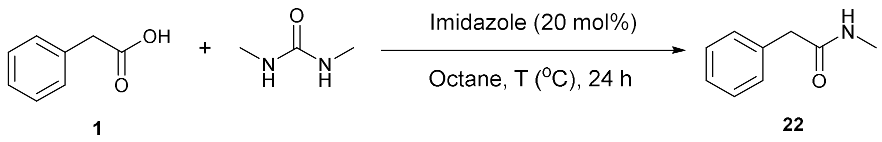

Phenylacetic acid (136 mg, 1.0 mmol), *N,N′*-dimethylurea, imidazole (14 mg, 0.2 mmol) and octane (1 mL) were added to a carousel tube. The reaction mixture was stirred at the corresponding temperature for 24 hours. After being allowed to cool to room temperature, the crude reaction was redissolved in methanol and the solvent was removed *in vacuo*. The resulting crude reaction mixtures were analysed by their ^{1}H NMR spectra using d^6-dmso as a solvent. Conversions were determined by comparison of the peaks at 3.46 (s, 2H, CH_2, **1**) and 3.43 (s, 2H, CH_2, **22**).

Table S13.

Entry	*N,N′*-Dimethylurea (equiv.)	Temperature (˚C)	Conversion (%)
1	1.5	120	54

2	1.5	130	83
**3**	**2**	**130**	**89**

5.8. General procedure for the synthesis of secondary amides using imidazole

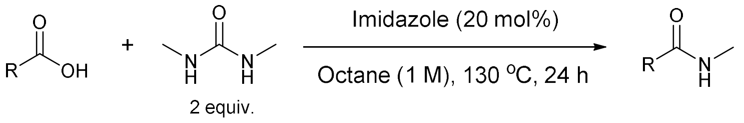

Carboxylic acid (3.0 mmol), *N,N'*-dimethylurea (6.0 mmol), imidazole (20 mol%) and octane (3 mL) were added to a carousel tube. The reaction mixture was stirred at 130 °C for 24 hours. After being allowed to cool to room temperature, the crude reaction was redissolved in methanol and the solvent was removed *in vacuo*. The resulting crude reaction mixtures were analysed by their ^{1}H NMR spectra using d^6-dmso as a solvent. When high conversions were achieved, the reaction mixture was dissolved in ethyl acetate and extracted with 1 M solution of $NaHCO_3$ (x 2). The aqueous layers were combined and extracted with ethyl acetate. The organic fractions were combined and dried over $MgSO_4$. The solvent was removed *in vacuo* on a rotary evaporator to yield the pure secondary amides.

6. Use of Other Ureas for the Synthesis of Amides

6.1. Use of *N,N,N',N'*-tetramethylurea

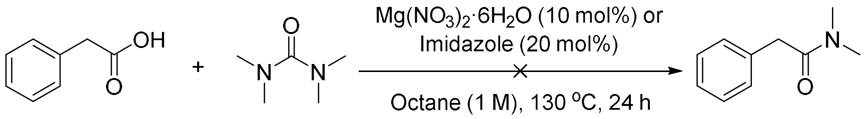

1 **39** **40**

Phenylacetic acid (136 mg, 1.0 mmol), *N,N,N',N'*-tetramethylurea (232 mg, 2.0 mmol) and imidazole (14 mg, 0.2 mmol) or $Mg(NO_3)_2·6H_2O$ (26 mg, 0.1 mmol) and octane (1 mL) were added to a carousel tube. The reaction mixture was stirred at 130 °C for 24 hours. After being allowed to cool to room temperature, the crude reaction was redissolved in methanol and the solvent was removed *in vacuo*. The resulting crude reaction mixtures were analysed by their ^{1}H NMR spectra using d^6-dmso as a solvent showing no conversion into *N,N*-dimethyl-2-phenylacetamide.

6.2. Use of *N*-methylurea

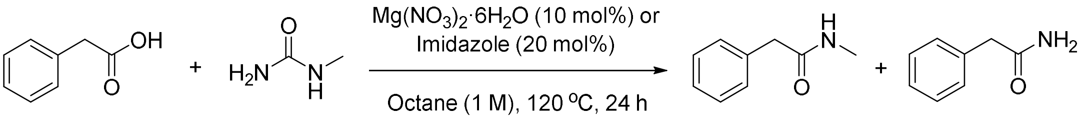

1 **41** **22** **3**

Phenylacetic acid (136 mg, 1.0 mmol), *N*-methylurea (148 mg, 2.0 mmol), imidazole (14 mg, 0.2 mmol) or $Mg(NO_3)_2·6H_2O$ (26 mg, 0.1 mmol) and octane (1 mL) were added to a carousel tube. The reaction mixture was stirred at 120 °C for 24 hours. After being allowed to cool to room temperature, the crude reaction was redissolved in methanol and the solvent was removed *in vacuo*. The resulting crude reaction mixtures were analysed by their ^{1}H NMR spectra using d^6-dmso. Conversions were determined by comparison of the peaks at 3.51 (s, 2H, CH_2, **1**), 3.40 (s, 2H, CH_2, **22**) and 3.38 (s, 2H, CH_2, **3**).

Table S14.

Entry	Catalyst	Conversion (%)	
		**22**	**3**
1	--	40	5
2	$Mg(NO_3)_2{\cdot}6H_2O$	66	14
3	Imidazole	77	10

6.3. Use of *N,N*-dimethylurea

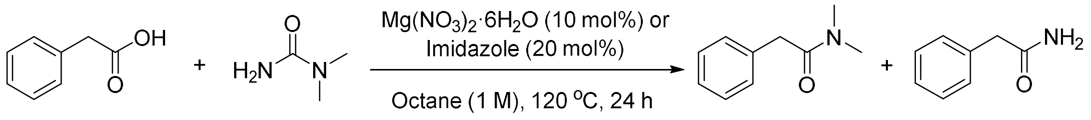

1 **42** **40** **3**

Phenylacetic acid (136 mg, 1.0 mmol), *N*,*N*-dimethylurea (176 mg, 2.0 mmol) and imidazole (14 mg, 0.2 mmol) or $Mg(NO_3)_2{\cdot}6H_2O$ (26 mg, 0.1 mmol) and octane (1 mL) were added to a carousel tube. The reaction mixture was stirred at 120 °C for 24 hours. After being allowed to cool to room temperature, the crude reaction was redissolved in methanol and the solvent was removed *in vacuo*. The resulting crude reaction mixtures were analysed by their ^{1}H NMR spectra using d^6-dmso. Conversions were determined by comparison of the peaks at 3.49 (s, 2H, CH_2, **1**), 3.46 (s, 2H, CH_2, **40**) and 3.38 (s, 2H, CH_2, **3**).

Table S15.

Entry	Catalyst	Conversion (%)	
		**40**	**3**
1	--	36	3
2	$Mg(NO_3)_2{\cdot}6H_2O$	65	--
3	Imidazole	75	14

7. Mechanistic Insights

7.1. Decomposition of urea

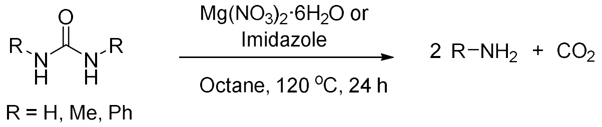

Urea (1.5 mmol), $Mg(NO_3)_2{\cdot}6H_2O$ (0.1 mmol) or imidazole (0.2 mmol) and octane (1 mL) were added to a carousel tube. The reaction mixture was stirred at 120 °C for 24 hours. After being allowed to cool to room temperature, the crude reaction was redissolved in methanol and the solvent was removed *in vacuo*. The resulting crude reaction mixtures were analysed by their ^{1}H NMR spectra using d^6-dmso. The analysis of the reaction crude by ^{1}H NMR did not show the formation of any primary amide and only the urea was detected. Gravimetric analysis before and after the reaction showed a mass recovery of over 92%.

7.2. Direct amidation using aniline[1]

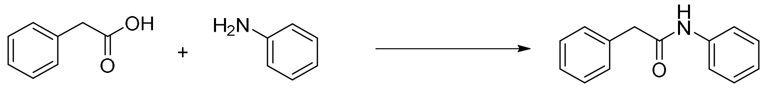

1 **37** **33**

Phenylacetic acid (136 mg, 1.0 mmol), aniline (182 µL, 2.0 mmol), $Mg(NO_3)_2{\cdot}6H_2O$ (26 mg, 0.1 mmol) or imidazole (14 mg, 0.2 mmol) and octane (1 mL) were added to a carousel tube. The reaction mixture was stirred at 120 °C for 24 hours. After being allowed to cool to room temperature, the crude reaction was redissolved in methanol and the solvent was removed *in vacuo*. The resulting crude reaction mixtures were analysed by their ^{1}H NMR spectra using d^6-dmso. Conversions were determined by analysis of their ^{1}H NMR spectra by comparison of the peaks at 3.70 (s, 2H, CH_2, **33**) and 3.60 (s, 2H, CH_2, **1**).

Table S16.

Entry	Catalyst	Conversion (%)	Conversion (%) No catalyst
1	$Mg(NO_3)_2{\cdot}6H_2O$	77	75
2	Imidazole	70	71

7.3. Synthesis of *N*-carbamoylpivalamide[2]

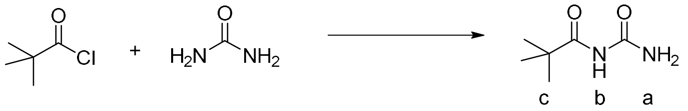

2 **43**

A solution of trimethylacetyl chloride (1.1 mL, 10.0 mmol) in dry acetonitrile (20 mL) was added to a boiling solution of urea (2.4 g, 40.0 mmol) in dry acetonitrile (30 mL) under an atmosphere of argon. The reaction was heated at reflux for 2 hours. The reaction mixture was allowed to cool to room temperature and the solvent was removed *in vacuo*. The resulting mixture was dissolved in ethyl acetate (15 mL) and extracted with water (3 x 20 mL). The aqueous layers were combined and

extracted with ethyl acetate (3 x 20 mL). The organic fractions were combined, dried over $MgSO_4$, filtered and the solvent removed *in vacuo* to give **43** as a white solid (1.1 g, 75%).

The spectroscopic data was consistent with those reported.[2]

^{1}H NMR (500 MHz, $CDCl_3$) δ 8.35 (br s, 2H, a), 5.64 (br s, 1H, b), 1.26 (s, 9H, c); ^{13}C NMR (125 MHz, $CDCl_3$) δ 180.1, 155.3, 40.0, 26.3; HRMS calcd for $[C_6H_{13}N_2O_2]^+$: 145.0977 $[M+H]^+$, found: 145.0996; m.p. 147-149 °C [lit. m.p. 151-153 °C].[2]

7.4. Synthesis of *N*-carbamoylphenylacetamide[3]

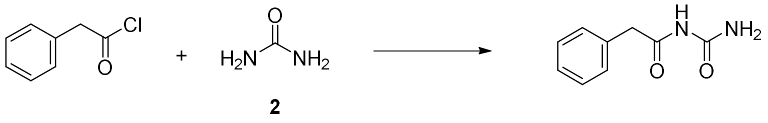

A solution of phenylacetyl chloride (1.32 mL, 10.0 mmol) in dry acetonitrile (20 mL) was added to a boiling solution of urea (2.4 g, 40.0 mmol) in dry acetonitrile (30 mL) under an atmosphere of argon. The reaction was heated at reflux for 2 hours. The reaction mixture was allowed to cool to room temperature and the solvent was removed *in vacuo*. The resulting mixture was dissolved in ethyl acetate (15 mL) and extracted with water (3 x 20 mL). The aqueous layers were combined and extracted with ethyl acetate (3 x 20 mL). The organic fractions were combined, dried over $MgSO_4$, filtered and the solvent removed *in vacuo* to give the desired product as a white solid (1.67 mg, 94%).

^{1}H NMR (300 MHz, d^6-DMSO) δ 10.41 (br s, 1H, NH), 7.69 (br s, 1H, NH_2), 7.34-7.22 (m, 6H, Ar, NH_2), 3.60 (s, 2H, CH_2); ^{13}C NMR (75.5 MHz, d^6-DMSO) δ 172.8, 154.0, 134.8, 129.3, 128.4, 126.9, 42.5; HRMS calcd for $[C_9H_{11}N_2O_2]^+$: 179.0820 $[M+H]^+$, found: 179.0816; m.p. 218-220 °C [lit. m.p. 211-213 °C].[4]

7.5. Formation of amides from the *N*-acylurea intermediates

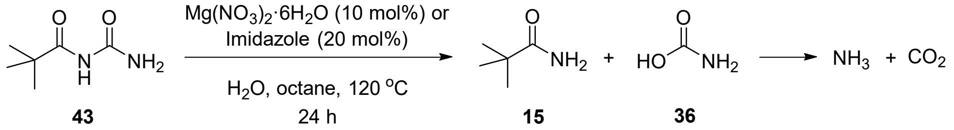

N-Carbamoylpivalamide (144 mg, 1.0 mmol), water (36 µL, 2.0 mmol), $Mg(NO_3)_2{\cdot}6H_2O$ (26 mg, 0.1 mmol) or imidazole (14 mg, 0.2 mmol) and octane (1 mL) were added to a carousel tube. The reaction mixture was stirred at 120 °C for 24 hours. After being allowed to cool to room temperature, the crude reaction was redissolved in methanol and the solvent was removed *in vacuo*. The resulting crude reaction mixtures were analysed by their ^{1}H NMR spectra using d^6-dmso. Conversions were determined by analysis of their ^{1}H NMR spectra by comparison of the peaks at 1.15 (s, 9H, $(CH_3)_3$, **43**) and 1.07 (s, 9H, $(CH_3)_3$, **15**).

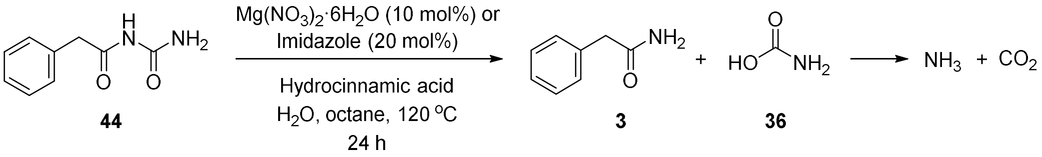

Phenylacetylurea (89 mg, 0.5 mmol), water (18 µL, 1 mmol), hydrocinnamic acid (75 mg, 0.5 mmol), $Mg(NO_3)_2{\cdot}6H_2O$ (13 mg, 0.05 mmol) or imidazole (7 mg, 0.1 mmol) and octane (0.5 mL) were added to a carousel tube. The reaction mixture was stirred at 120 °C for 24 hours. After being allowed to cool to room temperature, the crude reaction was redissolved in methanol and the solvent was removed *in vacuo*. The resulting crude reaction mixtures were analysed by their ^{1}H NMR spectra using d^6-dmso.

Conversions determined by analysis of their ^{1}H NMR spectra by comparison of the peaks at 3.60 (s, 2H, CH_2, **44**) and 3.36 (s, 2H, CH_2, **3**).

Table S17.

Entry	Substrate	$Mg(NO_3)_2 \cdot 6H_2O$ (10 mol%)	Imidazole (20 mol%)	Water (2 equiv)	Conversion (%)
1	**44**	√	x	√	27
2	**44**	x	√	√	18
3	**43**	√	x	√	25
4	**43**	x	√	√	14

8. Synthesis of Primary Amides

2-Phenylacetamide (3)[5]

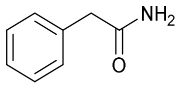

Using $Mg(NO_3)_2 \cdot 6H_2O$ as catalyst. Following the general procedure described in section 4.6, phenylacetic acid (408 mg, 3.0 mmol) was used as the acid species. The *title* compound was recovered after purification as an off-white solid (393 mg, 97%).

Using imidazole as catalyst. Following the general procedure described in section 5.6, phenylacetic acid (408 mg, 3.0 mmol) was used as the acid species. The *title* compound was recovered after purification as an off-white solid (367 mg, 91%)

The spectroscopic data obtained using both methods was consistent with those reported.[5]

^{1}H NMR (300 MHz, $CDCl_3$) δ 7.40 – 7.27 (m, 5H, Ar), 5.65 (br s, 1H, NH), 5.39 (br s, 1H, NH), 3.59 (s, 2H, CH_2); ^{13}C NMR (75 MHz, $CDCl_3$) δ 173.6, 134.8, 129.4, 129.0, 127.6, 43.3; FT-IR (neat) υ in cm^{-1}: 1627 (C=O stretch).

Using $Mg(NO_3)_2 \cdot 6H_2O$ as catalyst. HRMS-ESI calcd for $[C_8H_9NONa]^+$: 158.0581 $[M+Na]^+$, found 158.0591; m.p. 152 – 155 °C (lit. m.p. 155 – 158 °C).[5]

Using imidazole as catalyst. HRMS-ESI calcd for $[C_8H_{10}NO]^+$: 136.0762 $[M+H]^+$, found 136.0781; m.p. 157 – 159 °C (lit. m.p. 155 – 158 °C).[5]

4-Methoxyphenylacetamide (4)[6,7]

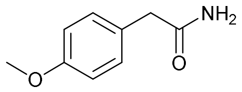

Using $Mg(NO_3)_2{\cdot}6H_2O$ as catalyst. Following the general procedure described in section 4.6, 4-methoxyphenylacetic acid (498 mg, 3.0 mmol) was used as the acid species. The *title* compound was recovered after purification as a yellow solid (431 mg, 87%).

Using imidazole as catalyst. Following the general procedure described in section 5.6, 4-methoxyphenylacetic acid (498 mg, 3.0 mmol) was used as the acid species. The *title* compound was recovered after purification as a yellow solid (450 mg, 91%).

The spectroscopic data obtained using both methods was consistent with those reported.[6]

^{1}H NMR (500 MHz, d^6-DMSO) δ 7.37 (br s, 1H, NH_2), 7.16 (d, 2H, *J* = 10.0 Hz, Ar), 6.85 (d, 2H, *J* = 10.0 Hz, Ar), 6.80 (br s, 1H, NH_2), 3.72 (s, 3H, CH_3), 3.26 (s, 2H, CH_2); ^{13}C NMR (125 MHz, d^6-DMSO) δ 172.9, 158.2, 130.4, 128.8, 114.0, 55.4, 41.4; FT-IR (neat) υ in cm^{-1}: 1627 (C=O stretch).

Using $Mg(NO_3)_2{\cdot}6H_2O$ as catalyst. HRMS-ESI calcd for $[C_9H_{12}NO_2]^+$: 166.0868 $[M+H]^+$, found 166.0873; m.p. 158 – 160 °C (lit. m.p. 164 – 166 °C).[7]

Using imidazole as catalyst. HRMS-ESI calcd for $[C_9H_{12}NO_2]^+$: 166.0868 $[M+H]^+$, found 166.0878; m.p. 158 – 160 °C (lit. m.p. 164 – 166 °C).[7]

(1,3-Benzodioxyl-5-ylmethyl)-amide (5)[8,9]

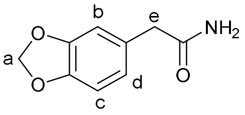

Using $Mg(NO_3)_2{\cdot}6H_2O$ as catalyst. Following the general procedure described in section 4.6, 2-(1,3-benzodioxyl-5-yl)-acetic acid (180 mg, 1.0 mmol) was used as the acid species. The *title* compound was recovered after purification as a white solid (154 mg, 86%).

Using imidazole as catalyst. Following the general procedure described in section 5.6, 3,4-(methylenedioxy)phenylacetic acid (180 mg, 1.0 mmol) was used as the acid species. The *title* compound was recovered after purification as a white solid (174 mg, 97%).

The spectroscopic data obtained using both methods was consistent with those reported.[8,9]

^{1}H NMR (500 MHz, d^6-DMSO) δ 7.36 (s, 1H, NH_2), 6.82 (m, 3H, b, d, NH_2), 6.70 (d, *J* = 7.8 Hz, 1H, c), 5.96 (s, 2H, a) 3.27 (s, 2H, e); ^{13}C NMR (125 MHz, d^6-DMSO) δ 172.3, 147.0, 145.7, 130.1, 122.0, 109.5, 107.9, 100.7, 41.8; FT-IR (neat) υ in cm^{-1}: 1644 (C=O stretch).

Using $Mg(NO_3)_2{\cdot}6H_2O$ as catalyst. HRMS calcd for $[C_9H_{10}NO_3]^+$: 180.0661 $[M+H]^+$, found: 180.0668; m.p. 174 – 176 °C (lit. m.p. 172 – 173 °C).[9]

Using imidazole as catalyst. HRMS calcd for $[C_9H_{10}NO_3]^+$: 180.0661 $[M+H]^+$, found: 180.0668; m.p. 173 – 175 °C (lit. m.p. 172 – 173 °C).[9]

4-Chlorophenylacetamide (6)[6]

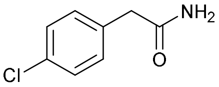

Using $Mg(NO_3)_2{\cdot}6H_2O$ as catalyst. Following the general procedure described in section 4.6, 4-chlorophenylacetic acid (510 mg, 3.0 mmol) was used as the acid species. The *title* compound was recovered after purification as a white solid (410 mg, 81%).

Using imidazole as catalyst. Following the general procedure described in section 5.6, 4-chlorophenylacetic acid (512 mg, 3.0 mmol) was used as the acid species. The *title* compound was recovered after purification as a white solid (487 mg, 97%).

The spectroscopic data obtained using both methods was consistent with those reported.[6]

^{1}H NMR (300 MHz, d^6-DMSO) δ 7.52 (br s, 1H, NH_2), 7.37 (d, *J* = 9.0 Hz, 2H, Ar), 7.28 (d, *J* = 9.0 Hz, 2H, Ar), 6.95 (br s, 1H, NH_2), 3.36 (s, 2H, CH_2); ^{13}C NMR (125 MHz, d^6-DMSO) δ 172.1, 135.3, 128.4, 41.7; FT-IR (neat) υ in cm^{-1}: 1627 (C=O stretch).

Using $Mg(NO_3)_2 \cdot 6H_2O$ as catalyst. HRMS-ESI calcd for $[C_8H_9ClNO]^+$: 170.0373 [M+H]$^+$, found 170.0380; m.p 178 – 180 °C (lit. m.p. 179 – 182 °C).[10]

Using imidazole as catalyst. HRMS-ESI calcd for $[C_8H_8ClNONa]^+$: 192.0192, found 192.0188; m.p. 183 – 185 °C (lit. m.p. 179 – 182 °C).[10]

Diphenylacetamide (7)[11]

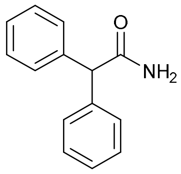

Using $Mg(NO_3)_2 \cdot 6H_2O$ as catalyst. Following the general procedure described in section 4.6, diphenylacetic acid (633 mg, 3.0 mmol) was used as the acid species. The *title* compound was recovered after purification as a white solid (580 mg, 92%).

Using imidazole as catalyst. Following the general procedure described in section 5.6, diphenylacetic acid (409 mg, 3.0 mmol) was used as the acid species. The *title* compound was recovered after purification as a white solid (264 mg, 65%).

The spectroscopic data obtained using both methods was consistent with those reported.[11]

^{1}H NMR (300 MHz, $CDCl_3$) δ 7.37 – 7.24 (m, 10H, Ar), 5.75 (br s, 1H, NH_2), 5.58 (br s, 1H, NH_2), 4.97 (s, 1H, CH); ^{13}C NMR (75 MHz, $CDCl_3$) δ 174.9, 139.1, 128.8, 127.3, 58.7; FT-IR (neat) υ in cm^{-1} = 1646 (C=O stretch).

Using $Mg(NO_3)_2 \cdot 6H_2O$ as catalyst. HRMS-ESI calcd for $[C_{14}H_{14}NO]^+$: 212.1075 [M+H]$^+$, found 212.1081; m.p. 166 – 168 °C (lit. m.p. 169 °C).[12]

Using imidazole as catalyst. HRMS-ESI calcd for $[C_{14}H_{13}NONa]^+$: 234.0865 [M+Na]$^+$, found 234.0903; m.p. 166 – 168 °C (lit. m.p. 169 °C).[12]

3-Phenylpropionamide (8)[13]

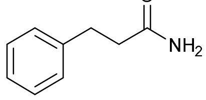

Using $Mg(NO_3)_2 \cdot 6H_2O$ as catalyst. Following the general procedure described in section 4.6, 3-phenylpropionic acid (450 mg, 3 mmol) was used as the acid species. The *title* compound was recovered after purification as an off-white solid (402 mg, 90%).

Using imidazole as catalyst. Following the general procedure described in section 5.6, hydrocinnamic acid (450 mg, 3 mmol) was used as the acid species. The *title* compound was recovered after purification as an off-white solid (433 mg, 97%).

The spectroscopic data obtained using both methods was consistent with those reported.[13]

^{1}H NMR (300 MHz, $CDCl_3$) δ 7.36 – 7.22 (m, 5H, Ar), 5.51 (br s, 1H, NH_2), 5.39 (br s, 1H, NH_2), 3.01 (t, 2H, J = 6.0 Hz, $PhCH_2$), 2.56 (t, 2H, J = 6.0 Hz, CH_2CO); ^{13}C NMR (75 MHz, $CDCl_3$) δ 174.6, 140.6, 128.5, 128.3, 126.3, 37.5, 31.4; FT-IR (neat) υ in cm^{-1}: 1626 (C=O stretch).

Using $Mg(NO_3)_2·6H_2O$ as catalyst. HRMS-ESI calcd for $[C_9H_{12}NO]^+$: 150.0919 $[M+H]^+$, found 150.0933; m.p. 98 – 100 °C (lit. m.p. 102 – 104 °C).[13]

Using imidazole as catalyst. HRMS calcd for $[C_9H_{12}NO]^+$: 150.0919 $[M+H]^+$, found 150.0941; m.p. 101 – 103 °C (lit. m.p. 102 – 104 °C).[13]

3-(4'-Methoxyphenyl)propionamide (9)[14]

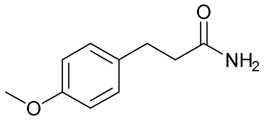

Using $Mg(NO_3)_2·6H_2O$ as catalyst. Following the general procedure described in section 4.6, 3-(4-methoxyphenyl)propionic acid (540 mg, 3 mmol) was used as the acid species. The *title* compound was recovered after purification as a white solid (488 mg, 91%).

Using imidazole as catalyst. Following the general procedure described in section 5.6, 3-(4-methoxyphenyl)propionic acid (540 mg, 3 mmol) was used as the acid species. The *title* compound was recovered after purification as a white solid (483 mg, 90%).

The spectroscopic data obtained using both methods was consistent with those reported.[14]

^{1}H NMR (300 MHz, $CDCl_3$) δ 7.15 (d, 2H, J = 9.0 Hz, Ar), 6.84 (d, 2H, J = 9.0 Hz, Ar), 5.52 (br s, 1H, NH_2), 5.37 (br s, 1H, NH_2), 3.79 (s, 3H, CH_3), 2.92 (t, 2H, J = 6.0 Hz, $PhCH_2$), 2.50 (t, 2H, J = 6.0 Hz, CH_2CO); ^{13}C NMR (125 MHz, $CDCl_3$) δ 174.8, 158.0, 132.7, 129.2, 113.9, 55.2, 37.8, 30.5; FT-IR (neat) υ in cm^{-1}: 1643 (C=O stretch).

Using $Mg(NO_3)_2·6H_2O$ as catalyst. HRMS-ESI calcd for $[C_{10}H_{13}NO_2Na]^+$: 202.0844 $[M+Na]^+$, found 202.0840; m.p. 123 – 124 °C (lit. m.p. 123 – 124 °C).[15]

Using imidazole as catalyst. HRMS-ESI calcd for $[C_{10}H_{14}NO_2]^+$: 180.1024 $[M+H]^+$, found 180.1039; m.p. 122 – 123 °C (lit. m.p. 123 – 124 °C).[15]

Benzamide (10)[5]

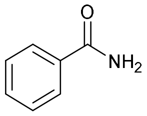

Using $Mg(NO_3)_2·6H_2O$ as catalyst. Following the general procedure described in section 4.6, benzoic acid (366 mg, 3 mmol) gave the corresponding amide **10** in 18% conversion. Conversion was determined by analysis of the ^{1}H NMR spectrum by comparison of the peaks at 7.89 (s, 2H, CH_2, starting material) and 7.86 (s, 2H, CH_2, **10**).

Using imidazole as catalyst. Following the general procedure described in section 5.6, benzoic acid (366 mg, 3 mmol) gave the corresponding amide **10** in 30% conversion.

4-Chlorobenzamide (11)[5]

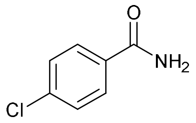

Using $Mg(NO_3)_2 \cdot 6H_2O$ as catalyst. Following the general procedure described in section 4.6, 4-chlorobenzoic acid (470 mg, 3.0 mmol) did not give the corresponding amide **11**.

Using imidazole as catalyst. Following the general procedure described in section 5.6, 4-chlorobenzoic acid (470 mg, 3 mmol) gave the corresponding amide **11** in 18% conversion. Conversion was determined by analysis of ^{1}H NMR spectrum by comparison of the peaks at 7.51 (d, 2H, CH_2, **11**) and 7.46 (d, 2H, CH_2, starting material).

4-Nitrobenzamide (12)

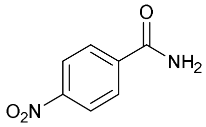

Using $Mg(NO_3)_2 \cdot 6H_2O$ as catalyst. Following the general procedure described in section 4.6, 4-nitrobenzoic acid (500 mg, 3.0 mmol) gave the corresponding amide **12** in 17% conversion. Conversion was determined by analysis of ^{1}H NMR spectrum by comparison of the peaks at 7.51 (d, 2H, CH_2, **12**) and 7.46 (d, 2H, CH_2, starting material).

Using imidazole as catalyst. Following the general procedure described in section 5.6, 4-nitrobenzoic acid (500 mg, 3 mmol) gave the corresponding amide **12** in 18% conversion.

Benzoylamidoacetamide (13)[16,17]

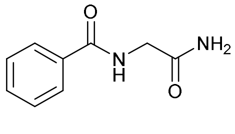

Using $Mg(NO_3)_2 \cdot 6H_2O$ as catalyst. Following the general procedure described in section 4.6, benzoylaminoacetic acid (358 mg, 2.0 mmol) was used as the acid species. The *title* compound was recovered after purification by column chromatography (eluting with EtOAc:Hexane 1:1) as an off-white solid (280 mg, 80%).

Using imidazole as catalyst. Following the general procedure described in section 5.6, benzoylaminoacetic acid (538 mg, 3.0 mmol) was used as the acid species. The *title* compound was recovered after purification by column chromatography (eluting with EtOAc:Hexane 1:1) as an off-white solid (460 mg, 86%).

The spectroscopic data obtained was consistent with those reported.[16]

^{1}H NMR (500 MHz, d^6-DMSO) δ 8.65 (t, 1H, *J* = 5.0 Hz, NH), 7.88 (d, 2H, *J* = 10.0 Hz, Ar), 7.53 (t, 1H, *J* = 10.0 Hz, Ar), 7.47 (t, 2H, *J* = 5.0 Hz, Ar), 7.36 (br s, 1H, NH_2), 7.03 (br s, 1H, NH_2), 3.82 (d, 2H, *J* = 5.0 Hz, CH_2); ^{13}C NMR (125 MHz, d^6-DMSO) δ 171.0, 166.3, 134.1, 131.2, 128.2, 127.3, 42.4; FT-IR (neat) υ in cm^{-1} = 1627 (C=O stretch).

Using $Mg(NO_3)_2{\cdot}6H_2O$ as catalyst. HRMS-ESI calcd for $[C_9H_{10}N_2O_2Na]^+$: 201.0640 [M+Na]$^+$, found 201.0614; m.p. 140 – 143 °C (lit. m.p. 139 – 141 °C).[17]

Using imidazole as catalyst. HRMS-ESI calcd for $[C_9H_{10}N_2O_2Na]^+$: 201.0640 [M+Na]$^+$, found 201.0638; m.p. 139 – 142 °C (lit. m.p. 139 – 141 °C).[17]

Hexanamide (14)[18]

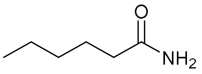

Using $Mg(NO_3)_2{\cdot}6H_2O$ as catalyst. Following the general procedure described in section 4.6, hexanoic acid (376 µL, 3.0 mmol) was used as the acid species. The *title* compound was recovered after purification as a white solid (300 mg, 87%).

Using imidazole as catalyst. Following the general procedure described in section 5.6, hexanoic acid (376 µL, 3.0 mmol) was used as the acid species. The *title* compound was recovered after purification as a white solid (316 mg, 89%)

The spectroscopic data obtained using both methods was consistent with those reported.[18]

^{1}H NMR (300 MHz, $CDCl_3$) δ 5.41 (br s, 2H, NH_2), 2.22 (t, 2H, *J* = 6.0 Hz, CH_2CO), 1.69 – 1.59 (m, 2H, $\underline{CH_2}$-CH_2CO), 1.37 – 1.27 (m, 4H, $\underline{CH_2}$-$\underline{CH_2}$-CH_2-CH_2CO), 0.9 (t, 3H, *J* = 6.0 Hz, CH_3); ^{13}C NMR (125 MHz, $CDCl_3$) δ 176.4, 36.2, 31.7, 25.5, 22.7, 14.2; FT-IR (neat) υ in cm^{-1}: 1631 (C=O stretch)

Using $Mg(NO_3)_2{\cdot}6H_2O$ as catalyst. HRMS-MS calcd for $[C_6H_{14}NO]^+$: 116.1075 [M+H]$^+$, found 116.1092; m.p. 98 °C (lit. m.p. 101 – 102 °C).[18]

Using imidazole as catalyst. HRMS calcd for $[C_6H_{13}NONa]^+$: 138.0894 [M+Na]$^+$, found 138.0908; m.p 98 – 100 °C (lit. m.p. 101 – 102 °C).[18]

Trimethylacetamide (15)[19,20]

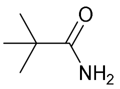

Using $Mg(NO_3)_2{\cdot}6H_2O$ as catalyst. Following the general procedure described in section 4.6, trimethylacetic acid (115 µL, 1.0 mmol) was used as acid species. The *title* compound was recovered after purification by column chromatography (eluting with EtOAc:Hexane 4:1) as a yellowish solid (56 mg, 55%).

Using imidazole as catalyst. Following the general procedure described in section 4.6, trimethylacetic acid (115 µL, 1.0 mmol) was used as acid species. The title compound was recovered after purification as yellowish solid (60 mg, 60%).

The spectroscopic data obtained using both methods was consistent with those reported.[19]

^{1}H NMR (500 MHz, DMSO-d_6) δ 6.99 (br s, 1H, NH_2), 6.67(br s, 1H, NH_2), 1.07 (s, 9H, CH_3); ^{13}C NMR (126 MHz, $CDCl_3$) δ 179.7, 37.8, 27.5; FT-IR (neat) υ in cm^{-1}: 1652 (C=O stretch)

Using $Mg(NO_3)_2·6H_2O$ as catalyst. HRMS-MS calcd for $[C_6H_{14}NO]^+$: 102.0919 $[M+H]^+$, found 102.0921; m.p. 154 – 156 °C (lit. m.p. 156 – 157 °C).[20]

Using imidazole as catalyst. HRMS calcd for $[C_6H_{13}NONa]^+$: 102.0919 $[M+NH]^+$, found 102.0917; m.p 155 – 157 °C (lit. m.p. 156 – 157 °C).[20]

Oleamide (16)[21,22]

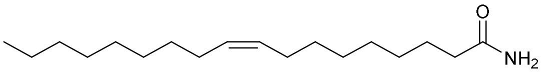

Using $Mg(NO_3)_2·6H_2O$ as catalyst. Following the general procedure described in section 4.6, oleic acid (634 µL, 2.0 mmol) was used as the acid species. The crude reaction mixture was dissolved in DCM and washed with NaOH (3 x 15 mL). The organic layers were then combined and dried over $MgSO_4$. The solvent was then removed *in vacuo*. The *title* compound was recovered as an off-white solid (475 mg, 85%).

Using imidazole as catalyst. Following the general procedure described in section 5.6, oleic acid (952 µL, 3.0 mmol) was used as the acid species. The *title* compound was recovered after purification as an off-white solid (768 mg, 91%).

The spectroscopic data obtained using both methods was consistent with those reported.[21]

^{1}H NMR (500 MHz, d^6-DMSO) δ 7.21 (s, 1H, NH_2), 6.67 (s, 1H, NH_2), 5.35 – 5.28 (m, 2H, CH=), 2.02 – 1.96 (m, 6H, CH_2), 1.48 – 1.42 (m, 2H, CH_2), 1.29 – 1.24 (m, 20H, CH_2), 0.85 (t, 3H, *J* = 5.0 Hz, CH_3); ^{13}C NMR (126 MHz, DMSO-d_6) δ 174.20, 129.56, 129.56, 35.10, 31.28, 29.14, 29.10, 28.84, 28.74, 28.72, 28.69, 28.60, 28.59, 26.61, 26.57, 25.10, 22.09, 13.90; FT-IR (neat) υ in cm^{-1} = 1632 (C=O stretch).

Using $Mg(NO_3)_2·6H_2O$ as catalyst. HRMS-ESI calcd for $[C_{18}H_{36}NO]^+$: 282.2797 $[M+H]^+$, found 282.2715; m.p. 72 – 75 °C (lit. m.p. 71 – 73 °C).[22]

Using imidazole as catalyst. HRMS-ESI calcd for $[C_{18}H_{36}NO]^+$: 282.2797 $[M+H]^+$, found 282.2772; m.p. 70 – 72 °C (lit. m.p. 71 – 73 °C).[22]

4-Pentenamide (17)[23]

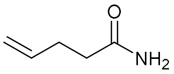

Using $Mg(NO_3)_2·6H_2O$ as catalyst. Following the general procedure described in section 4.6, 4-pentenoic acid (300 µL, 3.0 mmol) was used as the acid species. The *title* compound was recovered after purification as a white solid (282 mg, 85%).

The spectroscopic data obtained was consistent with those reported.[23]

^{1}H NMR (500 MHz, $CDCl_3$) δ 5.89 – 5.81 (m, CH_2=<u>CH</u>), 5.48 (br s, 2H, NH_2), 5.12 – 5.02 (m, 2H, <u>CH_2</u>=CH), 2.43 – 2.38 (m, 2H, CH_2CO), 2.34 – 2.31 (m, 2H, <u>CH_2</u>CH=); ^{13}C NMR (125 MHz, $CDCl_3$) δ 175.2, 137.2, 116.2, 35.5, 29.6; FT-IR (neat) υ in cm^{-1}: 1663 (C=O stretch), 1629 (C=C stretch); HRMS-ESI calcd for $[C_5H_{10}NO]^+$: 100.0762 $[M+H]^+$, found = 100.0762; m.p. 100 – 101 °C (lit. m.p. 105 – 106 °C).[23]

***trans*-Cinnamamide (19)**[5,24]

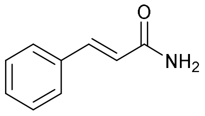

Using $Mg(NO_3)_2·6H_2O$ as catalyst. Following the general procedure described in section 4.6, *trans*-cinnamic acid (444 mg, 3.0 mmol) was used as the acid species. The *title* compound was recovered after purification as a white solid (265 mg, 60%).

Using imidazole as catalyst. Following the general procedure described in section 5.6, *trans*-cinnamic acid (952 µL, 3.0 mmol) was used as the acid species. The *title* compound was recovered after purification as a white solid (230 mg, 52%).

The spectroscopic data obtained using both methods was consistent with those reported.[24]

^{1}H NMR (500 MHz, DMSO-d_6) δ 7.61 – 7.49 (m, 3H, NH_2, Ar), 7.47 – 7.34 (m, 4H, Ar, CH=), 7.10 (br s, 1H, NH_2), 6.61 (d, *J* = 15.9 Hz, 1H, =CHCO); ^{13}C NMR (100 MHz, DMSO-d_6) δ 166.6, 139.1, 134.9, 129.4, 128.8, 127.5, 122.3; FT-IR (neat) υ in cm^{-1} = 1659 (C=O stretch), 1602 (C=C stretch).

Using $Mg(NO_3)_2·6H_2O$ as catalyst. HRMS-ESI calcd for $[C_9H_{10}NO]^+$: 148.0762 $[M+H]^+$, found 148.0766; m.p. 149 – 151 °C (lit. m.p. 148 – 151 °C).[5]

Using imidazole as catalyst. HRMS-ESI calcd for $[C_9H_{10}NO]^+$: 148.0762 $[M+H]^+$, found 148.0760; m.p. 149 – 151 °C (lit. m.p. 148 – 151 °C).[5]

Picolinamide (19)[25,26,27]

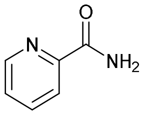

Using $Mg(NO_3)_2·6H_2O$ as catalyst. Following the general procedure described in section 4.6, 2-picolinic acid (370 mg, 3.0 mmol) was used as the acid species. The *title* compound was recovered after purification as a white solid (315 mg, 86%).

Using imidazole as catalyst. Following the general procedure described in section 5.6, 2-picolinic acid (370 mg, 3.0 mmol) was used as the acid species. The *title* compound was recovered after purification as a white solid (154 mg, 42%).

The spectroscopic data obtained using both methods was consistent with those reported.[26]

^{1}H NMR (500 MHz, DMSO-d_6) δ 8.63 (dd, *J* = 4.7, 1.6 Hz, 1H, H6), 8.04 (d, *J* = 7.8 Hz, 1H, H3), 7.98 (td, *J* = 7.6, 1.7 Hz, 1H, H4), 7.58 (ddd, *J* = 7.5, 4.8, 1.3 Hz, 1H, H5); ^{13}C NMR (126 MHz, DMSO-d_6) δ 166.0, 148.8, 148.4, 137.6, 126.4, 121.9; FT-IR (neat) υ in cm^{-1} = 1661 (C=O stretch).

Using $Mg(NO_3)_2·6H_2O$ as catalyst. HRMS-ESI calcd for $[C_6H_7N_2O]^+$: 123.0558 $[M+H]^+$, found 123.0562; m.p. 104 – 106 °C (lit. m.p. 105 – 106 °C).[27]

Using imidazole as catalyst. HRMS-ESI calcd for $[C_6H_7N_2O]^+$: 123.0558 $[M+H]^+$, found 123.0559; m.p. 103 – 105 °C (lit. m.p. 105 – 106 °C).[27]

Benzo[b]thiophene-2-carboxamide (20)[28,29]

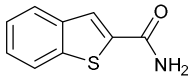

Using $Mg(NO_3)_2·6H_2O$ as catalyst. Following the general procedure described in section 4.6, thianaphthene-2-carboxylic acid (535 mg, 3.0 mmol) was used as the acid species. The *title* compound was recovered after purification by column chromatography (eluting EtOAc:Hexane 2:1) as a yellowish solid (440 mg, 83%).

Using imidazole as catalyst. Following the general procedure described in section 5.6, thianaphthene-2-carboxylic acid (535 mg, 3.0 mmol) was used as the acid species. The *title* compound was recovered after purification as a yellowish solid (420 mg, 79%).

The spectroscopic data obtained using both methods was consistent with those reported.[28]

^{1}H NMR (500 MHz, DMSO-d_6) δ 7.97 – 7.91 (m, 2H, Ar), 7.88 (s, 1H, CH=C), 7.44 – 7.38 (m, 2H, Ar); ^{13}C NMR (125 MHz, $CDCl_3$) δ 163.9, 140.7, 140.6, 139.9, 125.8, 124.6, 124.3, 123.8, 122.5; FT-IR (neat) υ in cm^{-1}: 1652 (C=O stretch), 1615 (C=C stretch).

Using $Mg(NO_3)_2·6H_2O$ as catalyst. HRMS-ESI calcd for $[C_9H_7NOSNa]^+$: 200.0146 $[M+Na]^+$, found = 200.0148; m.p. 173 – 175 °C (lit. m.p. 177 °C).[29]

Using imidazole as catalyst. HRMS-ESI calcd for $[C_9H_7NOSNa]^+$: 200.0146 $[M+Na]^+$, found = 200.0147; m.p. 173 – 175 °C (lit. m.p. 177 °C).[29]

2-Hydroxyacetamide (21)[30,31]

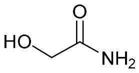

Using $Mg(NO_3)_2·6H_2O$ as catalyst. Following the general procedure described in section 4.6, hydroxyacetic acid (228 mg, 3.0 mmol) was used as the acid species. The *title* compound was recovered after purification by column chromatography (eluting DCM:MeOH 9:1) as an off-white solid (153 mg, 68%).

Using imidazole as catalyst. Following the general procedure described in section 4.6, hydroxyacetic acid (228 mg, 3.0 mmol) was used as the acid species. The *title* compound was recovered after purification as an off-white solid (137 mg, 61%).

The spectroscopic data obtained using both methods was consistent with those reported.[31]

^{1}H NMR (500 MHz, DMSO-d_6) δ 7.15 (br s, 2H, NH_2), 5.32 (br s, 1H, OH), 3.73 (br s, 2H, CH_2); ^{13}C NMR (126 MHz, DMSO-d_6) δ 174.5, 61.3; FT-IR (neat) υ in cm^{-1}: 1653 (C=O stretch).

Using $Mg(NO_3)_2·6H_2O$ as catalyst. HRMS-ESI calcd for $[C_2H_5NO_2Na]^+$: 98.0218 $[M+Na]^+$, found = 98.0221; m.p. 113 – 115 °C (lit. m.p. 114 – 115 °C).[30]

Using imidazole as catalyst. HRMS-ESI calcd for $[C_2H_5NO_2Na]^+$: 98.0218 $[M+Na]^+$, found = 98.0220; m.p. 114 – 116 °C (lit. m.p. 114 – 115 °C).[30]

9. Synthesis of Secondary Amides

N-Methyl phenylacetamide (22)[32]

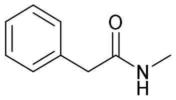

Using $Mg(NO_3)_2{\cdot}6H_2O$ as catalyst. Following the general procedure described in section 4.7, phenylacetic acid (400 mg, 3.0 mmol) was used as the acid species. The *title* compound was recovered after purification by column chromatography (EtOAc:DCM, 1:1) as a white solid (393 mg, 89%).

Using imidazole as catalyst. Following the general procedure described in section 5.8, phenylacetic acid (400 mg, 3.0 mmol) was used as the acid species. The *title* compound was recovered after purification as a white solid (370 mg, 84%).

The spectroscopic data obtained using both methods was consistent with those reported.[32]

^{1}H NMR (300 MHz, $CDCl_3$) δ 7.23 – 7.11 (m, 5H, Ar), 6.54 (br s, 1H, NH), 3.39 (s, 2H, CH_2), 2.58 (d, 3H, *J* = 3.0 Hz, CH_3); ^{13}C NMR (75 MHz, $CDCl_3$) δ 171.9, 135.1, 129.1, 128.6, 126.9, 43.1, 26.2; FT-IR (neat) υ in cm^{-1}: 1627 (C=O stretch).

Using $Mg(NO_3)_2{\cdot}6H_2O$ as catalyst. HRMS-ESI calcd for $[C_9H_{12}NO]^+$: 150.0919 $[M+H]^+$, found 150.0933; m.p. 50 – 52 °C (lit. m.p. 51 °C).[32]

Using imidazole as catalyst. HRMS-ESI calcd for $[C_9H_{12}NO]^+$: 150.0919 $[M+H]^+$, found 150.0922; m.p. 51 – 53 °C (lit. m.p. 51 °C).[32]

2-(4-Chlorophenyl)-*N*-methylacetamide (23)[33,34]

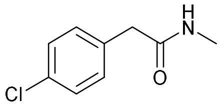

Using $Mg(NO_3)_2{\cdot}6H_2O$ as catalyst. Following the general procedure described in section 4.7, phenylacetic acid (400 mg, 3.0 mmol) was used as the acid species. The *title* compound was recovered after purification by column chromatography (EtOAc:DCM, 1:1) as a white solid (393 mg, 89%).

Using imidazole as catalyst. Following the general procedure described in section 5.8, phenylacetic acid (400 mg, 3.0 mmol) was used as the acid species. The *title* compound was recovered after purification as a white solid (370 mg, 84%).

The spectroscopic data obtained using both methods was consistent with those reported.[33]

^{1}H NMR (500 MHz, $CDCl_3$) δ 7.32 (d, *J* = 8.4 Hz, 2H, Ar), 7.19 (d, *J* = 8.4 Hz, 2H, Ar), 5.41 (s, 1H, NH), 3.53 (s, 2H, CH_2), 2.77 (d, *J* = 4.8 Hz, 3H, CH_3); ^{13}C NMR (126 MHz, $CDCl_3$) δ 171.1, 133.5, 133.5, 130.9, 129.3, 77.4, 77.2, 76.9, 43.1, 26.7; FT-IR (neat) υ in cm^{-1}: 1645 (C=O stretch).

Using $Mg(NO_3)_2{\cdot}6H_2O$ as catalyst. HRMS-ESI calcd for $[C_9H_{11}ClNO]^+$: 184.0529 $[M+H]^+$, found 184.0531; m.p. 105 – 106 °C (lit. m.p. 106 – 107 °C).[34]

Using imidazole as catalyst. HRMS-ESI calcd for $[C_9H_{11}ClNO]^+$: 184.0529 $[M+H]^+$, found 184.0530; m.p. 105 – 106 °C (lit. m.p. 106 – 107 °C).[34]

2-(4-Methoxyphenyl)-*N*-methylacetamide (24)[35,36]

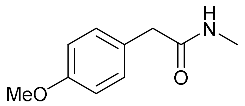

Using $Mg(NO_3)_2{\cdot}6H_2O$ as catalyst. Following the general procedure described in section 4.7, 4-methoxyphenylacetic acid (500 mg, 3 mmol) was used as the acid species. The *title* compound was recovered after purification as a yellow solid (496 mg, 92%).

Using imidazole as catalyst. Following the general procedure described in section 5.8, 4-methoxyphenylacetic acid (500 mg, 3 mmol) was used as the acid species. The *title* compound was recovered after purification as a yellow solid (512 mg, 95%).

The spectroscopic data obtained was consistent with those reported.[36]

^{1}H NMR (500 MHz, $CDCl_3$) δ 7.16 (d, *J* = 8.8 Hz, 2H, Ar), 6.89 (d, *J* = 8.8 Hz, 2H, Ar), 5.38 (br s, 1H, NH), 3.81 (s, 3H, OCH_3), 3.52 (s, 2H, CH_2), 2.74 (d, *J* = 4.9 Hz, 3H, CH_3); ^{13}C NMR (126 MHz, $CDCl_3$) δ 172.1, 158.8, 130.5, 126.8, 114.3, 55.2, 42.6, 26.4; FT-IR (neat) υ in cm^{-1}: 1653 (C=O stretch).

Using $Mg(NO_3)_2{\cdot}6H_2O$ as catalyst. HRMS-ESI calcd for $[C_{10}H_{14}NO_2]^+$: 180.1025 $[M+H]^+$, found 180.1028; m.p. 96 – 97 °C (lit. m.p. 96 – 97 °C).[35]

Using imidazole as catalyst. HRMS-ESI calcd for $[C_{10}H_{14}NO_2]^+$: 180.1025 $[M+H]^+$, found 180.1027; m.p. 96 – 97 °C (lit. m.p. 96 – 97 °C).[35]

***N*-Methyl-2,2-diphenylacetamide (25)**[37,38]

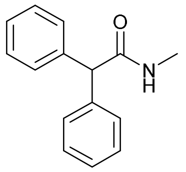

Using $Mg(NO_3)_2{\cdot}6H_2O$ as catalyst. Following the general procedure described in section 4.7, diphenylacetic acid (640 mg, 3.0 mmol) was used as the acid species. The *title* compound was recovered after purification as a white solid (652 mg, 96%).

Using imidazole as catalyst. Following the general procedure described in section 5.8, diphenylacetic acid (640 mg, 3.0 mmol) was used as the acid species. The *title* compound was recovered after purification as a white solid (604 mg, 89%).

^{1}H NMR (500 MHz, $CDCl_3$) δ 7.35 – 7.30 (m, 4H, Ar), 7.28 – 7.23 (m, 6H, Ar), 5.58 (s, 1H, NH), 4.93 (s, 1H, CH), 2.84 (d, *J* = 4.8 Hz, 3H, CH_3); ^{13}C NMR (126 MHz, $CDCl_3$) δ 172.6, 139.7, 129.1, 128.9, 127.4, 59.4, 26.8; FT-IR (neat) υ in cm^{-1}: 1641 (C=O stretch).

Using $Mg(NO_3)_2{\cdot}6H_2O$ as catalyst. HRMS-ESI calcd for $[C_{15}H_{16}NO]^+$: 226.1232 $[M+H]^+$, found 226.1233; m.p. 167 – 168 °C (lit. m.p. 167 – 168 °C).[38]

Using imidazole as catalyst. HRMS-ESI calcd for $[C_{15}H_{16}NO]^+$: 226.1232 $[M+H]^+$, found 226.1234; m.p. 167 – 168 °C (lit. m.p. 167 – 168 °C).[38]

***N*-Methyl-3-phenylpropanamide (26)**[39]

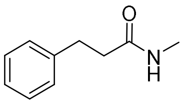

Using $Mg(NO_3)_2·6H_2O$ as catalyst. Following the general procedure described in section 4.7, hydrocinnamic acid (450 mg, 3.0 mmol) was used as the acid species. The *title* compound was recovered after purification as an off-white solid (293 mg, 60%).

Using imidazole as catalyst. Following the general procedure described in section 5.8, hydrocinnamic acid (450 mg, 3.0 mmol) was used as the acid species. The *title* compound was recovered after purification as an off-white solid (390 mg, 80%).

The spectroscopic data was consistent with those reported.[39]

^{1}H NMR (500 MHz, $CDCl_3$) δ 7.31 – 7.28 (m, 2H, Ar), 7.22 – 7.19 (m, 3H, Ar), 5.41 (br s, 1H, NH), 2.97 (t, *J* = 7.6 Hz, 2H, $PhCH_2$), 2.78 (d, *J* = 4.9 Hz, 3H, CH_3), 2.47 (t, *J* = 7.8 Hz, 2H, CH_2CO); ^{13}C NMR (125 MHz, $CDCl_3$) δ 172.2, 140.9, 128.4, 128.2, 126.1, 38.3, 31.7, 26.2; FT-IR (neat) υ in cm^{-1}: 1641 (C=O stretch).

Using $Mg(NO_3)_2·6H_2O$ as catalyst. HRMS-ESI calcd for $[C_{10}H_{13}NONa]^+$: 186.0895 $[M+Na]^+$, found 186.0897; m.p. 58 – 60 °C (lit. m.p. 59 – 60 °C).[39]

Using imidazole as catalyst. HRMS-ESI calcd for $[C_{10}H_{13}NONa]^+$: 186.0895 $[M+Na]^+$, found 186.0894; m.p. 58 – 60 °C (lit. m.p. 59 – 60 °C).[39]

3-(4-Methoxyphenyl)-*N*-methylpropanamide (27)[40, 41]

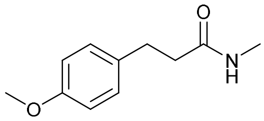

Using $Mg(NO_3)_2·6H_2O$ as catalyst. Following the general procedure described in section 4.7, 3-(4-methoxyphenyl)propionic acid (540 mg, 3.0 mmol) was used as the acid species. The *title* compound was recovered after purification as a white solid (445 mg, 77%).

Using imidazole as catalyst. Following the general procedure described in section 5.8, 3-(4-methoxyphenyl)propionic acid (540 mg, 3.0 mmol) was used as the acid species. The *title* compound was recovered after purification as a white solid (538 mg, 93%).

The spectroscopic data obtained was consistent with those reported.[41]

^{1}H NMR (500 MHz, $CDCl_3$) δ 7.12 (d, *J* = 8.8 Hz, 2H, Ar), 6.83 (d, *J* = 8.8 Hz, 2H, Ar), 5.38 (br s, 1H, NH), 3.79 (s, 3H, OCH_3), 2.91 (t, *J* = 7.6 Hz, 2H, $PhCH_2$), 2.77 (d, *J* = 4.9 Hz, 3H, CH_3), 2.44 (t, *J* = 7.8 Hz, 2H, CH_2CO); ^{13}C NMR (125 MHz, $CDCl_3$) δ 172.9, 158.0, 132.9, 129.2, 113.8, 55.2, 38.6, 30.8, 26.2; FT-IR (neat) υ in cm^{-1}: 1639 (C=O stretch).

Using $Mg(NO_3)_2·6H_2O$ as catalyst. HRMS-ESI calcd for $[C_{11}H_{15}NONa]^+$: 216.1000 $[M+Na]^+$, found 216.1003; m.p. 85 – 87 °C (lit. m.p. 87.5 – 88 °C).[41]

Using imidazole as catalyst. HRMS-ESI calcd for $[C_{11}H_{15}NONa]^+$: 216.1000 $[M+Na]^+$, found 216.1002; m.p. 86 – 87 °C (lit. m.p. 87.5 – 88 °C).[41]

***N*-Methylbenzamide (28)**[42]

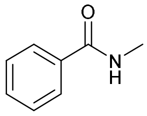

Using $Mg(NO_3)_2·6H_2O$ as catalyst. Following the general procedure described in section 4.7, benzoic acid (366 mg, 3.0 mmol) did not give the corresponding amide **28**.

Using imidazole as catalyst. Following the general procedure described in section 5.8, benzoic acid (366 mg, 3.0 mmol) gave the corresponding amide **28** in 25% conversion. Conversion was determined by analysis of the ^{1}H NMR spectrum by comparison of the peaks at 7.85 (d, 2H, CH, starting material) and 7.67 (d, 2H, CH, **28**).

***N*-Methyl hexanamide (29)**[43]

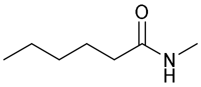

Using $Mg(NO_3)_2·6H_2O$ as catalyst. Following the general procedure described in section 4.7, hexanoic acid (380 µL, 3.0 mmol) was used as the acid species. The *title* compound was recovered after purification by column chromatography (eluting with EtOAc:DCM, 1:1) as a colourless liquid (349 mg, 89%).

Using imidazole as catalyst. Following the general procedure described in section 5.8, hexanoic acid (380 µL, 3.0 mmol) was used as the acid species. The *title* compound was recovered after purification as a colourless liquid (274 mg, 70%).

The spectroscopic data obtained using both methods was consistent with those reported.[43]

^{1}H NMR (300 MHz, $CDCl_3$) δ 7.07 (br s, 1H, NH), 2.59 (d, 3H, *J* = 4.7 Hz, NCH_3), 2.01 (t, 2H, *J* = 7.6 Hz, CH_2CO), 1.49 – 1.39 (quin, *J* = 7.6 Hz, 2H, $\underline{CH_2}$-CH_2-CO), 1.16 – 1.05 (m, 4H, CH_3-$\underline{CH_2}$-$\underline{CH_2}$), 0.69 (t, 3H, *J* = 7.2 Hz, $\underline{CH_3}$-CH_2); ^{13}C NMR (75 MHz, $CDCl_3$) δ 174.6, 36.5, 31.5, 26.2, 25.2, 22.5, 14.0; FT-IR (neat) υ in cm^{-1}: 1646 (C=O stretch).

Using $Mg(NO_3)_2·6H_2O$ as catalyst. HRMS-ESI calcd for $[C_7H_{16}NO]^+$: 130.1232 $[M+H]^+$, found 130.1234.

Using imidazole as catalyst. HRMS calcd for $[C_7H_{15}NONa]^+$: 152.1051 $[M+Na]^+$, found 152.1054.

***N*-Methyltrimethylacetamide (30)**[44,45]

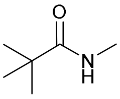

Using $Mg(NO_3)_2·6H_2O$ as catalyst. Following the general procedure described in section 4.7, trimethylacetic acid (310 mg, 3.0 mmol) was used as the acid species. The *title* compound was recovered after purification as a white solid (192 mg, 55%).

Using imidazole as catalyst. Following the general procedure described in section 5.8, trimethylacetic acid (310 mg, 3.0 mmol) was used as the acid species. The *title* compound was recovered after purification as a colourless liquid (133 mg, 38%).

The spectroscopic data obtained using both methods was consistent with those reported.[44,45]

^{1}H NMR (400 MHz, $CDCl_3$) δ 5.83 (br s, 1H, NH), 2.77 (d, *J* = 4.8 Hz, 3H, NCH_3), 1.17 (s, 9H, CH_3); ^{13}C NMR (100 MHz, $CDCl_3$) δ 179.5, 38.8, 27.6, 26.6; FT-IR (neat) υ in cm^{-1}: 1642 (C=O stretch).

Using $Mg(NO_3)_2{\cdot}6H_2O$ as catalyst. HRMS-ESI calcd for $[C_6H_{14}NO]^+$: 116.1075 [M+H]$^+$, found 116.1077; m.p. 48 – 50 °C (lit. m.p. 49 – 50 °C).[44]

Using imidazole as catalyst. HRMS-ESI calcd for $[C_6H_{14}NO]^+$: 116.1075 [M+H]$^+$, found 116.1076; m.p. 48 – 50 °C (lit. m.p. 49 – 50 °C).[44]

***N*-Methyl Oleamide (31)**[46,47]

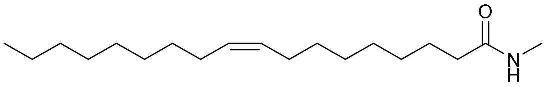

Using $Mg(NO_3)_2{\cdot}6H_2O$ as catalyst. Following the general procedure described in section 4.7, oleic acid (950 μL, 1.0 mmol) was used as the acid species. The *title* compound was recovered after purification by column chromatography (EtOAc:Pentane, 1:2) as an off-white solid (688 mg, 78%)

Using imidazole as catalyst. Following the general procedure described in section 5.8, trimethylacetic acid (950 μL, 3.0 mmol) was used as the acid species. The *title* compound was recovered after purification as an off-white solid (529 mg, 60%).

The spectroscopic data obtained using both methods was consistent with those reported.[46,47]

^{1}H NMR (400 MHz, $CDCl_3$) δ 5.54 (br s, 1H, NH), 5.38 – 5.28 (m, 2H, CH=), 2.79 (d, *J* = 4.8 Hz, 3H, CH_3NH), 2.15 (t, *J* = 7.5 Hz, 2H, CH_2), 2.00 – 1.97 (m, 4H, CH_2), 1.65 – 1.57 (m, 2H, CH_2), 1.35 – 1.21 (m, 20H, CH_2), 0.87 (t, *J* = 6.7 Hz, 3H, CH_3); ^{13}C NMR (100 MHz, $CDCl_3$) δ 174.0, 130.1, 129.9, 36.9, 32.0, 29.9, 29.8, 29.6, 29.5, 29.4, 29.4, 29.3, 27.4, 27.4, 27.3, 26.4, 25.9, 22.8, 14.2; FT-IR (neat) υ in cm^{-1}: 1638 (C=O stretch), 1467 (C=C stretch).

Using $Mg(NO_3)_2{\cdot}6H_2O$ as catalyst. HRMS-ESI calcd for $[C_{19}H_{38}NO]^+$: 296.2953 [M+H]$^+$, found 296.2955; m.p. 36 – 37 °C (lit. m.p. 34 – 35 °C).[47]

Using imidazole as catalyst. HRMS-ESI calcd for $[C_{19}H_{38}NO]^+$: 296.2953 [M+H]$^+$, found 296.2954; m.p. 36 – 37 °C (lit. m.p. 34 – 35 °C).[47]

***N*-Methylpicolinamide (32)**[48,49]

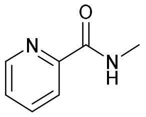

Using $Mg(NO_3)_2{\cdot}6H_2O$ as catalyst. Following the general procedure described in section 4.7, 2-picolinic acid (370 mg, 3 mmol) was used as the acid species. The *title* compound was recovered after purification by column chromatography as a clear oil (340 mg, 83%).

Using imidazole as catalyst. Following the general procedure described in section 5.8, 2-picolinic acid (370 mg, 3 mmol) was used as the acid species. The *title* compound was recovered after purification as a clear oil (310 mg, 76%).

The spectroscopic data obtained using both methods was consistent with those reported.[48,49]

^{1}H NMR (500 MHz, $CDCl_3$) δ 8.53 (d, *J* = 4.7 Hz, 1H, H6), 8.19 (dd, *J* = 7.8, 1.1 Hz, 1H, H3), 8.03 (s, 1H, NH), 7.84 (td, *J* = 7.7, 1.7 Hz, 1H, H4), 7.41 (ddd, *J* = 7.6, 4.8, 1.2 Hz, 1H, H5), 3.03 (d, *J* = 5.1 Hz, 3H,

CH_3); ^{13}C NMR (126 MHz, $CDCl_3$) δ 165.1, 150.1, 148.2, 137.5, 126.2, 122.2, 77.2, 26.2; FT-IR (neat) υ in cm^{-1}: 1645 (C=O stretch).

Using $Mg(NO_3)_2{\cdot}6H_2O$ as catalyst. HRMS-ESI calcd for $[C_7H_9N_2O]^+$: 137.0715 [M+H]$^+$, found 137.0717.

Using imidazole as catalyst. HRMS-ESI calcd for $[C_7H_9N_2O]^+$: 137.0715 [M+H]$^+$, found 137.0720.

***N*,2-Diphenylacetamide (33)**[1,50]

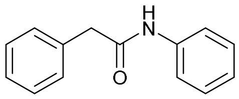

Using $Mg(NO_3)_2{\cdot}6H_2O$ as catalyst. Following the general procedure described in section 4.7, phenylacetic acid (410 mg, 3.0 mmol) was used as the acid species. The *title* compound was recovered after purification as a yellow solid (426 mg, 67%).

Using imidazole as catalyst. Following the general procedure described in section 5.8, phenylacetic acid (410 mg, 3.0 mmol) was used as the acid species. The *title* compound was recovered after purification as a yellow solid (457 mg, 72%).

The spectroscopic data obtained using both methods was consistent with those reported.[1,50]

^1H NMR (500 MHz, $CDCl_3$) δ 7.45 – 7.36 (m, 4H, Ar), 7.36 – 7.31 (m, 3H, Ar), 7.27 (t, *J* = 7.1 Hz, 2H, Ar), 7.08 (t, *J* = 7.1 Hz, 1H, Ar), 3.72 (s, 2H, CH_2); ^{13}C NMR (126 MHz, $CDCl_3$) δ 169.3, 137.8, 134.6, 129.6, 129.3, 129.0, 127.7, 124.6, 120.0, 44.9; FT-IR (neat) υ in cm^{-1}: 1648 (C=O stretch).

Using $Mg(NO_3)_2{\cdot}6H_2O$ as catalyst. HRMS-ESI calcd for $[C_{14}H_{14}NO]^+$: 212.1075 [M+H]$^+$, found 212.1080. m.p. 117 – 118 °C (lit. m.p. 119 – 120 °C).[50]

Using imidazole as catalyst. HRMS-ESI calcd for $[C_{14}H_{14}NO]^+$: 212.1075 [M+H]$^+$, found 212.1079. m.p. 117 – 118 °C (lit. m.p. 119 – 120 °C).[50]

***N*,3-Diphenylpropanamide (34)**[51,52]

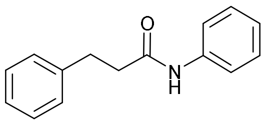

Using $Mg(NO_3)_2{\cdot}6H_2O$ as catalyst. Following the general procedure described in section 4.7, hydrocinnamic acid (450 mg, 3.0 mmol) was used as the acid species. The *title* compound was recovered after purification as a yellow solid (438 mg, 65%).

Using imidazole as catalyst. Following the general procedure described in section 5.8, hydrocinnamic acid (450 mg, 3.0 mmol) was used as the acid species. The *title* compound was recovered after purification as a yellow solid (459 mg, 68%).

The spectroscopic data obtained using both methods was consistent with those reported.[51,52]

^1H NMR (400 MHz, $CDCl_3$) δ 7.43 (d, *J* = 7.9 Hz, 2H, Ar), 7.32 – 7.29 (m, 4H, Ar), 7.25 – 7.21 (m, 3H, Ar), 7.16 (br s, 1H, NH), 7.09 (t, *J* = 7.4 Hz, 1H, Ar), 3.05 (t, *J* = 7.6 Hz, 2H, $PhCH_2$), 2.66 (t, *J* = 7.6 Hz, 2H, CH_2CO); ^{13}C NMR (100 MHz, $CDCl_3$) δ 170.5, 140.8, 137.9, 129.1, 128.8, 128.5, 126.5, 124.4, 120.1, 39.6, 31.7; FT-IR (neat) υ in cm^{-1}: 1652 (C=O stretch).

Using $Mg(NO_3)_2{\cdot}6H_2O$ as catalyst. HRMS-ESI calcd for $[C_{15}H_{16}NO]^+$: 226.1232 $[M+H]^+$, found 226.1237. m.p. 88 – 90 °C (lit. m.p. 88 – 90 °C).[52]

Using imidazole as catalyst. HRMS-ESI calcd for $[C_{15}H_{16}NO]^+$: 226.1232 $[M+H]^+$, found 226.1237. m.p. 88 – 90 °C (lit. m.p. 88 – 90 °C).[52]

***N*-Phenylhexanamide (35)**[53,54]

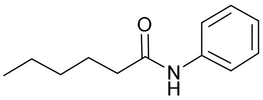

Using $Mg(NO_3)_2{\cdot}6H_2O$ as catalyst. Following the general procedure described in section 4.7, hexanoic acid (380 µL, 3.0 mmol) was used as the acid species. The *title* compound was recovered after purification as a yellow solid (429 mg, 74%).

Using imidazole as catalyst. Following the general procedure described in section 5.8, hexanoic acid (380 µL, 3.0 mmol) was used as the acid species. The *title* compound was recovered after purification as a yellow solid (365 mg, 63%).

The spectroscopic data obtained using both methods was consistent with those reported.[53,54]

^{1}H NMR (500 MHz, $CDCl_3$) δ 7.66 (br s, 1H, NH), 7.5 (d, *J* = 7.6 Hz, 2H, Ar), 7.29 (t, *J* = 7.9 Hz, 2H, Ar), 7.08 (t, *J* = 7.4 Hz, 1H, Ar), 2.34 (t, *J* = 7.5 Hz, 2H, CH_2CO), 1.71 (quint, *J* = 7.5, 2H, CH_2), 1.33 (m, 4H, CH_2), 0.90 (t, *J* = 7.0 Hz, 3H, CH_3); ^{13}C NMR (126 MHz, $CDCl_3$) δ 171.8, 138.1, 128.9, 124.1, 119.9, 37.7, 31.4, 25.4, 22.4, 13.9; FT-IR (neat) υ in cm^{-1}: 1663 (C=O stretch).

Using $Mg(NO_3)_2{\cdot}6H_2O$ as catalyst. HRMS-ESI calcd for $[C_{12}H_{18}NO]^+$: 192.1388 $[M+H]^+$, found 192.1392. m.p. 96 – 98 °C (lit. m.p. 97 °C).[54]

Using imidazole as catalyst. **Using $Mg(NO_3)_2{\cdot}6H_2O$ as catalyst**. HRMS-ESI calcd for $[C_{12}H_{18}NO]^+$: 192.1388 $[M+H]^+$, found 192.1390. m.p. 96 – 98 °C (lit. m.p. 97 °C).[54]

10. ^{1}H and ^{13}C NMR.

Identical spectroscopic data was obtained using both methodologies.

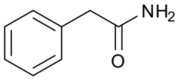

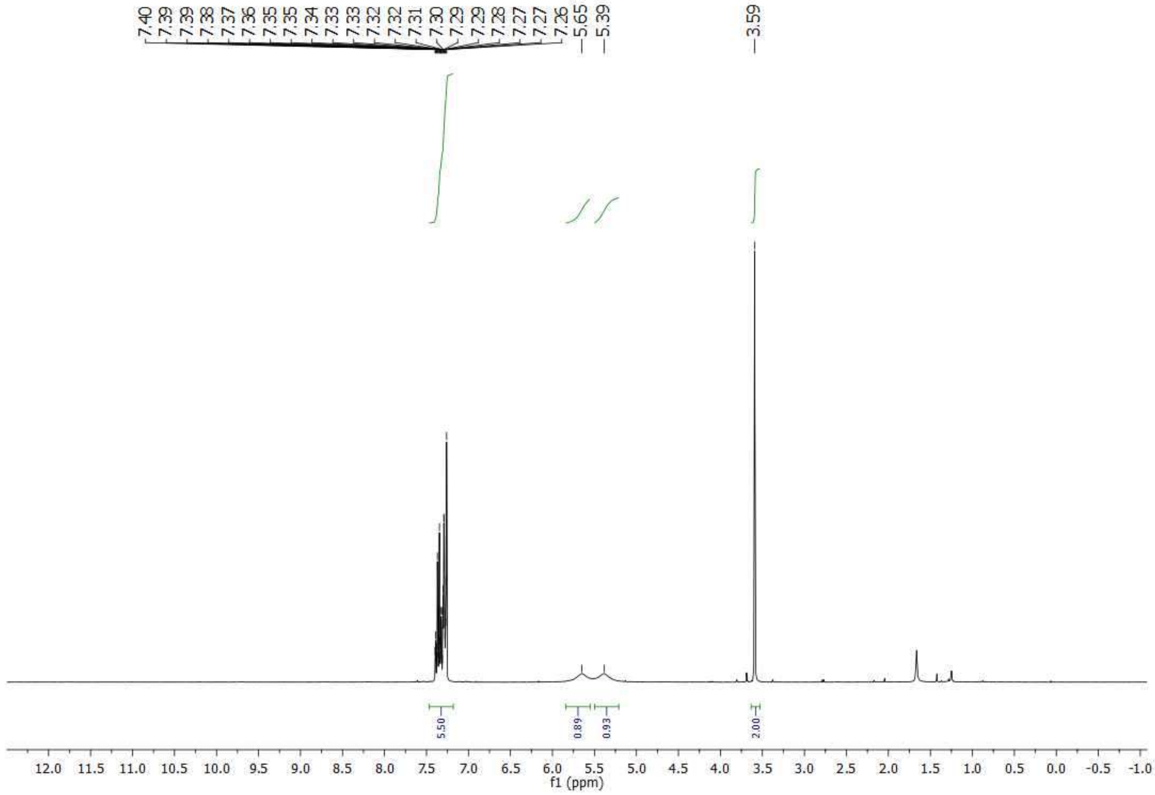

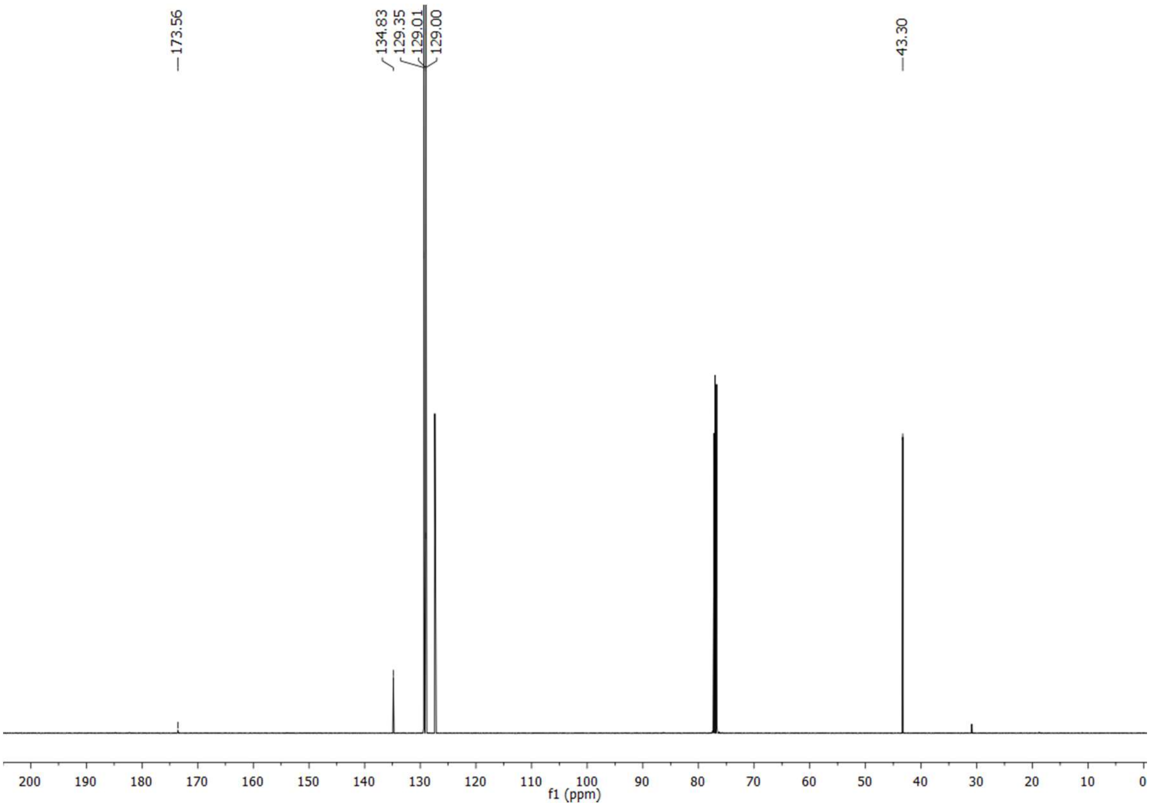

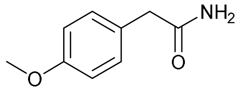
NH2
O
O

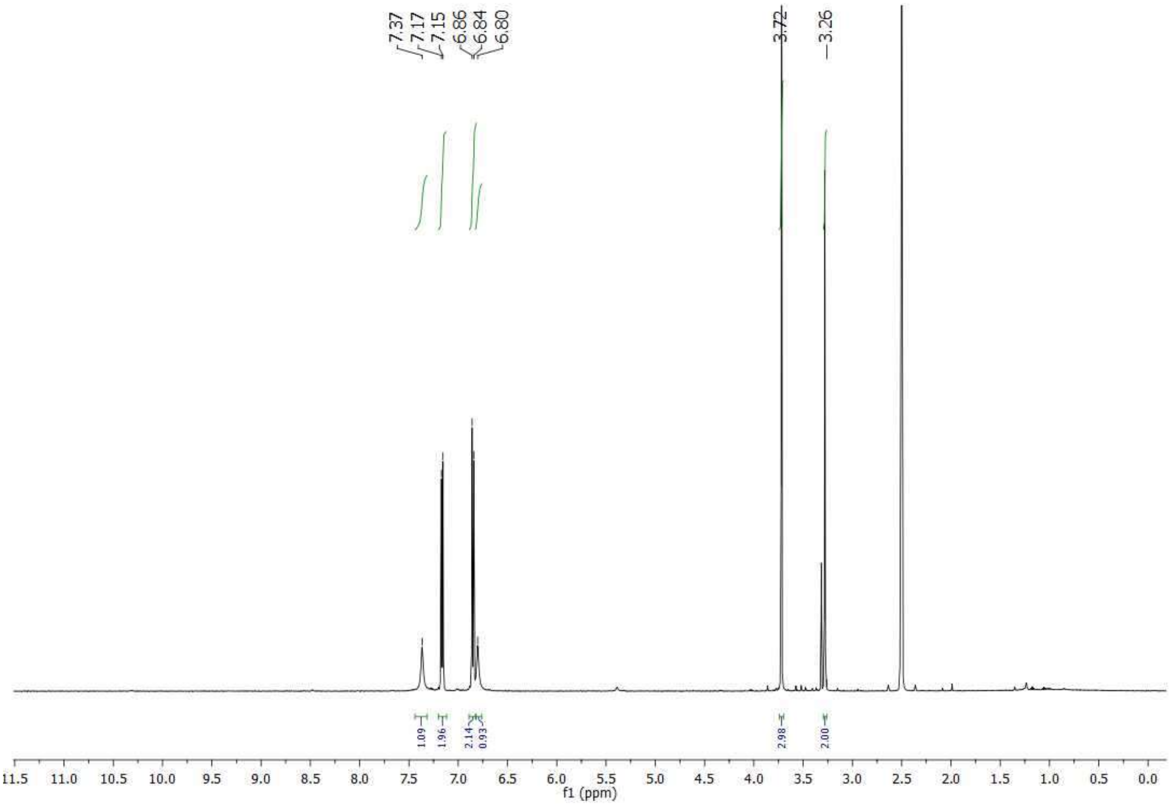
7.37
7.17
7.15
6.86
6.84
6.80
3.72
3.26
1.09
1.96
2.14
0.93
2.98
2.00
11.5 11.0 10.5 10.0 9.5 9.0 8.5 8.0 7.5 7.0 6.5 6.0 5.5 5.0 4.5 4.0 3.5 3.0 2.5 2.0 1.5 1.0 0.5 0.0
f1 (ppm)

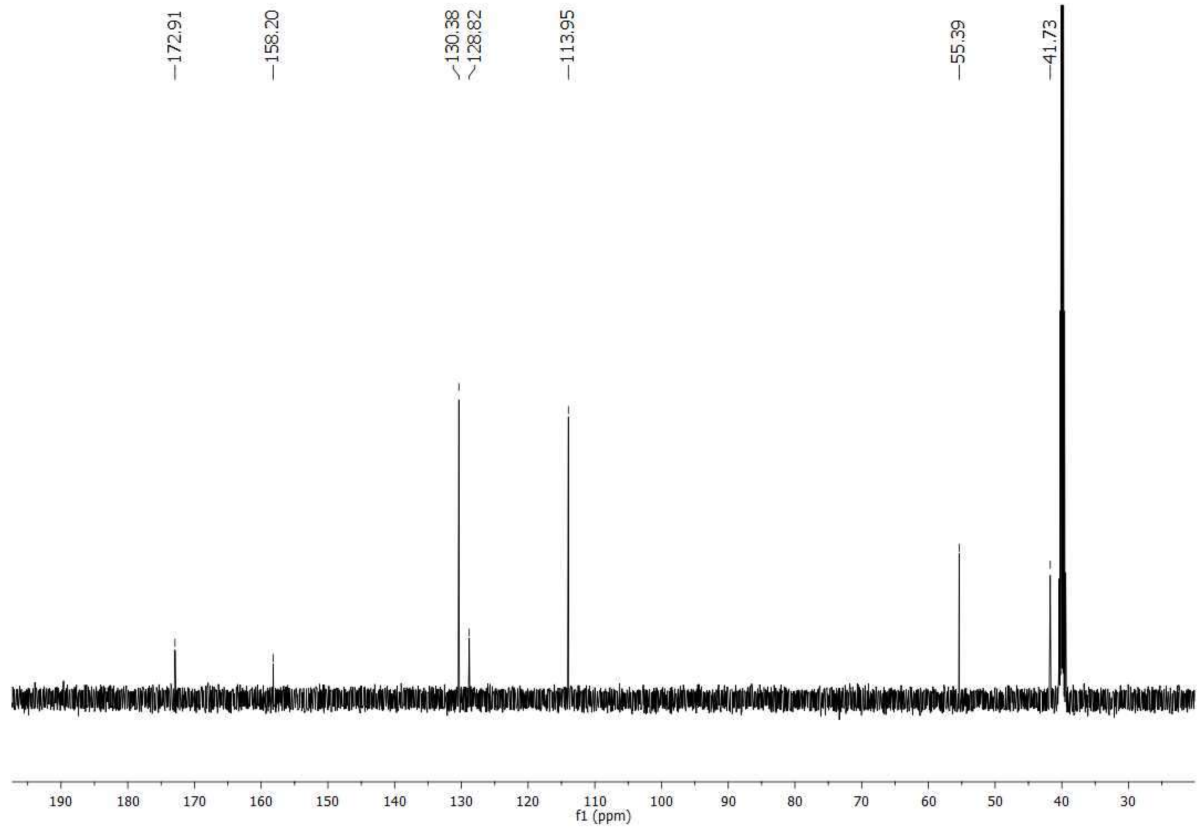
172.91
158.20
130.38
128.82
113.95
55.39
41.73
190 180 170 160 150 140 130 120 110 100 90 80 70 60 50 40 30
f1 (ppm)

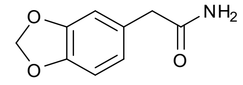
NH2
O
O
O

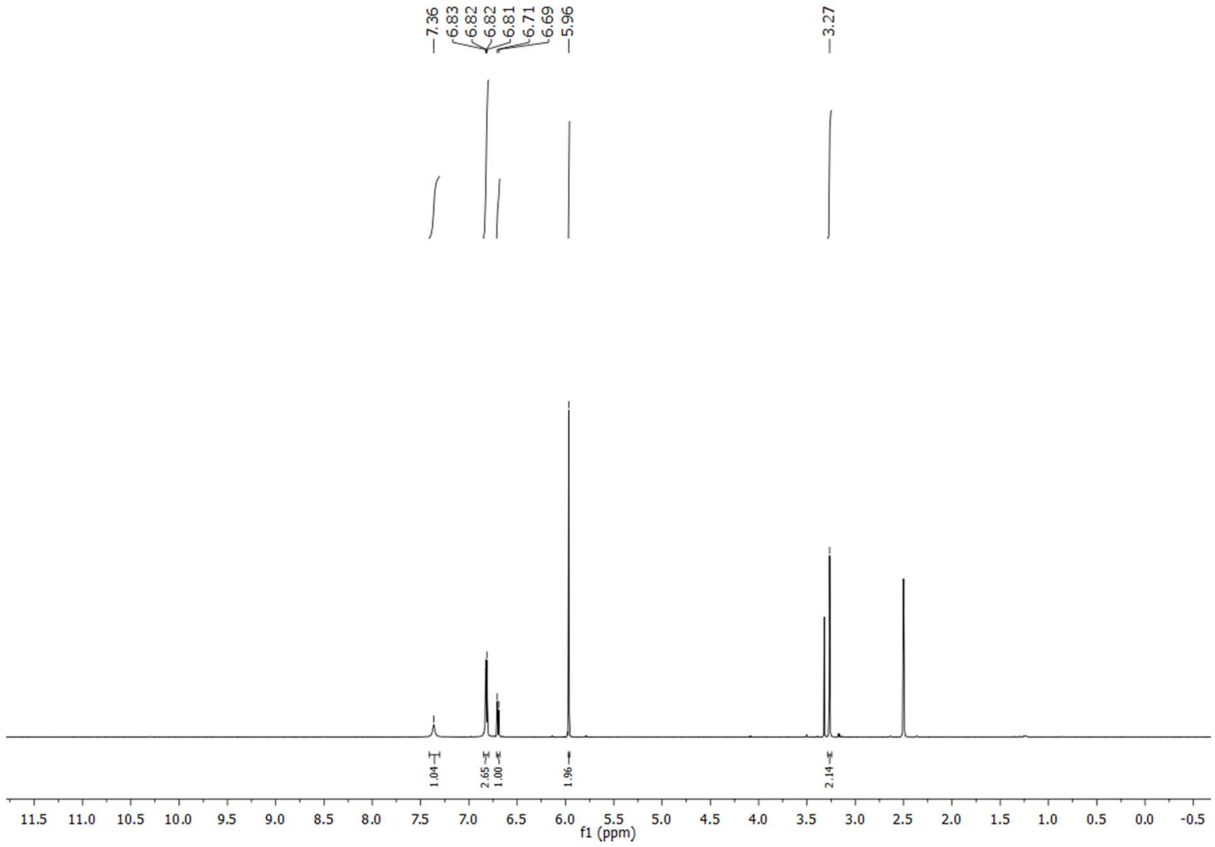
7.36
6.83
6.82
6.82
6.81
6.71
6.69
5.96
3.27
1.04
2.65
1.00
1.96
2.14
f1 (ppm)

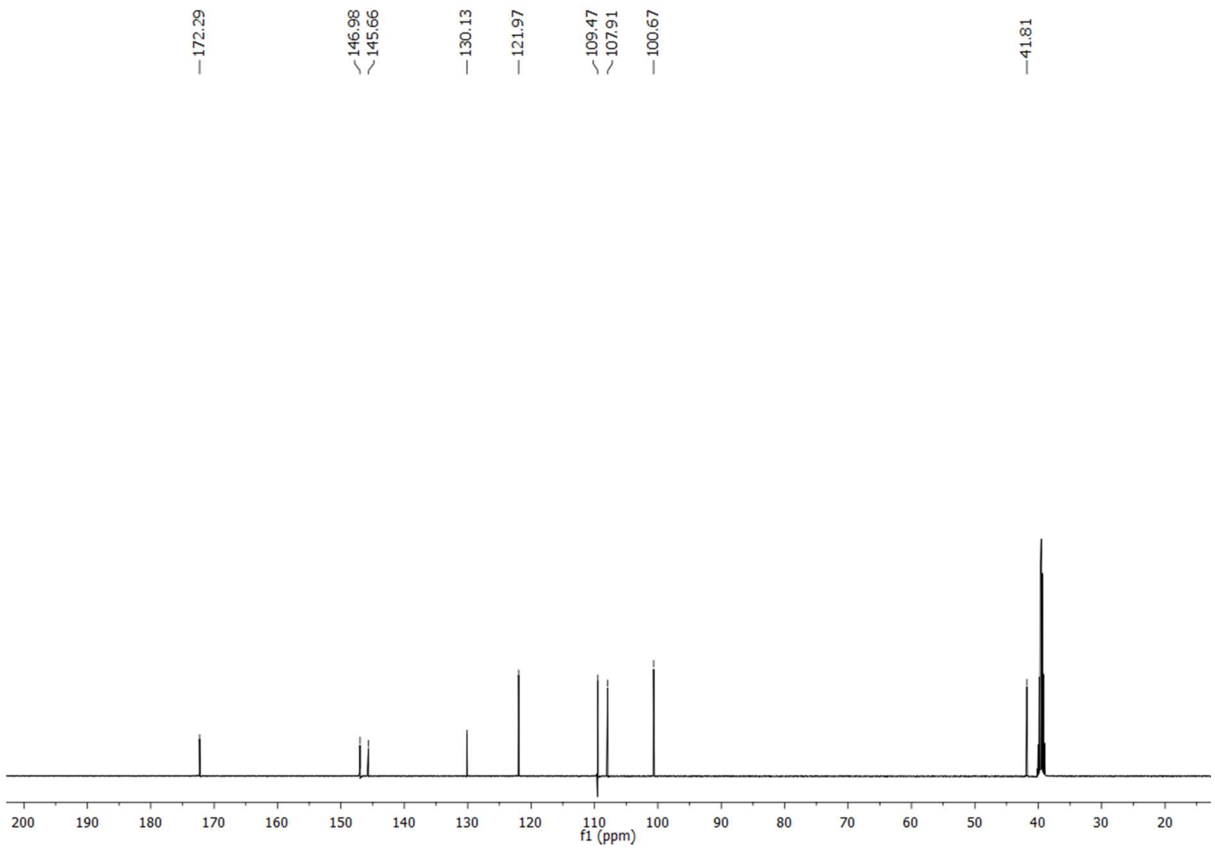
172.29
146.98
145.66
130.13
121.97
109.47
107.91
100.67
41.81
f1 (ppm)

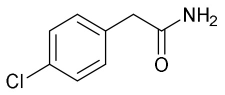
NH2
O
Cl

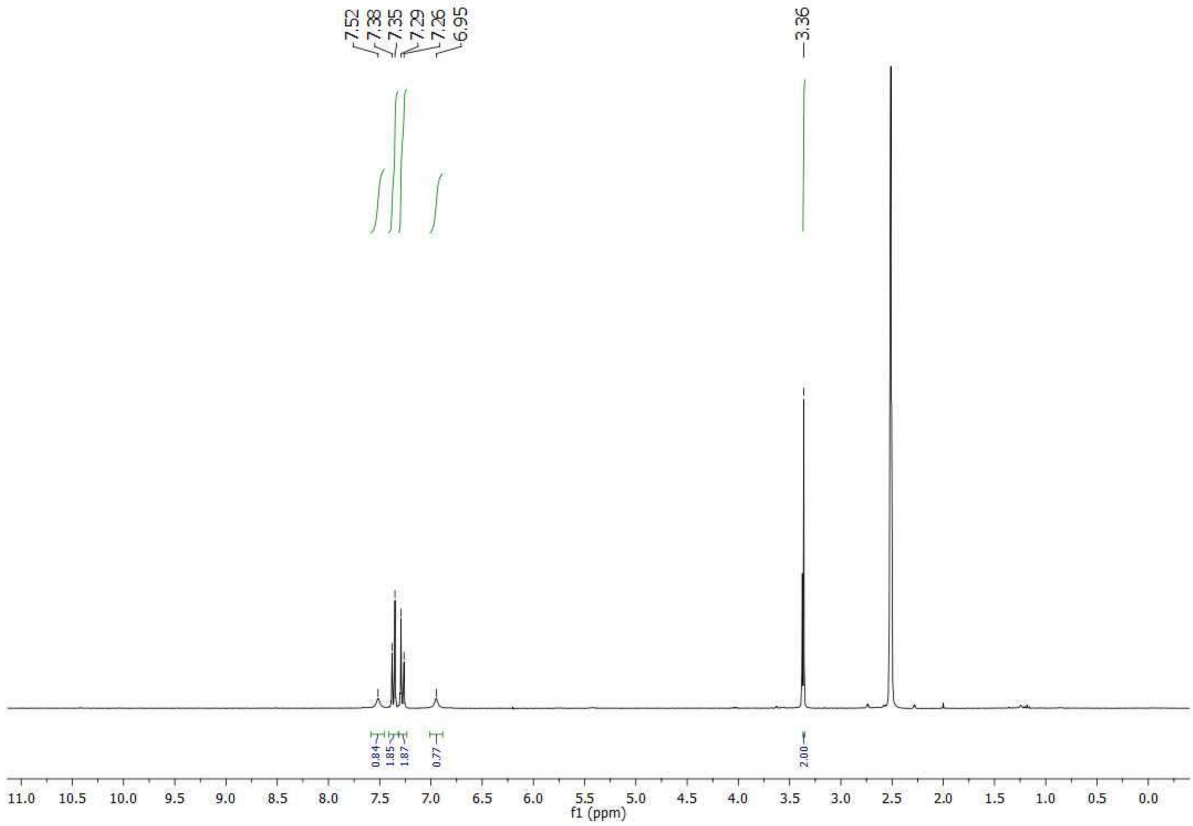
7.52
7.38
7.35
7.29
7.26
6.95
3.36
0.84
1.85
1.87
0.77
2.00
11.0 10.5 10.0 9.5 9.0 8.5 8.0 7.5 7.0 6.5 6.0 5.5 5.0 4.5 4.0 3.5 3.0 2.5 2.0 1.5 1.0 0.5 0.0
f1 (ppm)

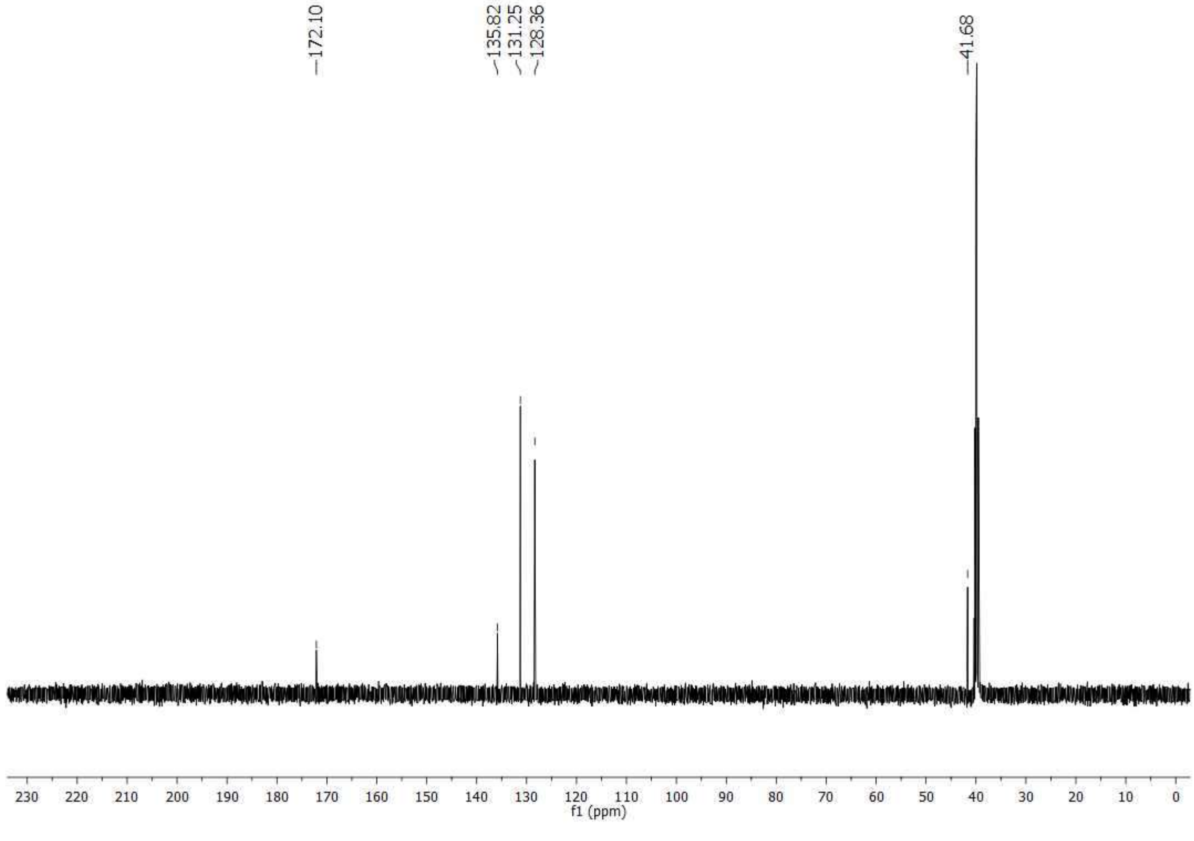
172.10
135.82
131.25
128.36
41.68
230 220 210 200 190 180 170 160 150 140 130 120 110 100 90 80 70 60 50 40 30 20 10 0
f1 (ppm)

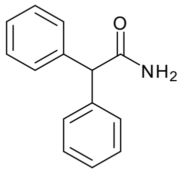
O
NH2

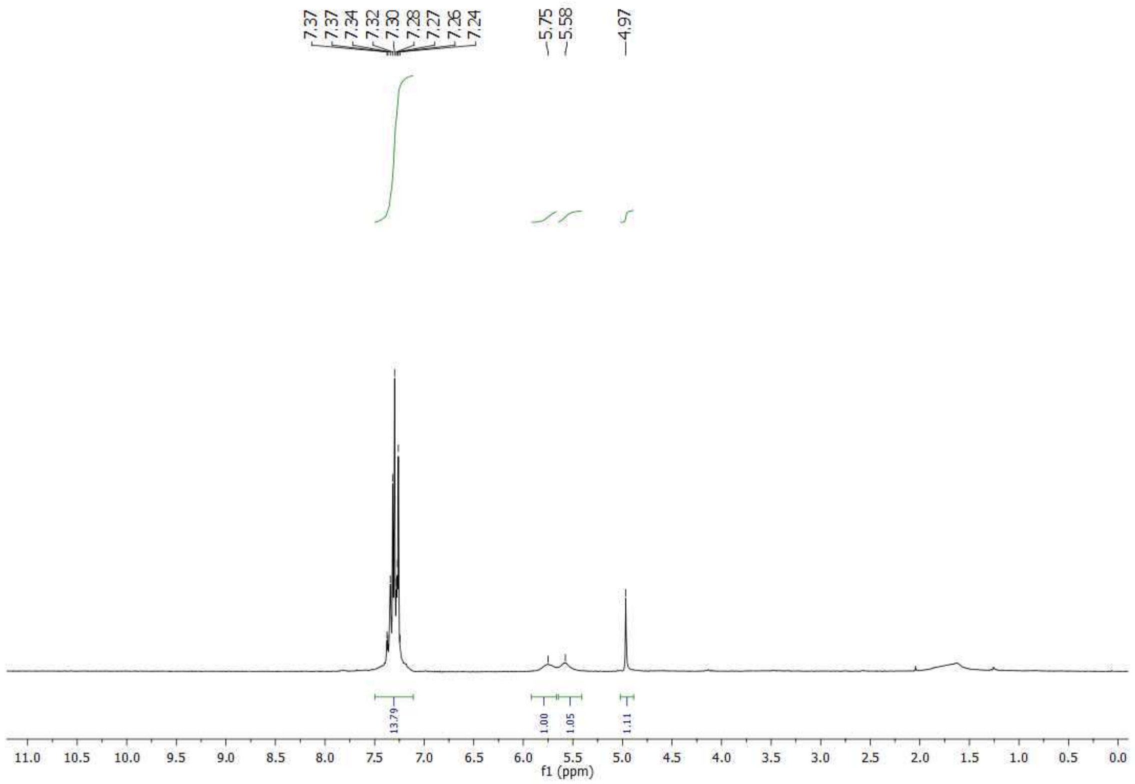
7.37
7.37
7.34
7.32
7.30
7.28
7.27
7.26
7.24
5.75
5.58
4.97
13.79
1.00
1.05
1.11
f1 (ppm)

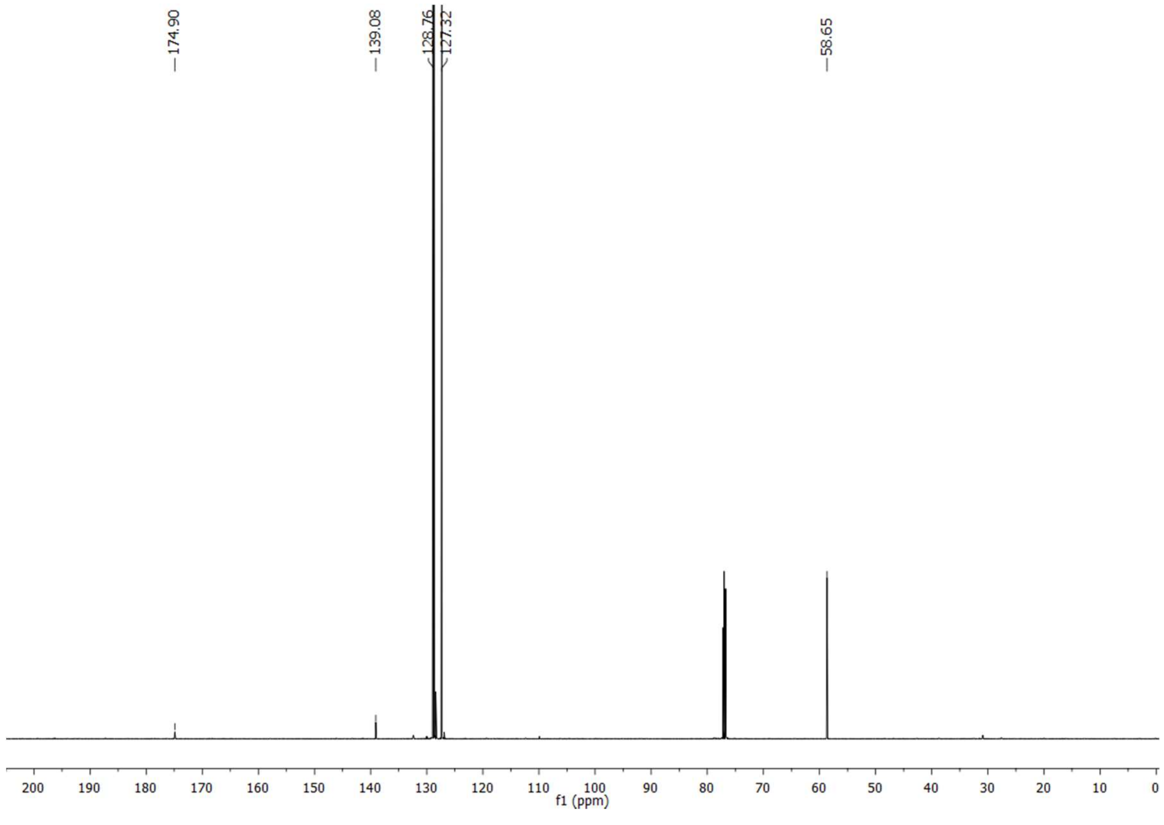
174.90
139.08
128.76
127.32
58.65
f1 (ppm)

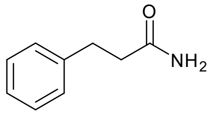
O
NH2

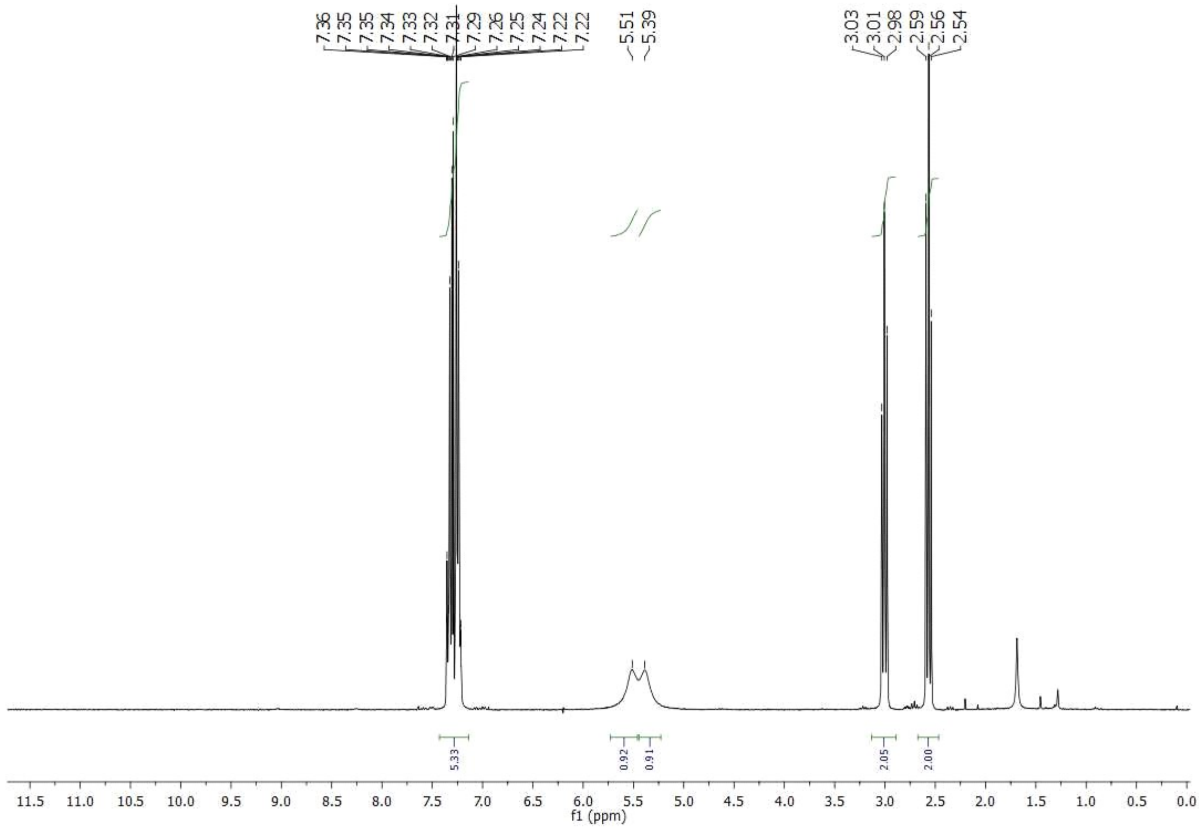
7.36
7.35
7.35
7.34
7.33
7.32
7.31
7.29
7.26
7.25
7.24
7.22
7.22
5.51
5.39
3.03
3.01
2.98
2.59
2.56
2.54
5.33
0.92
0.91
2.05
2.00
11.5 11.0 10.5 10.0 9.5 9.0 8.5 8.0 7.5 7.0 6.5 6.0 5.5 5.0 4.5 4.0 3.5 3.0 2.5 2.0 1.5 1.0 0.5 0.0
f1 (ppm)

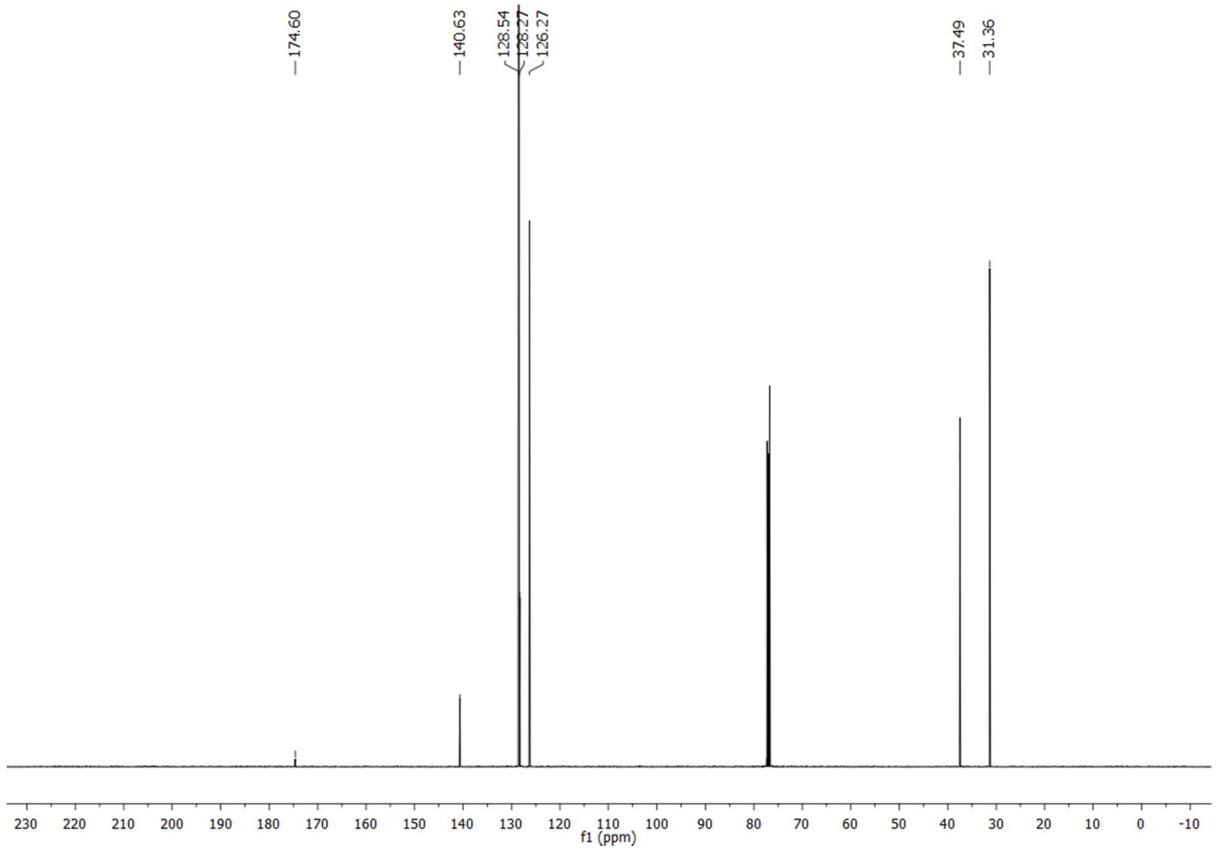
174.60
140.63
128.54
128.27
126.27
37.49
31.36
230 220 210 200 190 180 170 160 150 140 130 120 110 100 90 80 70 60 50 40 30 20 10 0 -10
f1 (ppm)

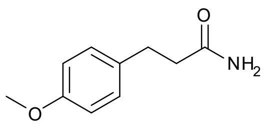
O
NH2
O

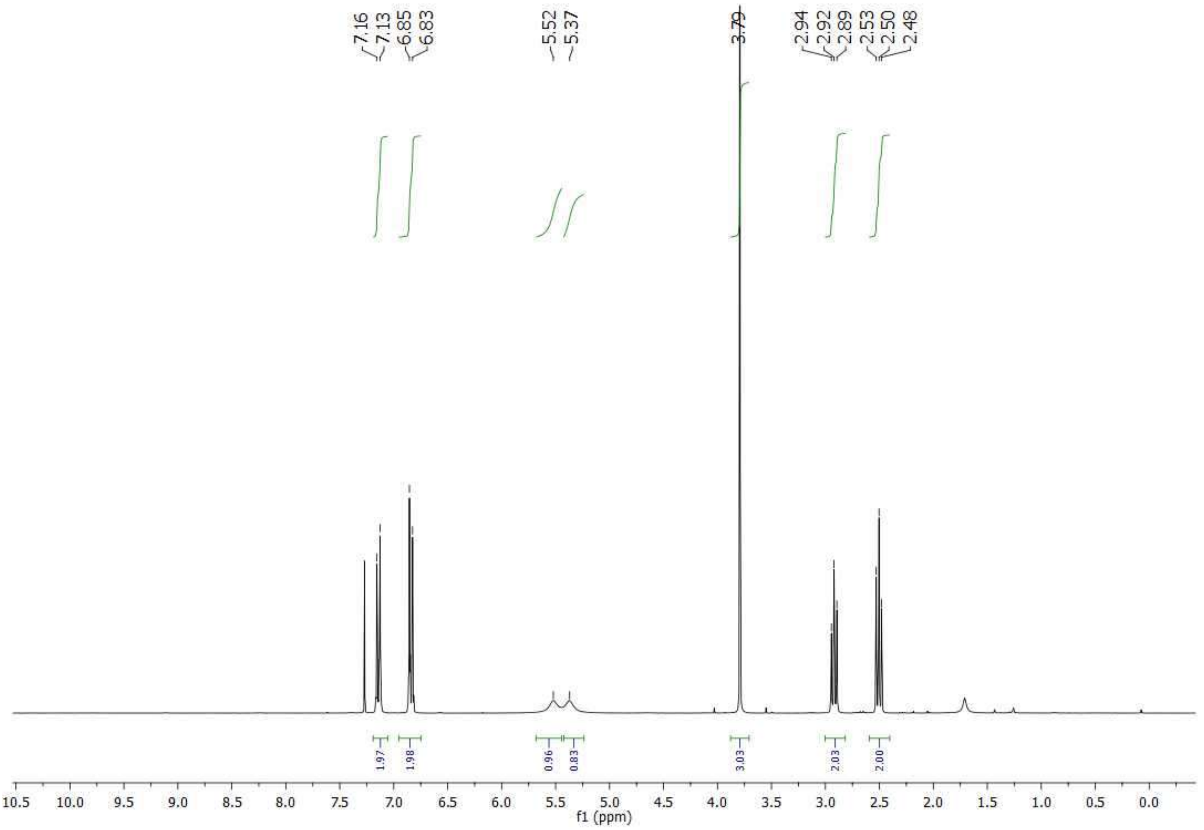
7.16
7.13
6.85
6.83
5.52
5.37
3.79
2.94
2.92
2.89
2.53
2.50
2.48
1.97
1.98
0.96
0.83
3.03
2.03
2.00
10.5 10.0 9.5 9.0 8.5 8.0 7.5 7.0 6.5 6.0 5.5 5.0 4.5 4.0 3.5 3.0 2.5 2.0 1.5 1.0 0.5 0.0
f1 (ppm)

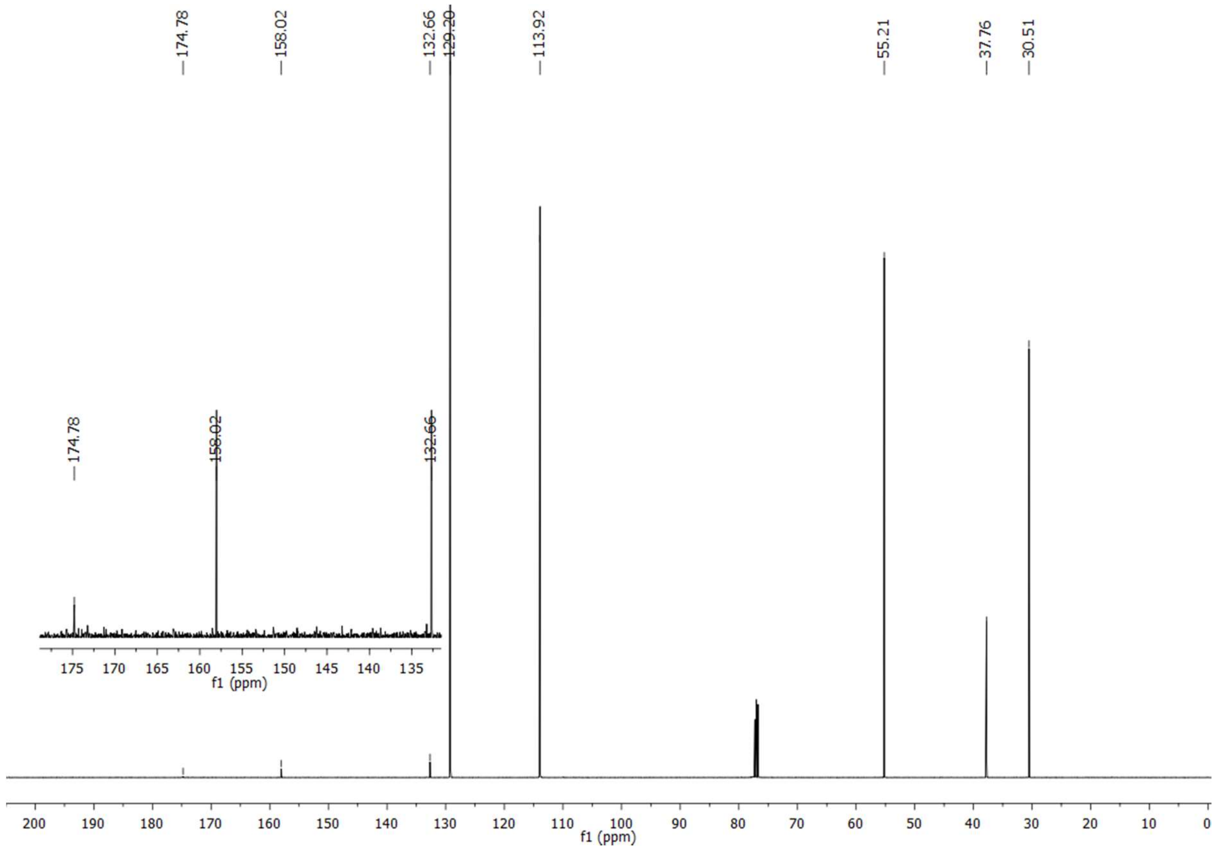
174.78
158.02
132.66
129.20
113.92
55.21
37.76
30.51
174.78
158.02
132.66
175 170 165 160 155 150 145 140 135
f1 (ppm)
200 190 180 170 160 150 140 130 120 110 100 90 80 70 60 50 40 30 20 10 0
f1 (ppm)

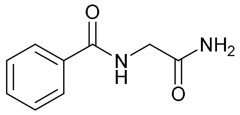
O
NH2
N
H
O

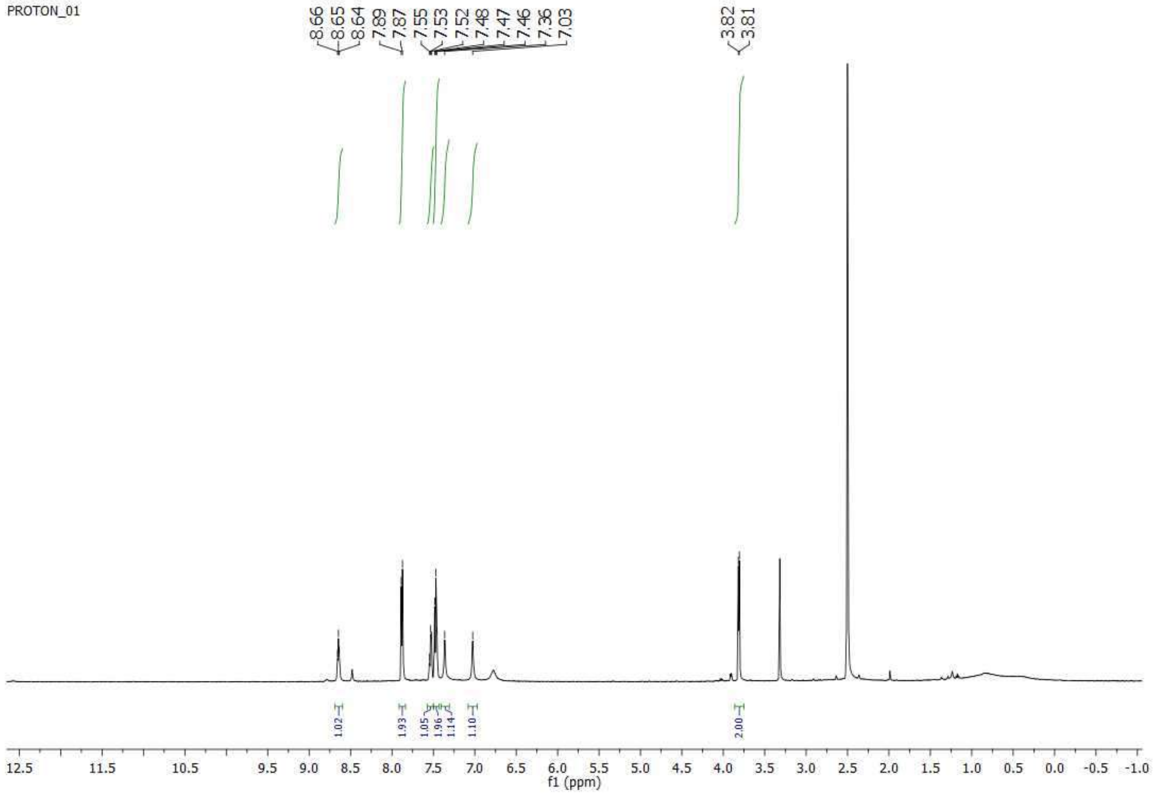
PROTON_01
8.66
8.65
8.64
7.89
7.87
7.55
7.53
7.52
7.48
7.47
7.46
7.36
7.03
3.82
3.81
1.02
1.93
1.05
1.96
1.14
1.10
2.00
12.5 11.5 10.5 9.5 9.0 8.5 8.0 7.5 7.0 6.5 6.0 5.5 5.0 4.5 4.0 3.5 3.0 2.5 2.0 1.5 1.0 0.5 0.0 -0.5 -1.0
f1 (ppm)

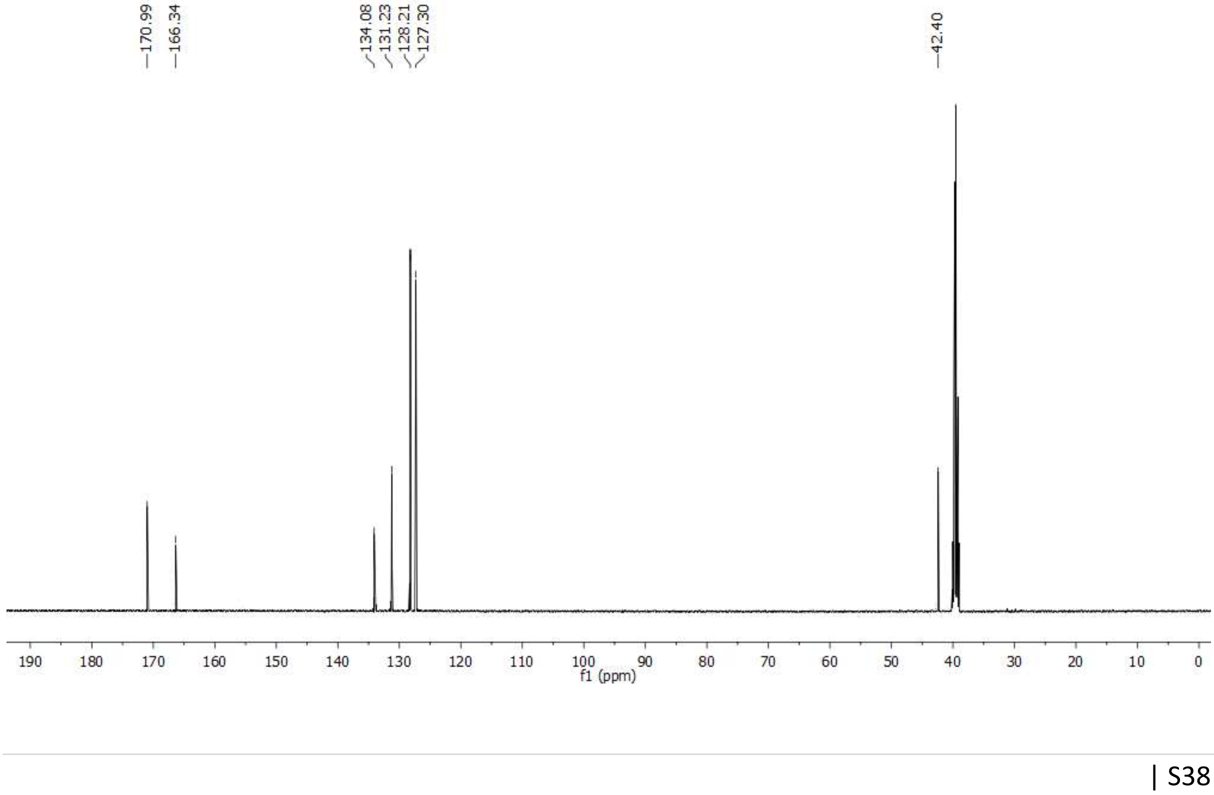
170.99
166.34
134.08
131.23
128.21
127.30
42.40
190 180 170 160 150 140 130 120 110 100 90 80 70 60 50 40 30 20 10 0
f1 (ppm)

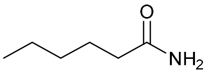
O
NH2

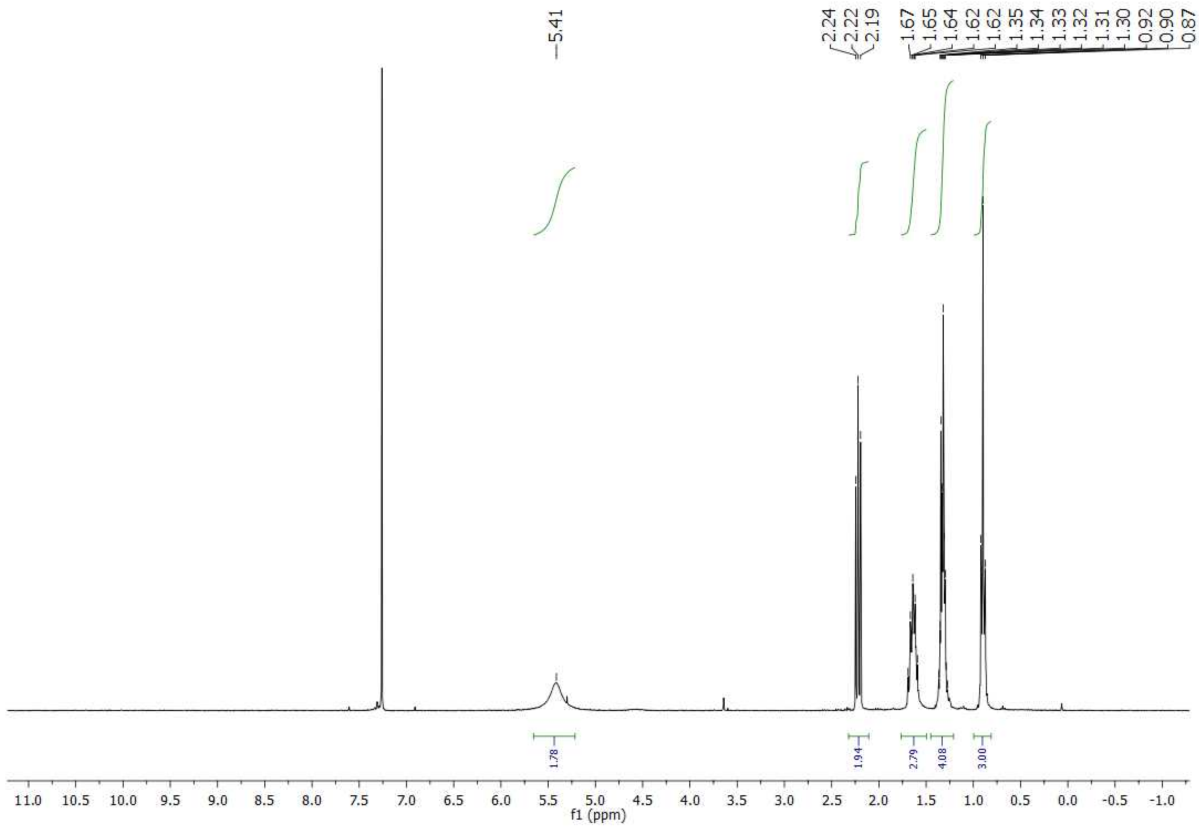
5.41
2.24
2.22
2.19
1.67
1.65
1.64
1.62
1.62
1.35
1.34
1.33
1.32
1.31
1.30
0.92
0.90
0.87
1.78
1.94
2.79
4.08
3.00
11.0 10.5 10.0 9.5 9.0 8.5 8.0 7.5 7.0 6.5 6.0 5.5 5.0 4.5 4.0 3.5 3.0 2.5 2.0 1.5 1.0 0.5 0.0 -0.5 -1.0
f1 (ppm)

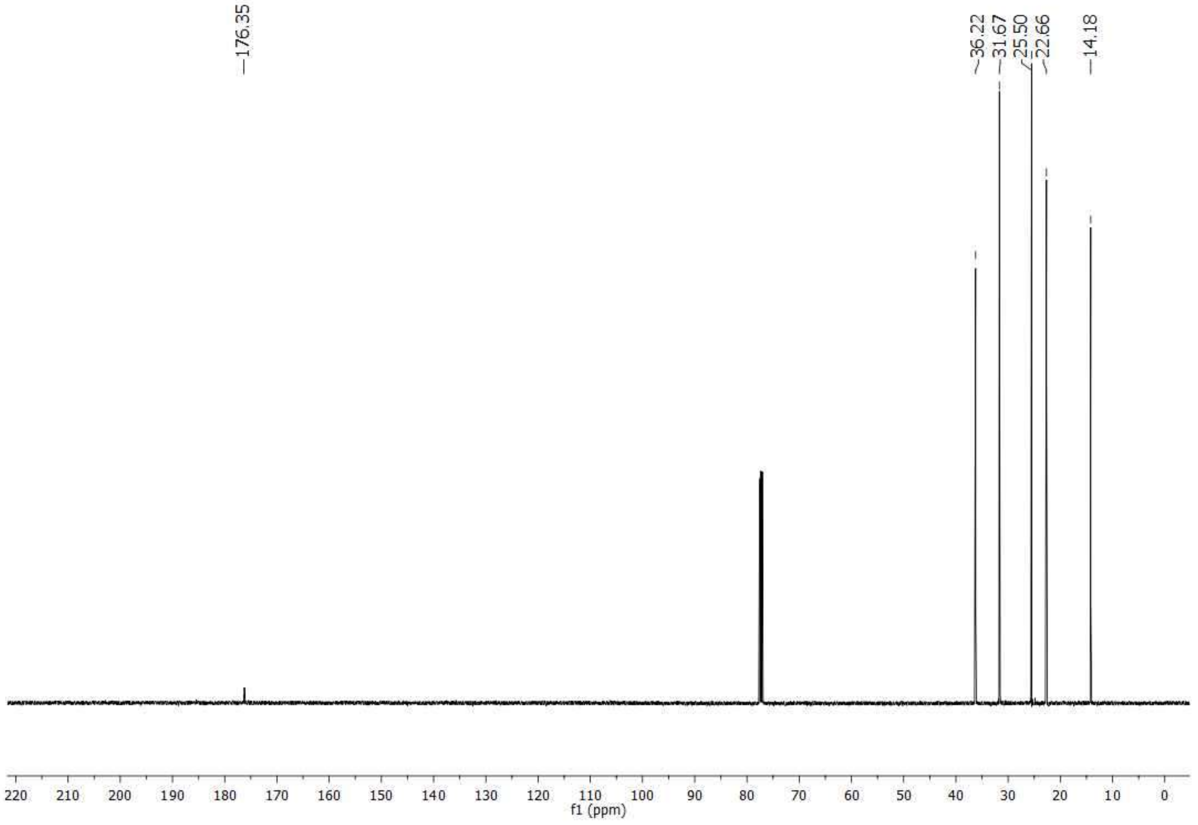
176.35
36.22
31.67
25.50
22.66
14.18
220 210 200 190 180 170 160 150 140 130 120 110 100 90 80 70 60 50 40 30 20 10 0
f1 (ppm)

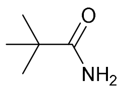

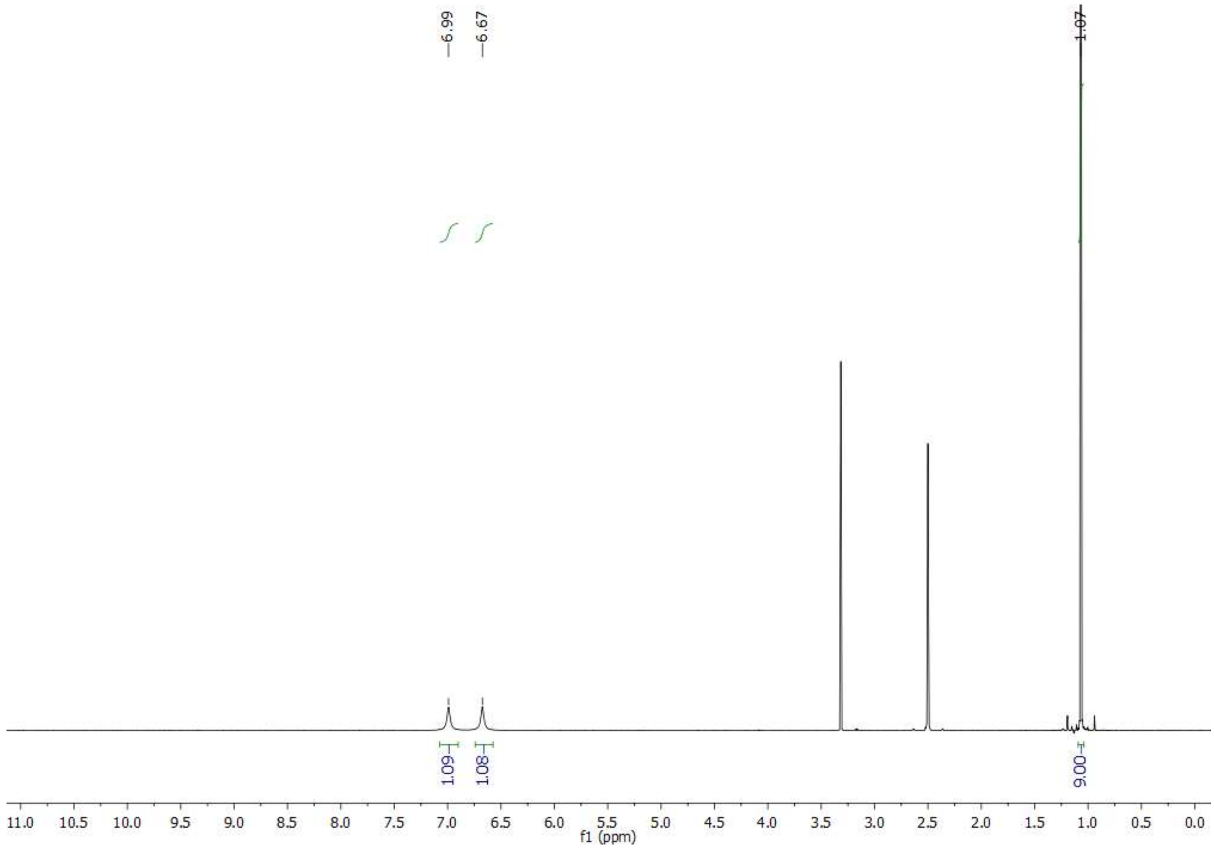
6.99
6.67
1.07
1.09
1.08
9.00
11.0 10.5 10.0 9.5 9.0 8.5 8.0 7.5 7.0 6.5 6.0 5.5 5.0 4.5 4.0 3.5 3.0 2.5 2.0 1.5 1.0 0.5 0.0
f1 (ppm)

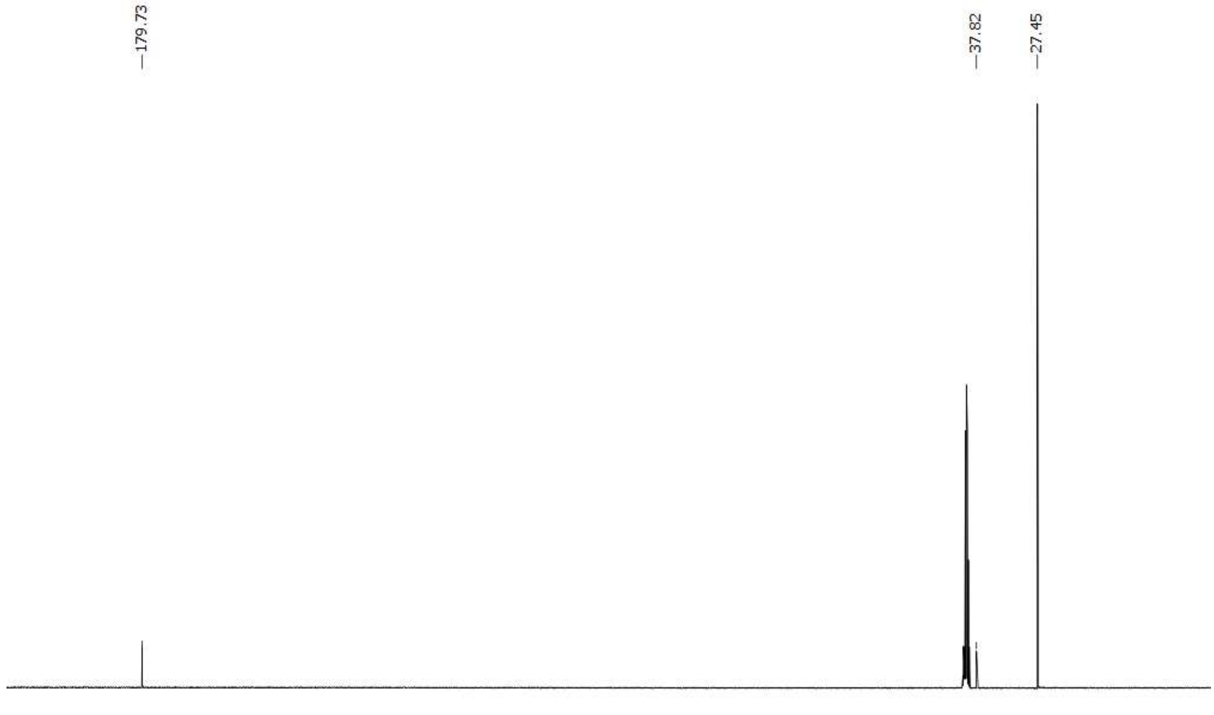
179.73
37.82
27.45
200 190 180 170 160 150 140 130 120 110 100 90 80 70 60 50 40 30 20 10 0
f1 (ppm)

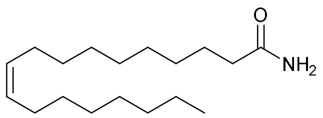
O
NH2

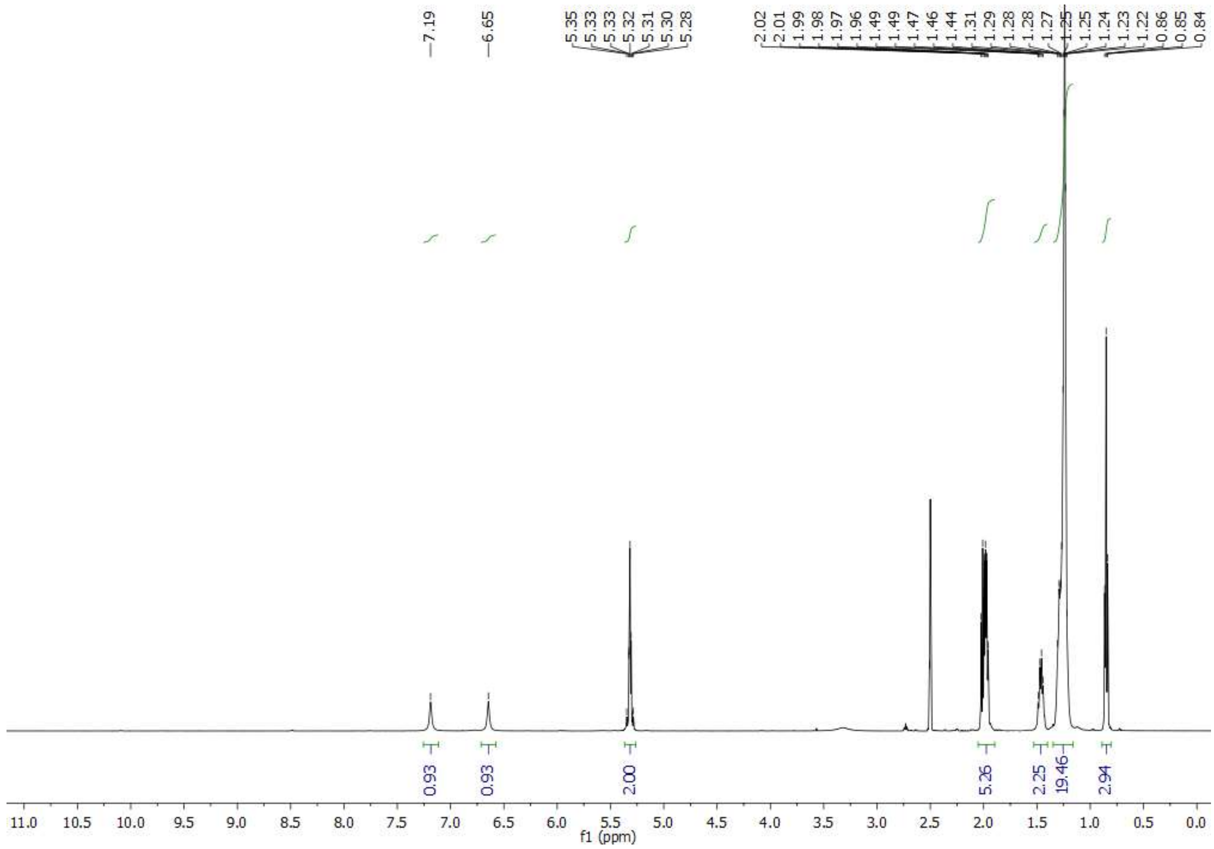
7.19
6.65
5.35
5.33
5.33
5.32
5.31
5.30
5.28
2.02
2.01
1.99
1.98
1.97
1.96
1.49
1.49
1.47
1.46
1.44
1.31
1.29
1.28
1.28
1.27
1.25
1.25
1.24
1.23
1.22
0.86
0.85
0.84
0.93
0.93
2.00
5.26
2.25
19.46
2.94
11.0 10.5 10.0 9.5 9.0 8.5 8.0 7.5 7.0 6.5 6.0 5.5 5.0 4.5 4.0 3.5 3.0 2.5 2.0 1.5 1.0 0.5 0.0
f1 (ppm)

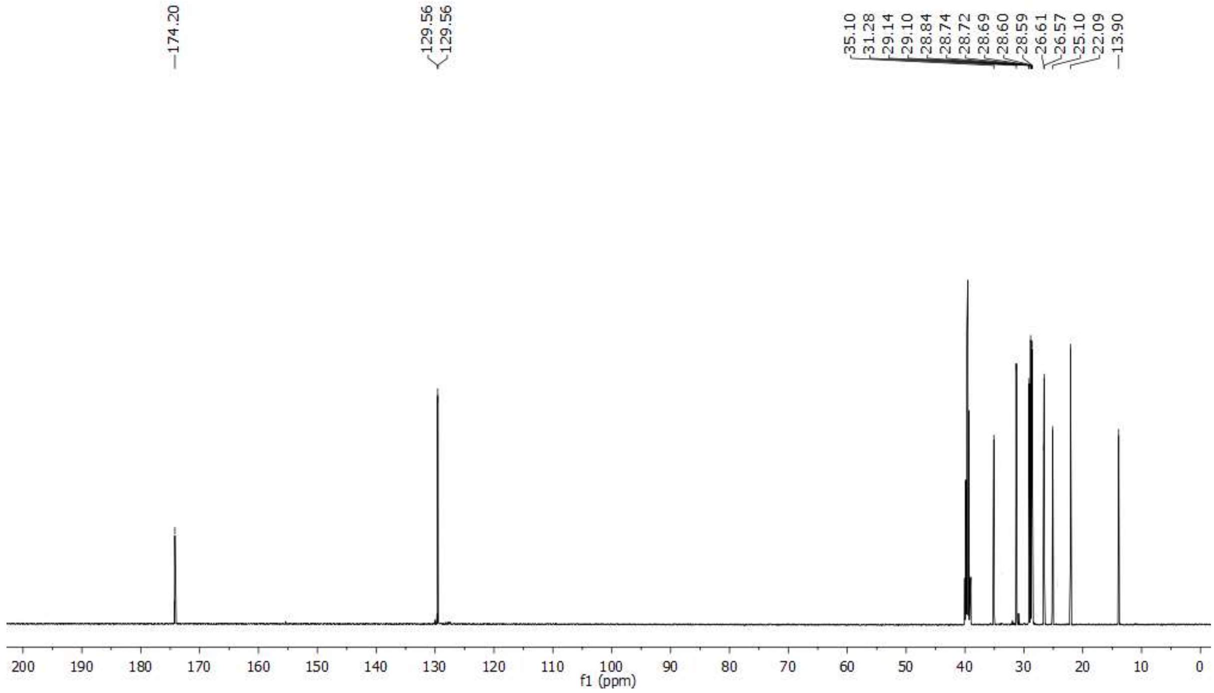
174.20
129.56
129.56
35.10
31.28
29.14
29.10
28.84
28.74
28.72
28.69
28.60
28.59
26.61
26.57
25.10
22.09
13.90
200 190 180 170 160 150 140 130 120 110 100 90 80 70 60 50 40 30 20 10 0
f1 (ppm)

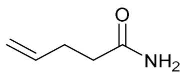
O
NH2

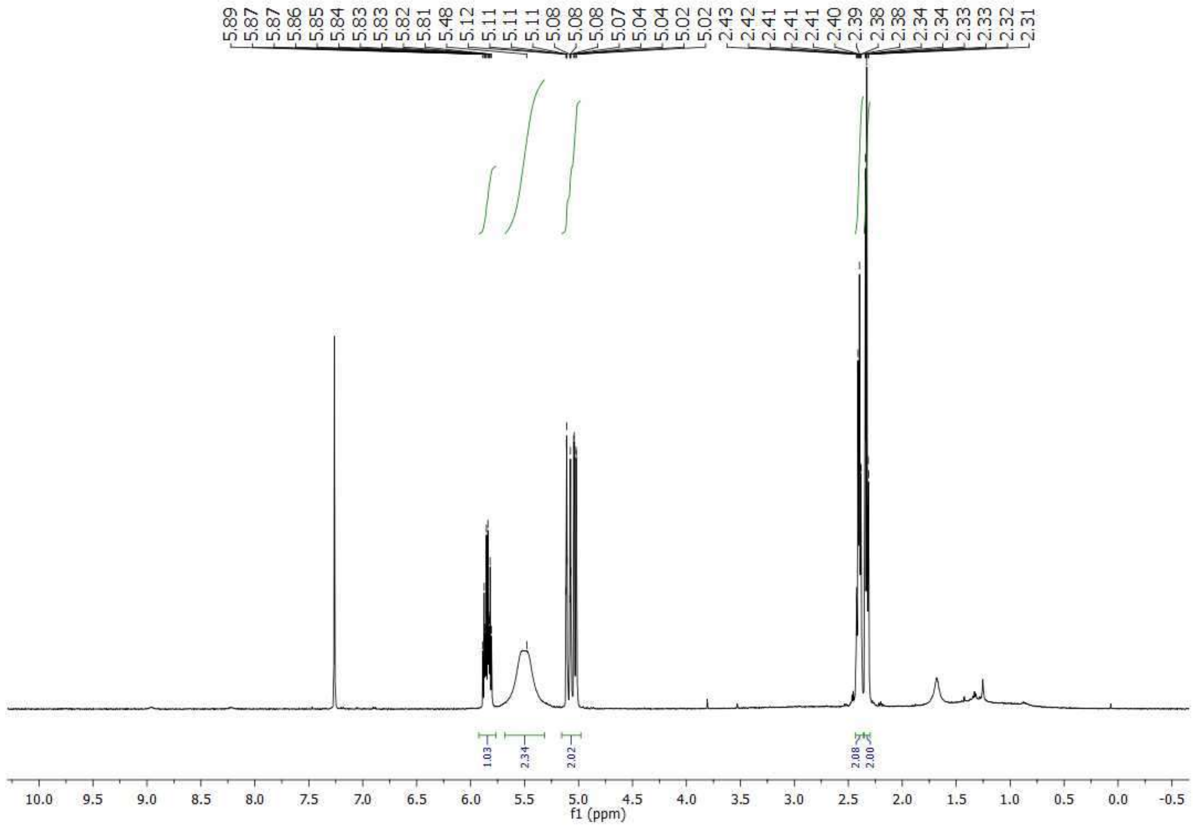
5.89
5.87
5.87
5.86
5.85
5.84
5.83
5.83
5.82
5.81
5.48
5.12
5.11
5.11
5.11
5.08
5.08
5.07
5.04
5.04
5.02
5.02
2.43
2.42
2.41
2.41
2.40
2.39
2.38
2.38
2.34
2.34
2.33
2.33
2.32
2.31
1.03
2.34
2.02
2.08
2.00
10.0 9.5 9.0 8.5 8.0 7.5 7.0 6.5 6.0 5.5 5.0 4.5 4.0 3.5 3.0 2.5 2.0 1.5 1.0 0.5 0.0 -0.5
f1 (ppm)

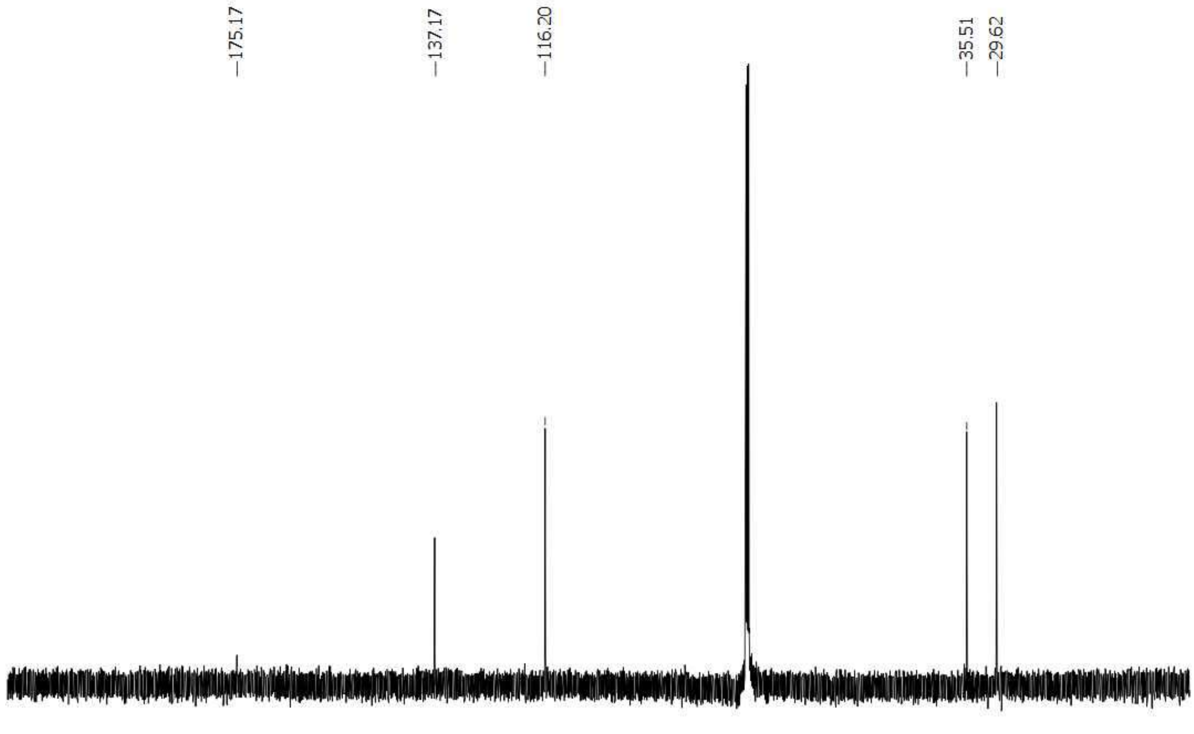
175.17
137.17
116.20
35.51
29.62
210 200 190 180 170 160 150 140 130 120 110 100 90 80 70 60 50 40 30 20 10 0
f1 (ppm)

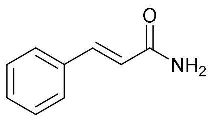
O
NH2

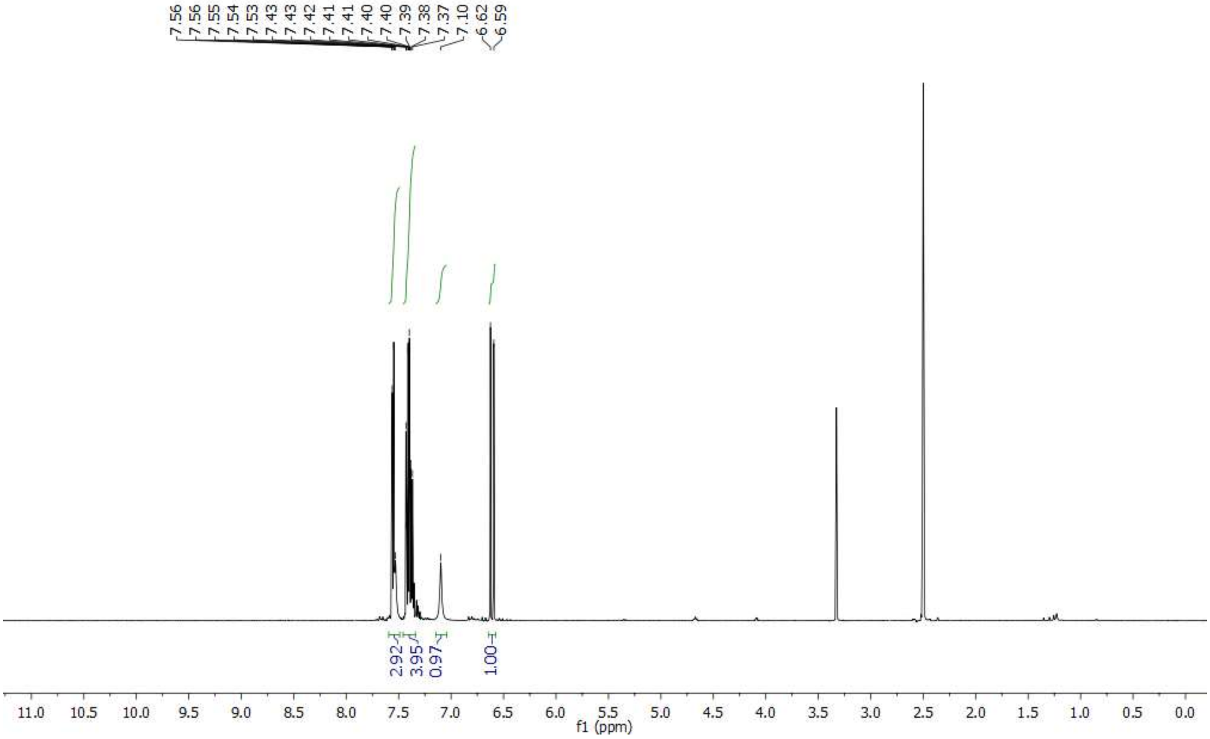
7.56
7.56
7.55
7.54
7.53
7.43
7.43
7.42
7.41
7.41
7.40
7.40
7.39
7.38
7.37
7.10
6.62
6.59
2.92
3.95
0.97
1.00
11.0 10.5 10.0 9.5 9.0 8.5 8.0 7.5 7.0 6.5 6.0 5.5 5.0 4.5 4.0 3.5 3.0 2.5 2.0 1.5 1.0 0.5 0.0
f1 (ppm)

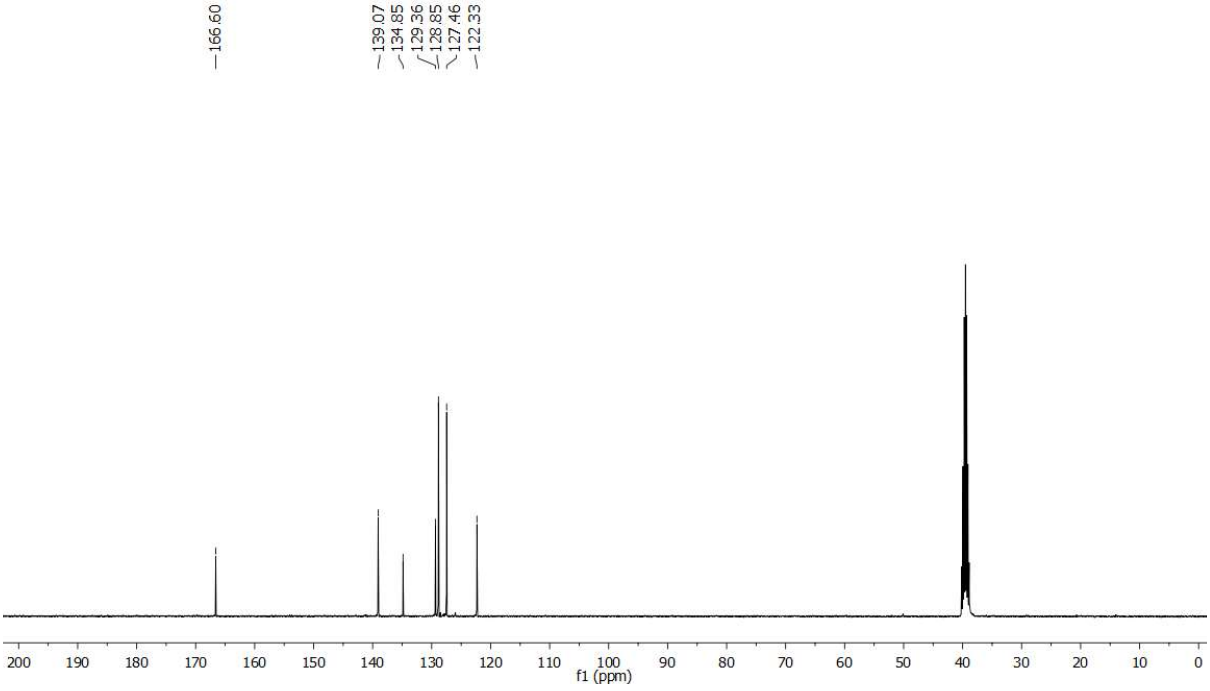
166.60
139.07
134.85
129.36
128.85
127.46
122.33
200 190 180 170 160 150 140 130 120 110 100 90 80 70 60 50 40 30 20 10 0
f1 (ppm)

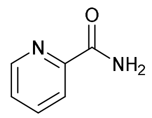
N
O
NH2

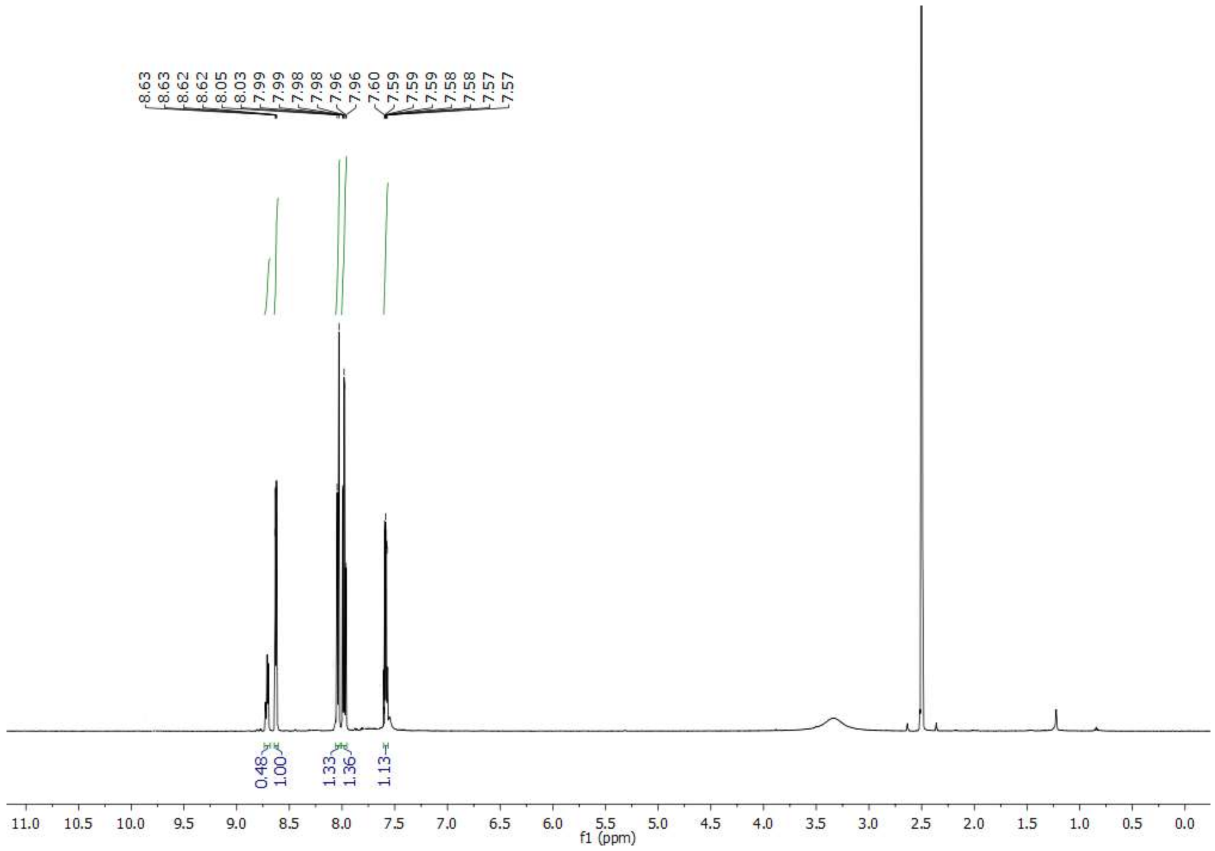
8.63
8.63
8.62
8.62
8.05
8.03
7.99
7.99
7.98
7.98
7.96
7.96
7.60
7.59
7.59
7.59
7.58
7.58
7.57
7.57
0.48
1.00
1.33
1.36
1.13
f1 (ppm)

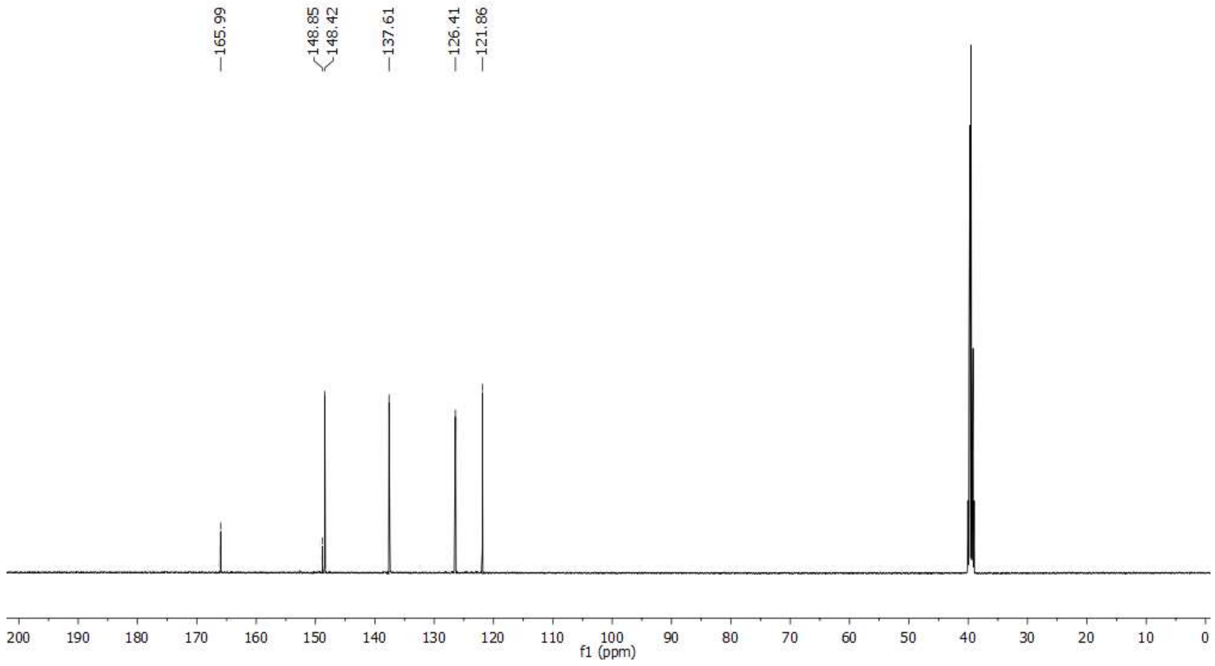
165.99
148.85
148.42
137.61
126.41
121.86
f1 (ppm)

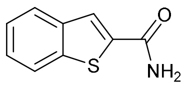
O
S
NH2

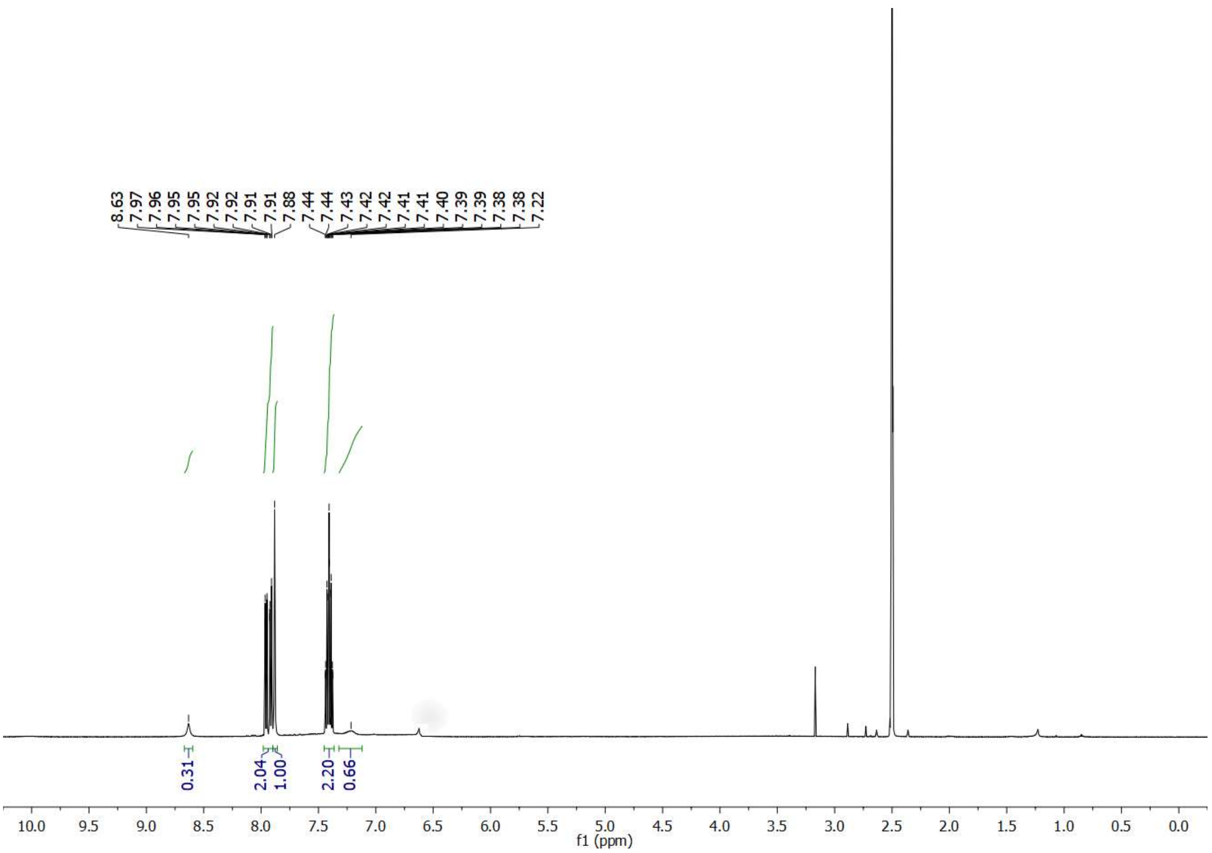
8.63
7.97
7.96
7.95
7.95
7.92
7.92
7.91
7.91
7.88
7.44
7.44
7.43
7.42
7.42
7.41
7.41
7.40
7.39
7.39
7.38
7.38
7.22
0.31
2.04
1.00
2.20
0.66
10.0 9.5 9.0 8.5 8.0 7.5 7.0 6.5 6.0 5.5 5.0 4.5 4.0 3.5 3.0 2.5 2.0 1.5 1.0 0.5 0.0
f1 (ppm)

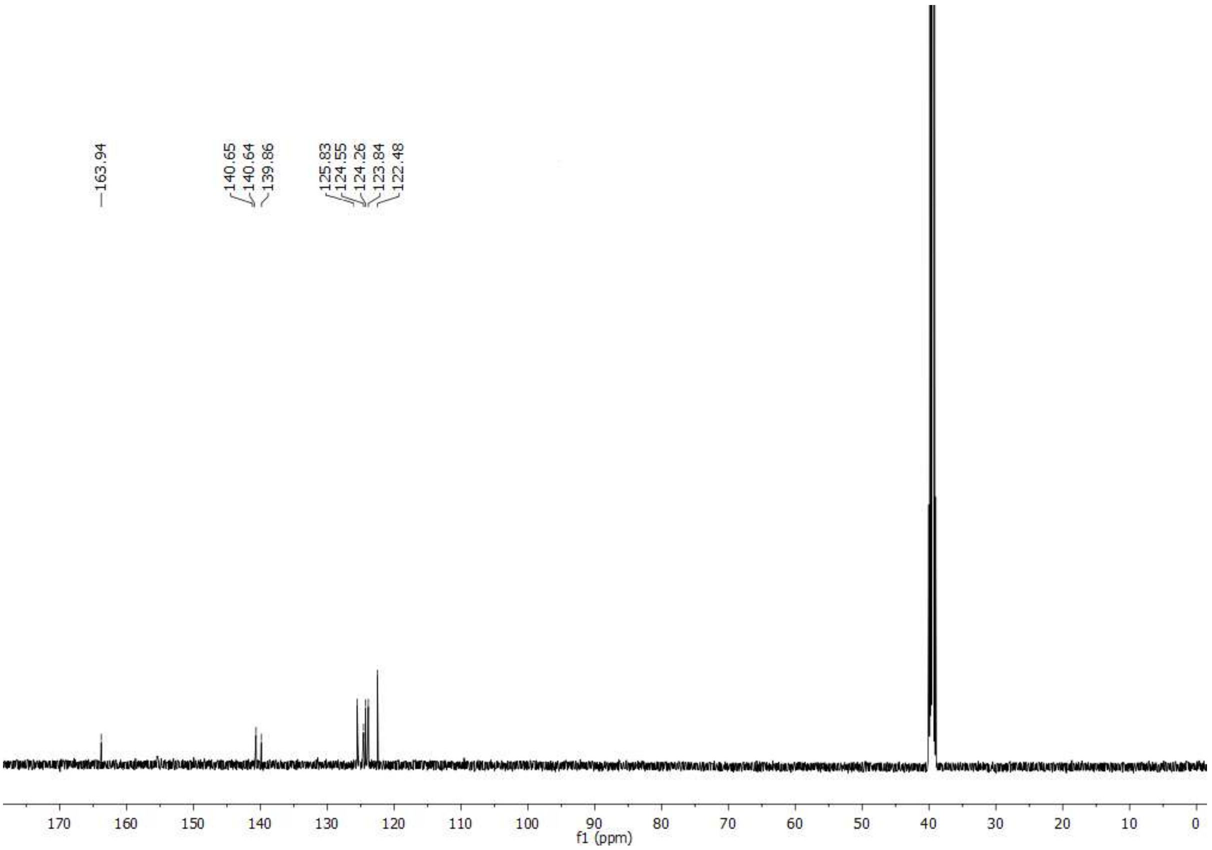
163.94
140.65
140.64
139.86
125.83
124.55
124.26
123.84
122.48
170 160 150 140 130 120 110 100 90 80 70 60 50 40 30 20 10 0
f1 (ppm)

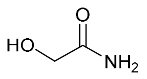

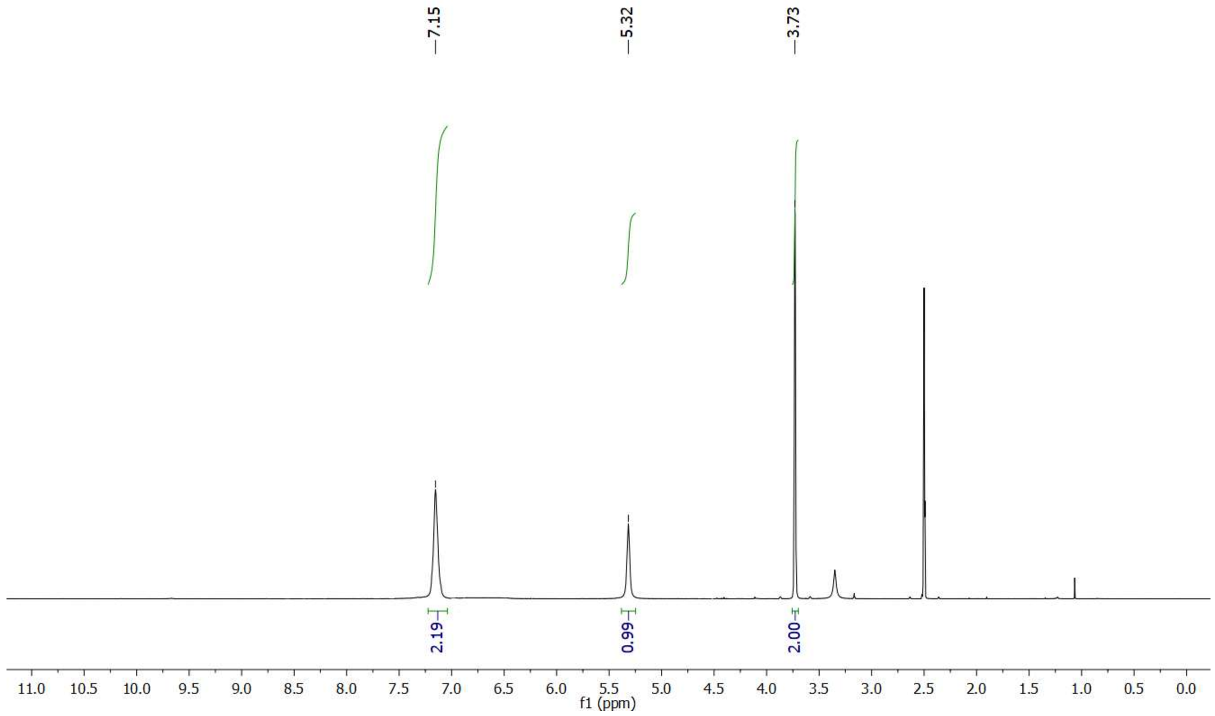

7.15
5.32
3.73
2.19
0.99
2.00
11.0 10.5 10.0 9.5 9.0 8.5 8.0 7.5 7.0 6.5 6.0 5.5 5.0 4.5 4.0 3.5 3.0 2.5 2.0 1.5 1.0 0.5 0.0
f1 (ppm)

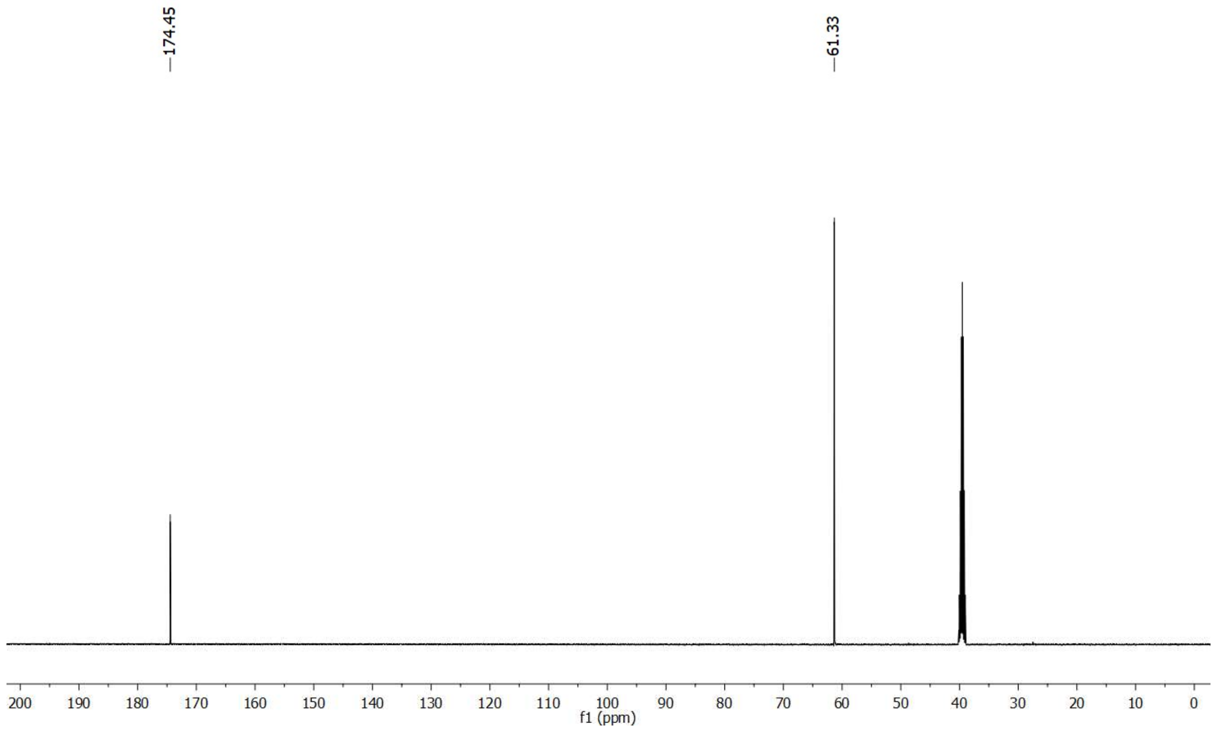

174.45
61.33
200 190 180 170 160 150 140 130 120 110 100 90 80 70 60 50 40 30 20 10 0
f1 (ppm)

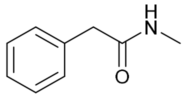
H
N
O

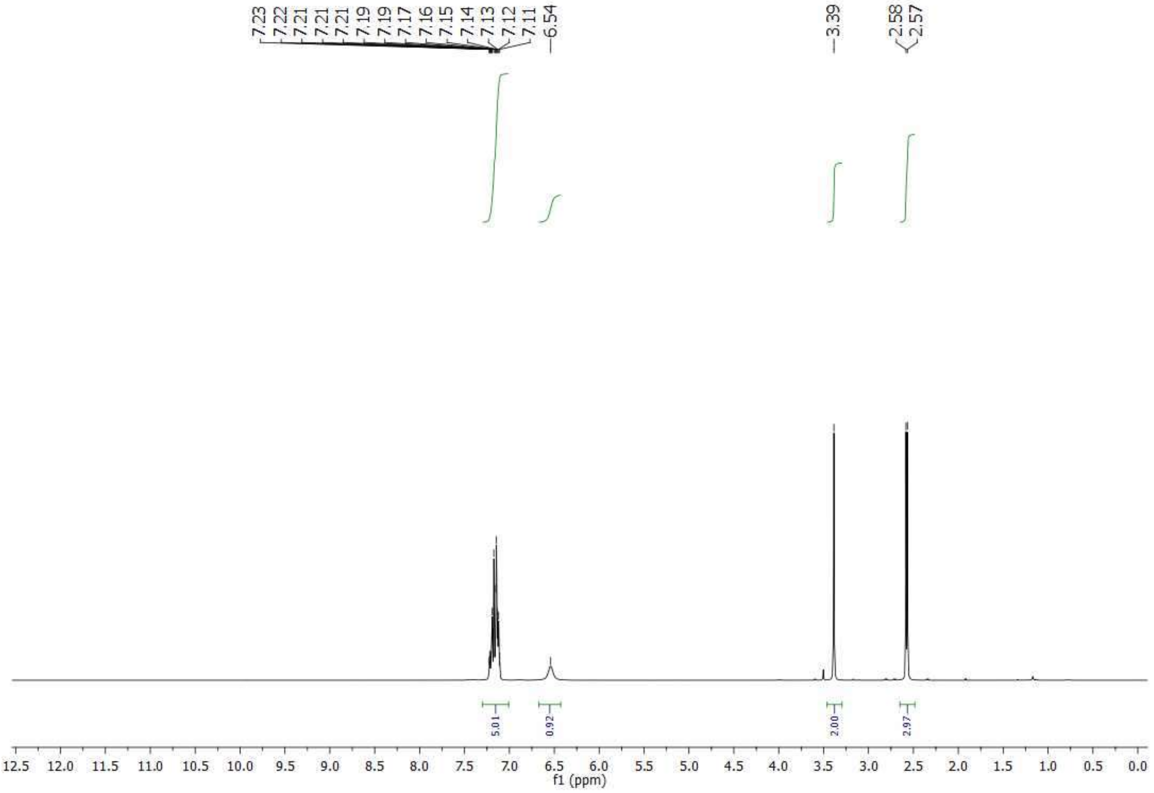
7.23
7.22
7.21
7.21
7.21
7.19
7.19
7.17
7.16
7.15
7.14
7.13
7.12
7.11
6.54
3.39
2.58
2.57
5.01
0.92
2.00
2.97
12.5 12.0 11.5 11.0 10.5 10.0 9.5 9.0 8.5 8.0 7.5 7.0 6.5 6.0 5.5 5.0 4.5 4.0 3.5 3.0 2.5 2.0 1.5 1.0 0.5 0.0
f1 (ppm)

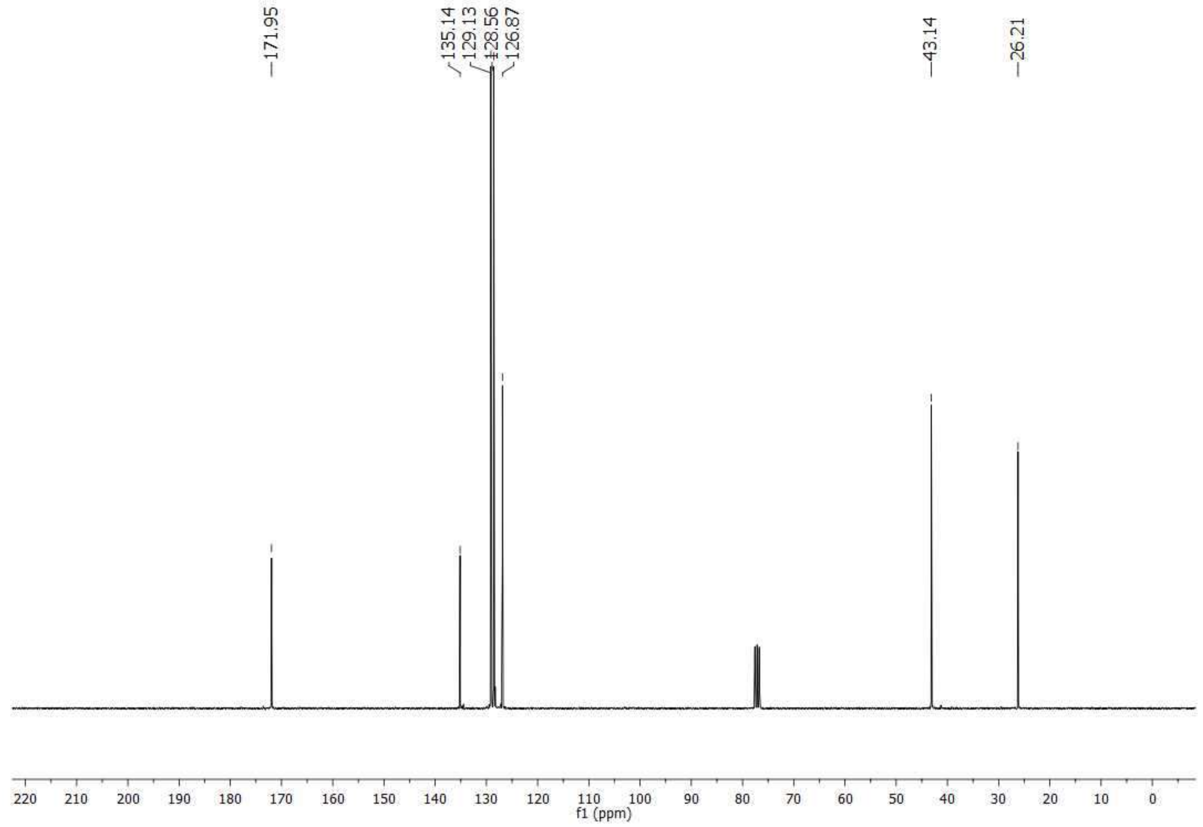
171.95
135.14
129.13
128.56
126.87
43.14
26.21
220 210 200 190 180 170 160 150 140 130 120 110 100 90 80 70 60 50 40 30 20 10 0
f1 (ppm)

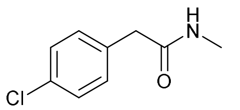
H
N
O
Cl

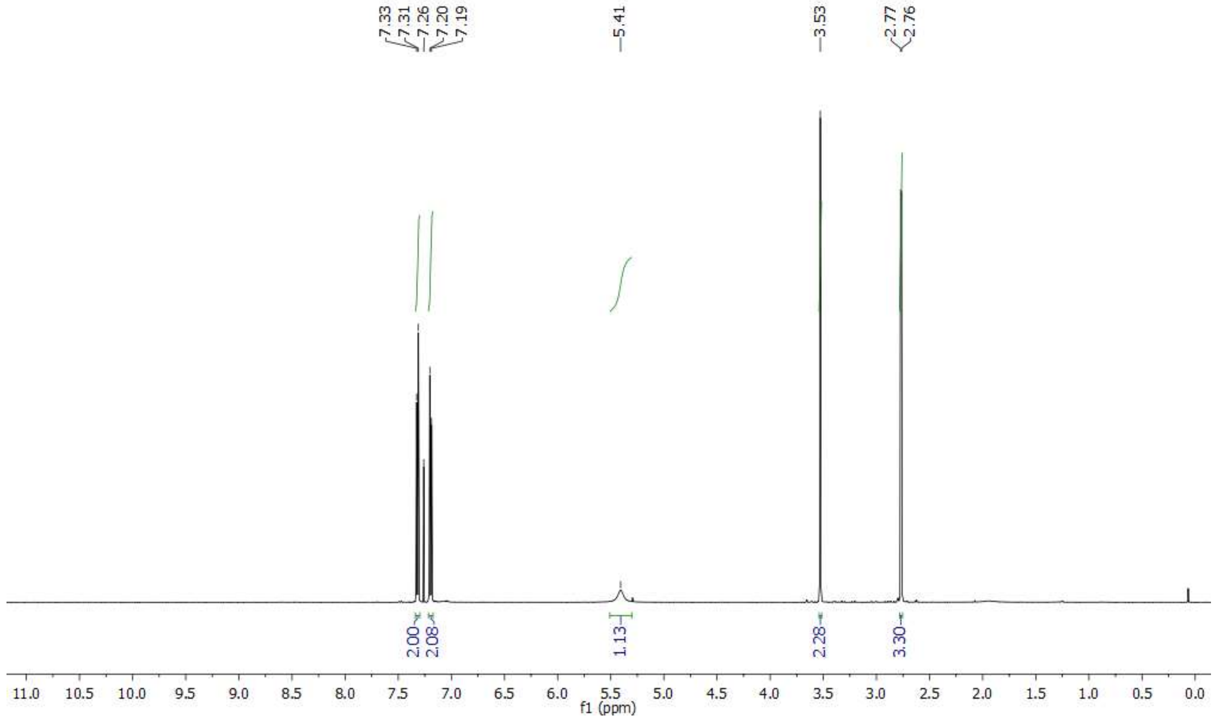
7.33
7.31
7.26
7.20
7.19
5.41
3.53
2.77
2.76
2.00
2.08
1.13
2.28
3.30
11.0 10.5 10.0 9.5 9.0 8.5 8.0 7.5 7.0 6.5 6.0 5.5 5.0 4.5 4.0 3.5 3.0 2.5 2.0 1.5 1.0 0.5 0.0
f1 (ppm)

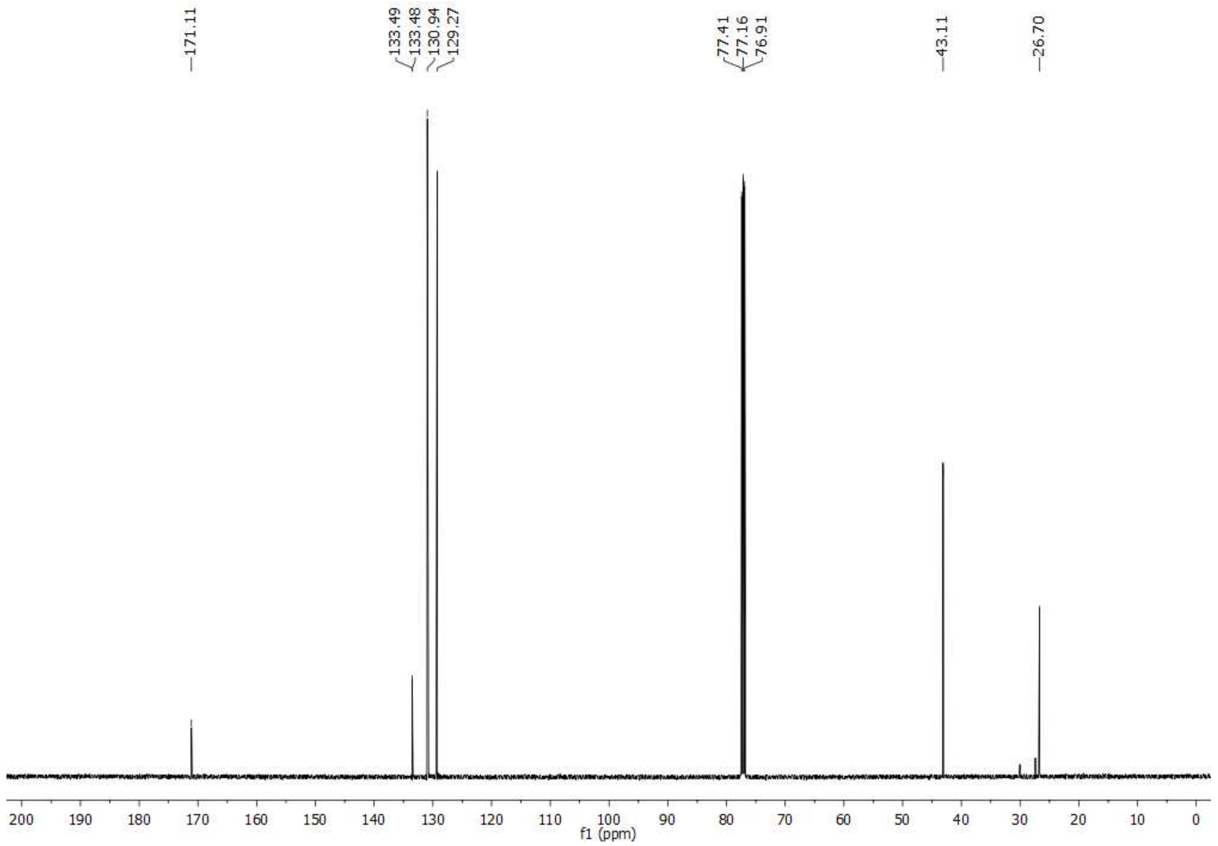
171.11
133.49
133.48
130.94
129.27
77.41
77.16
76.91
43.11
26.70
200 190 180 170 160 150 140 130 120 110 100 90 80 70 60 50 40 30 20 10 0
f1 (ppm)

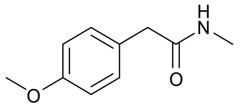
H
N
O
O

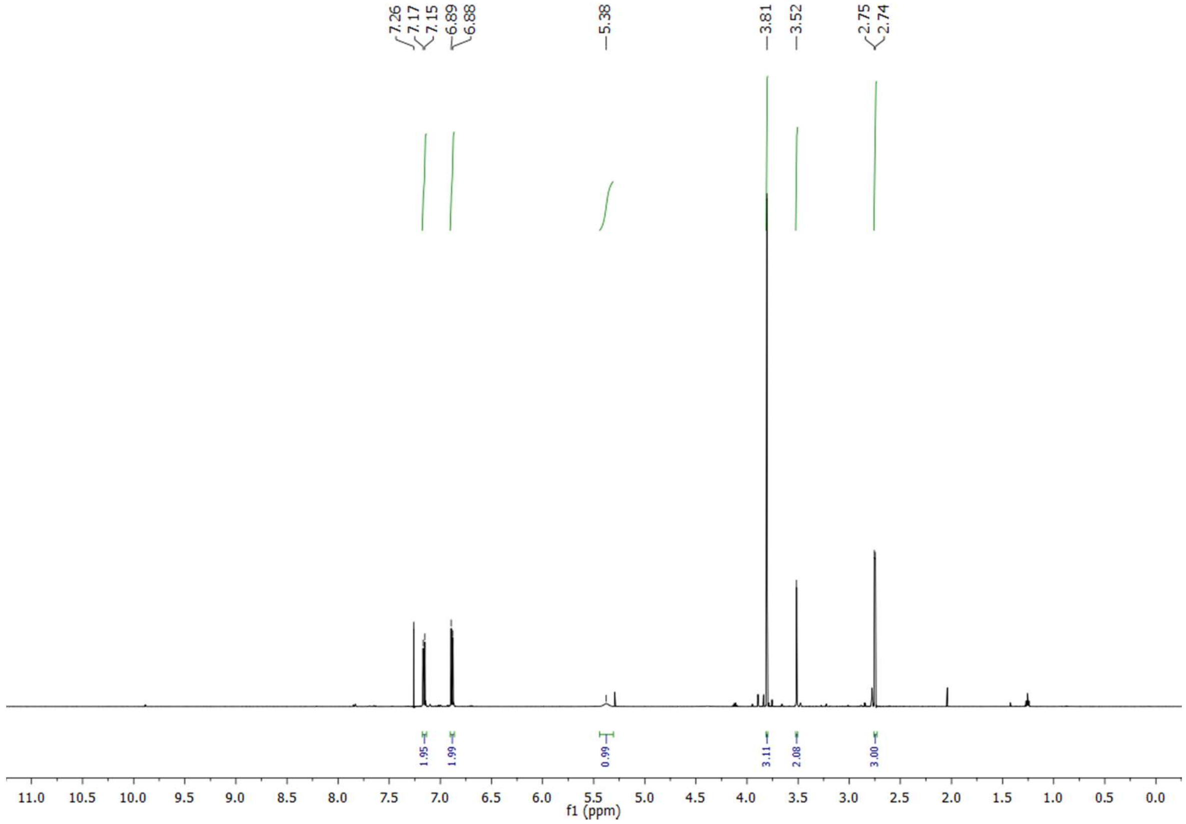
7.26
7.17
7.15
6.89
6.88
5.38
3.81
3.52
2.75
2.74
1.95
1.99
0.99
3.11
2.08
3.00
11.0 10.5 10.0 9.5 9.0 8.5 8.0 7.5 7.0 6.5 6.0 5.5 5.0 4.5 4.0 3.5 3.0 2.5 2.0 1.5 1.0 0.5 0.0
f1 (ppm)

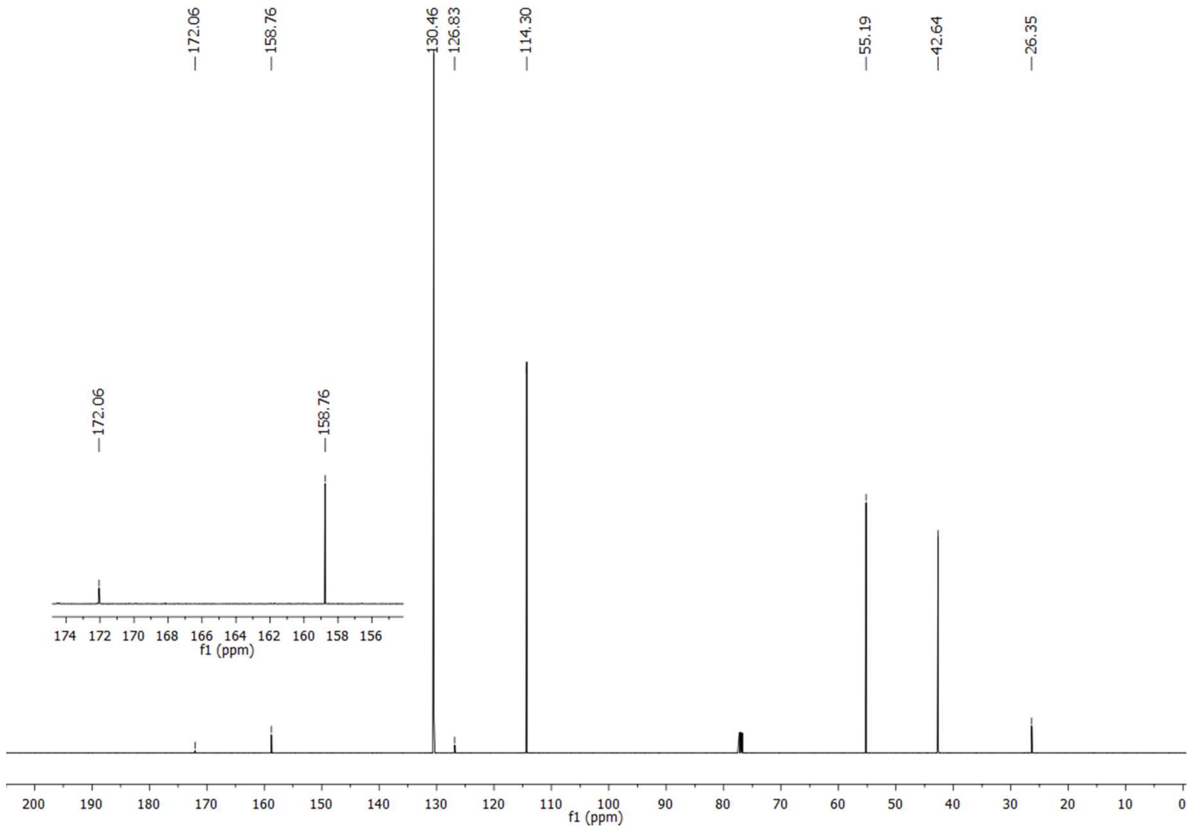
172.06
158.76
130.46
126.83
114.30
55.19
42.64
26.35
172.06
158.76
174 172 170 168 166 164 162 160 158 156
f1 (ppm)
200 190 180 170 160 150 140 130 120 110 100 90 80 70 60 50 40 30 20 10 0
f1 (ppm)

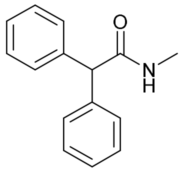
O
N
H

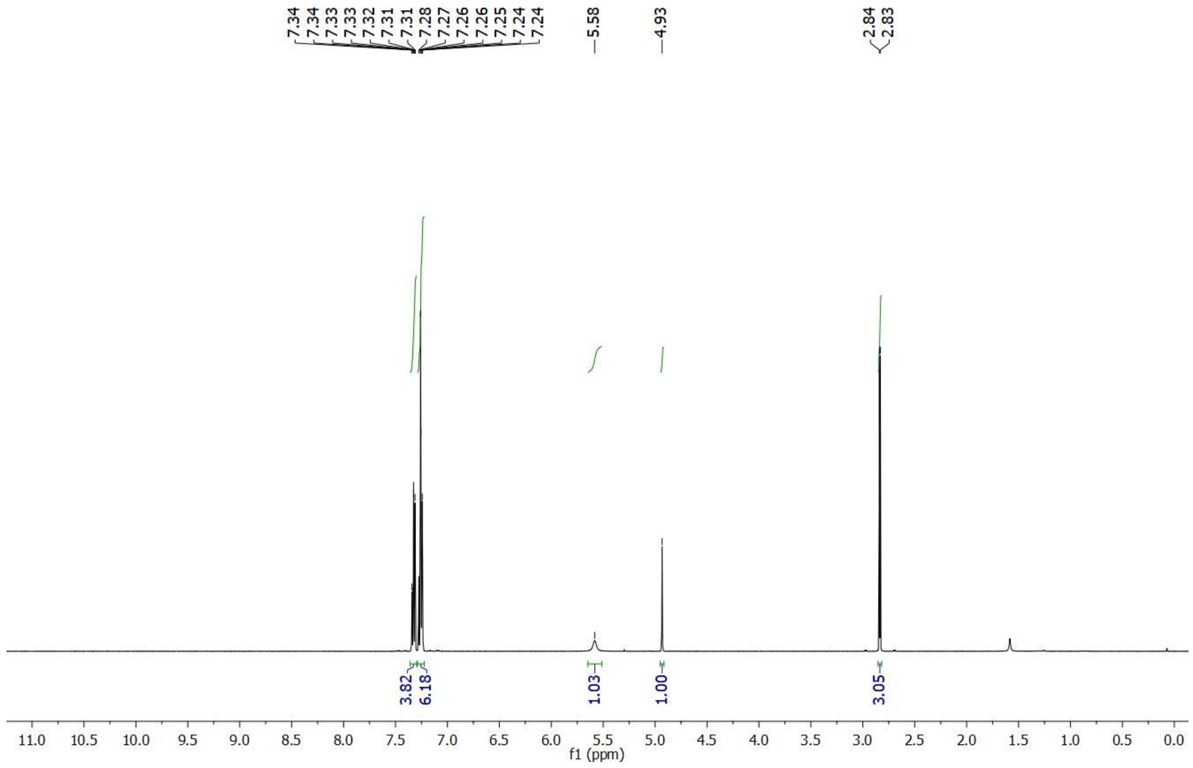
7.34
7.34
7.33
7.33
7.32
7.31
7.31
7.28
7.27
7.26
7.26
7.25
7.24
7.24
5.58
4.93
2.84
2.83
3.82
6.18
1.03
1.00
3.05
11.0 10.5 10.0 9.5 9.0 8.5 8.0 7.5 7.0 6.5 6.0 5.5 5.0 4.5 4.0 3.5 3.0 2.5 2.0 1.5 1.0 0.5 0.0
f1 (ppm)

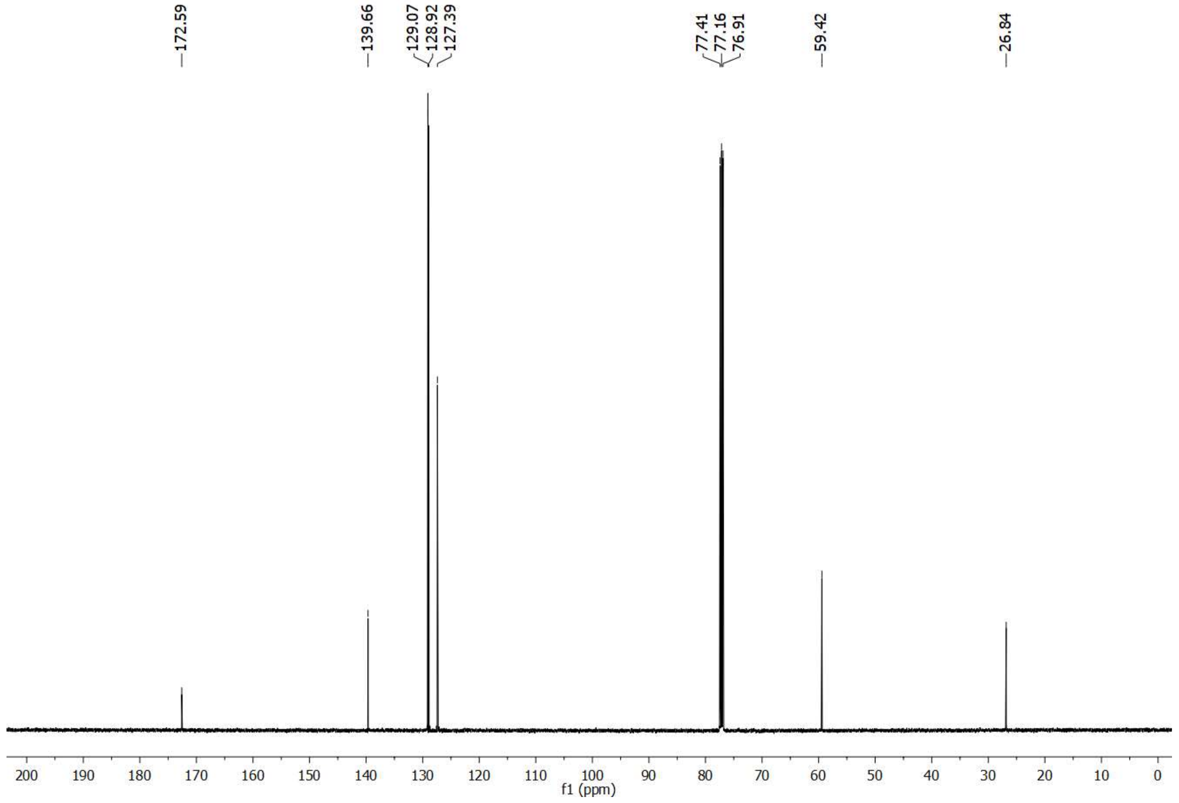
172.59
139.66
129.07
128.92
127.39
77.41
77.16
76.91
59.42
26.84
200 190 180 170 160 150 140 130 120 110 100 90 80 70 60 50 40 30 20 10 0
f1 (ppm)

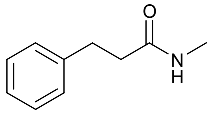

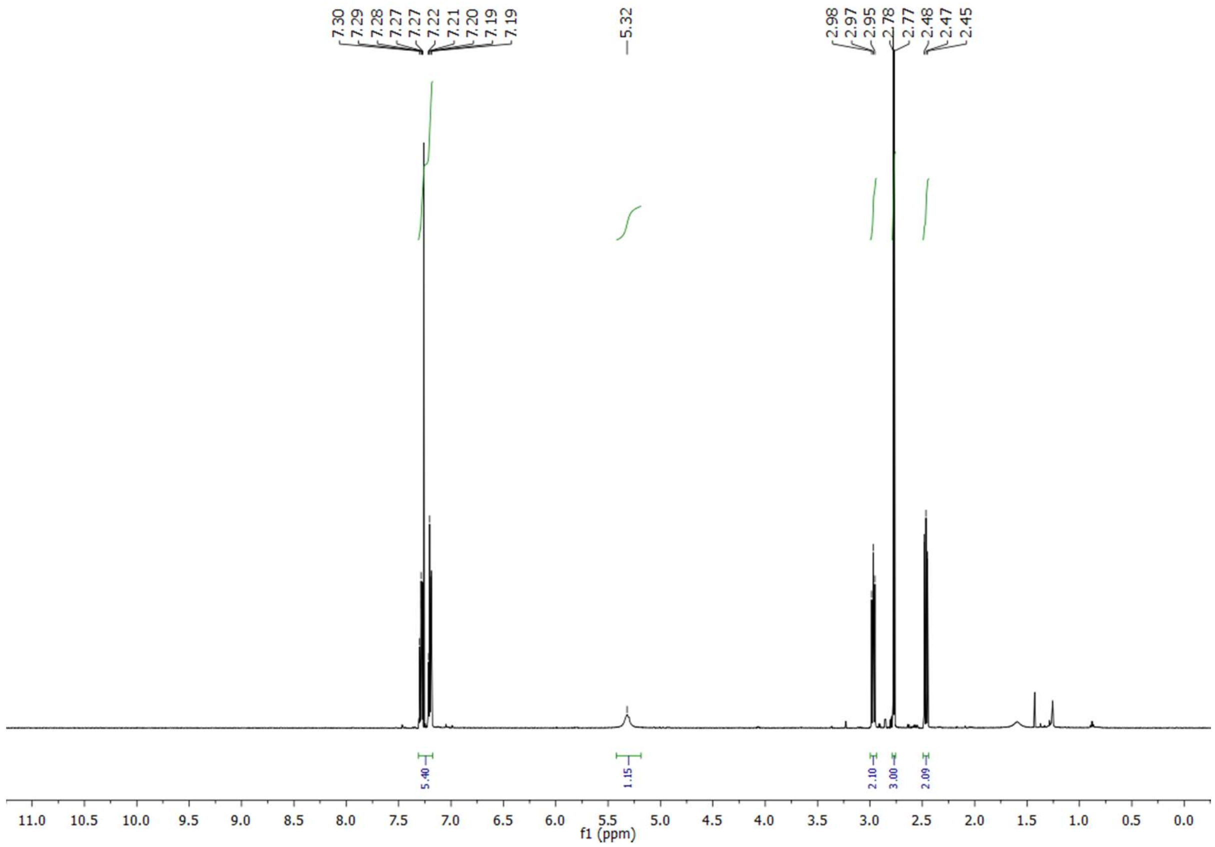

7.30
7.29
7.28
7.27
7.27
7.22
7.21
7.20
7.19
7.19
5.32
2.98
2.97
2.95
2.78
2.77
2.48
2.47
2.45
5.40
1.15
2.10
3.00
2.09
f1 (ppm)

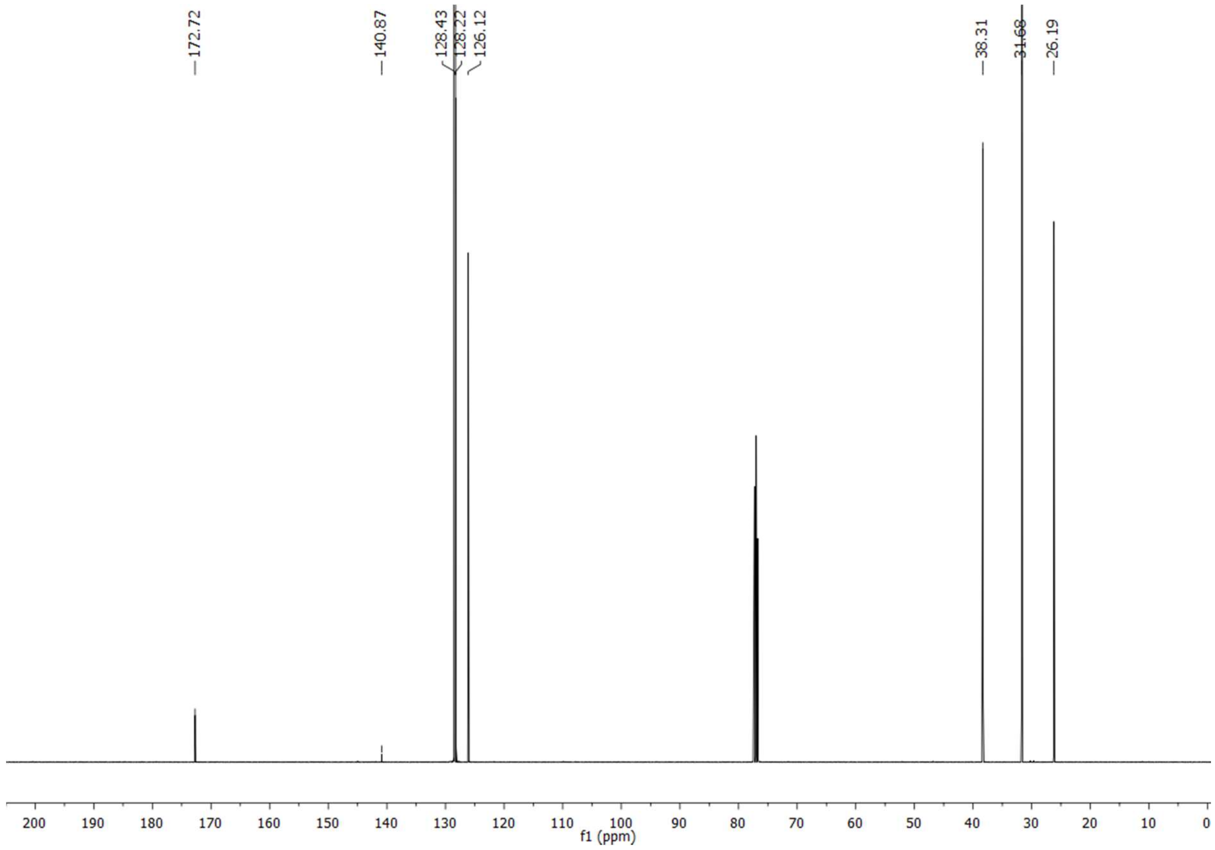

172.72
140.87
128.43
128.22
126.12
38.31
31.68
26.19
f1 (ppm)

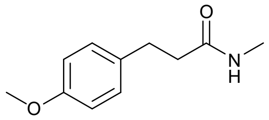
O
N
H
O

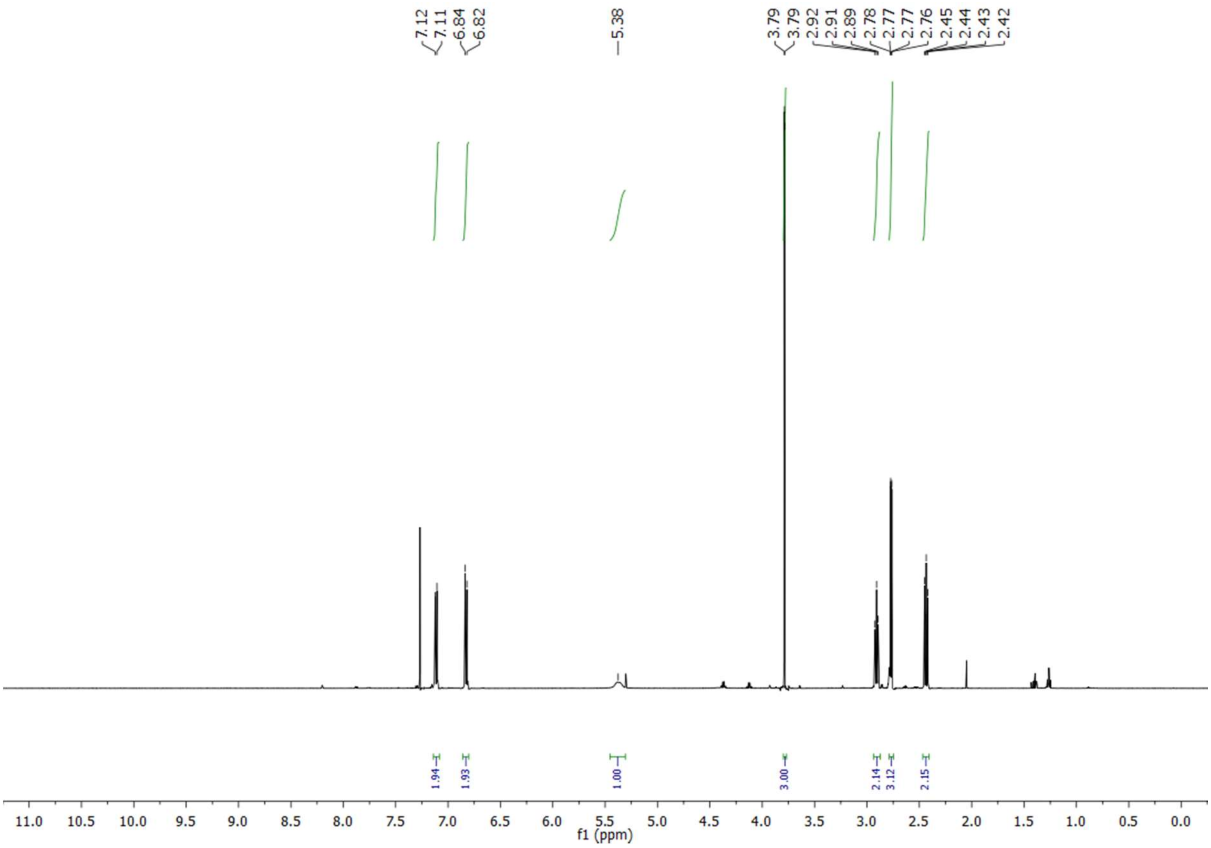
7.12
7.11
6.84
6.82
5.38
3.79
3.79
2.92
2.91
2.89
2.78
2.77
2.77
2.76
2.45
2.44
2.43
2.42
1.94
1.93
1.00
3.00
2.14
3.12
2.15
f1 (ppm)

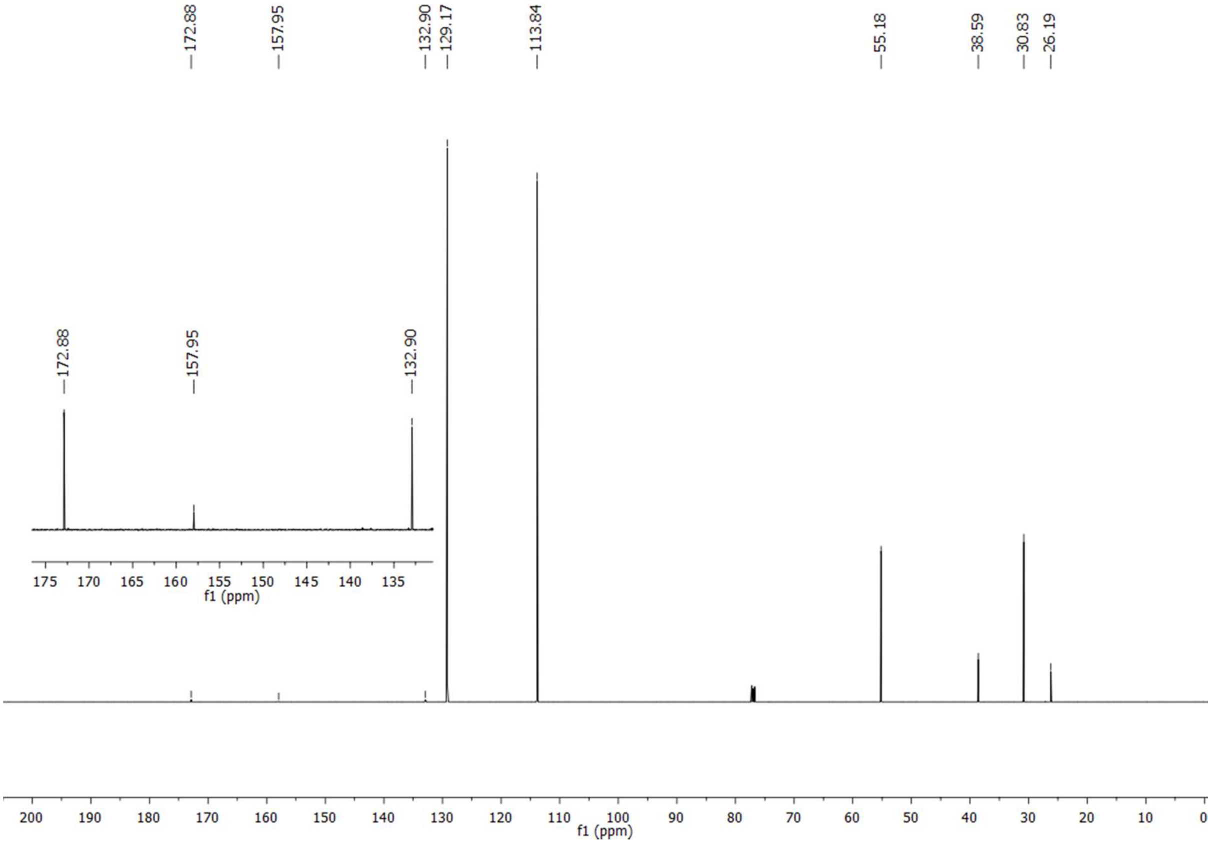
172.88
157.95
132.90
129.17
113.84
55.18
38.59
30.83
26.19
f1 (ppm)

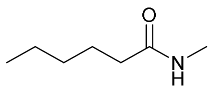
O
N
H

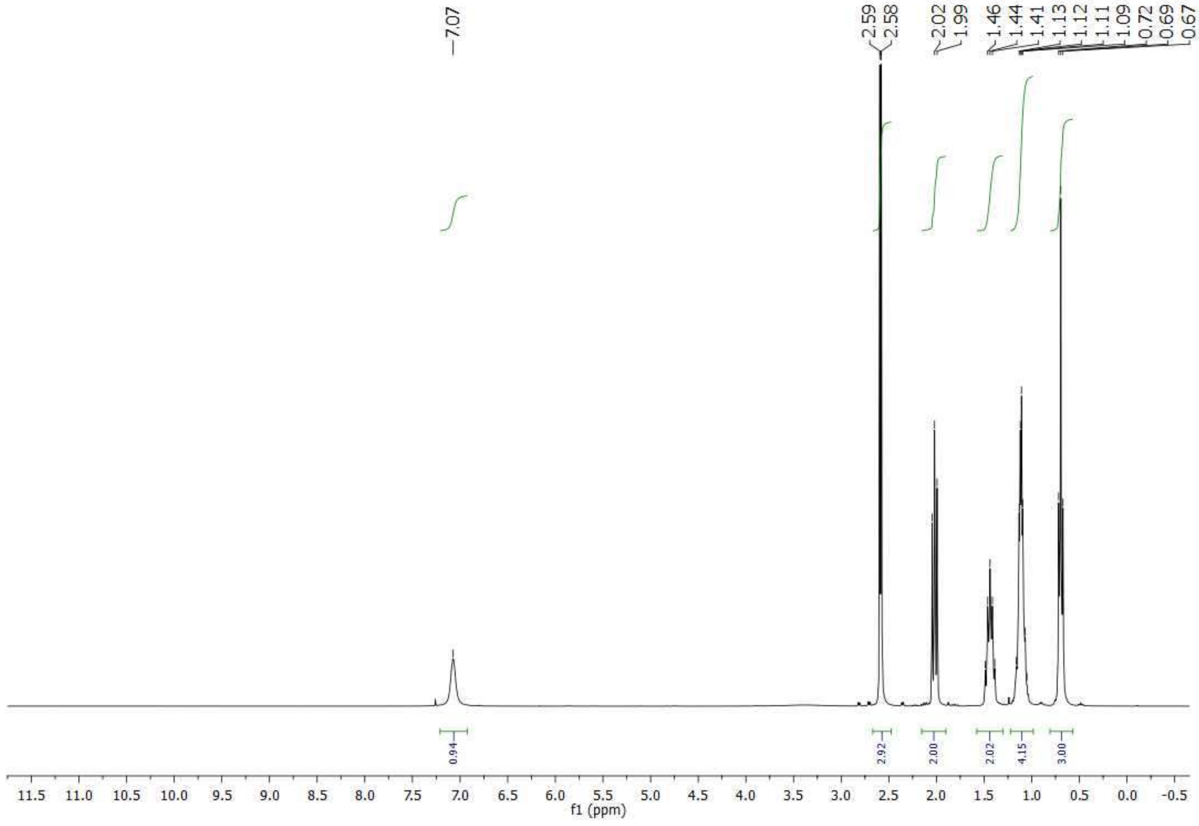
7.07
2.59
2.58
2.02
1.99
1.46
1.44
1.41
1.13
1.12
1.11
1.09
0.72
0.69
0.67
0.94
2.92
2.00
2.02
4.15
3.00
11.5 11.0 10.5 10.0 9.5 9.0 8.5 8.0 7.5 7.0 6.5 6.0 5.5 5.0 4.5 4.0 3.5 3.0 2.5 2.0 1.5 1.0 0.5 0.0 -0.5
f1 (ppm)

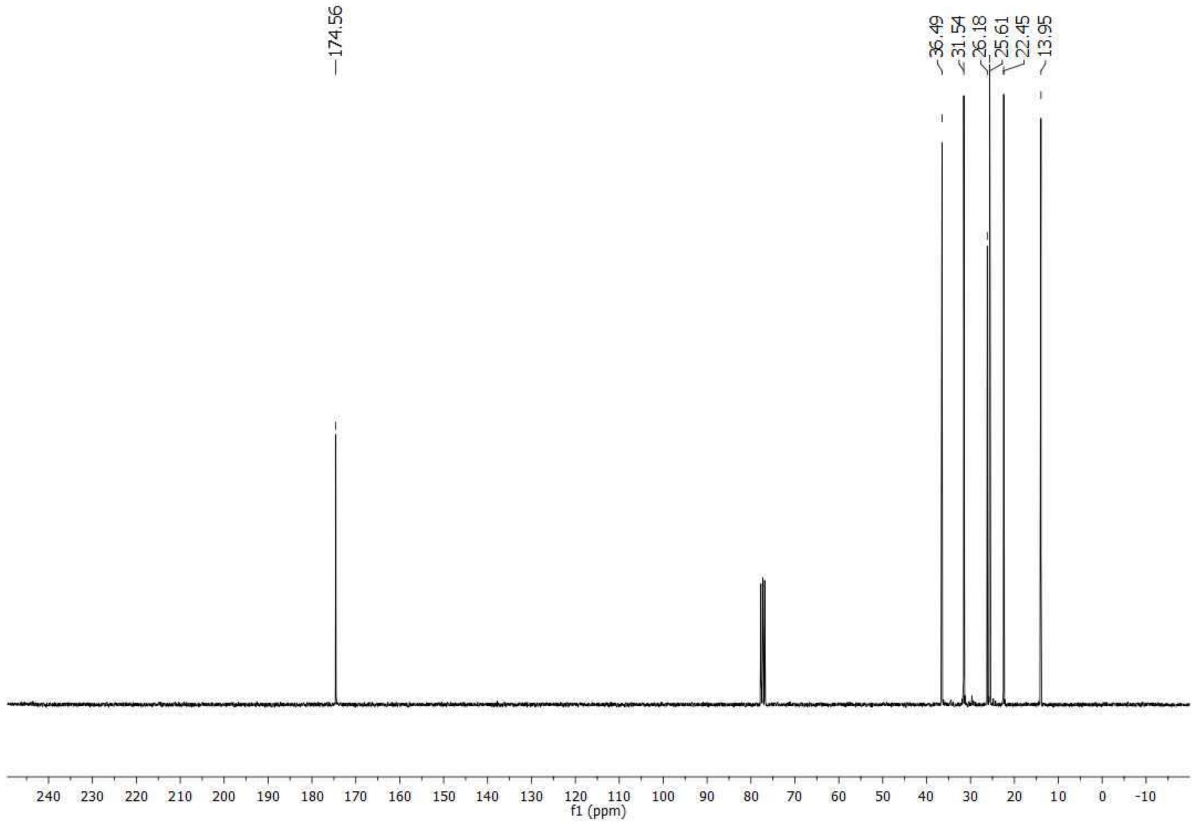
174.56
36.49
31.54
26.18
25.61
22.45
13.95
240 230 220 210 200 190 180 170 160 150 140 130 120 110 100 90 80 70 60 50 40 30 20 10 0 -10
f1 (ppm)

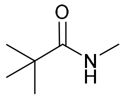
O
N
H

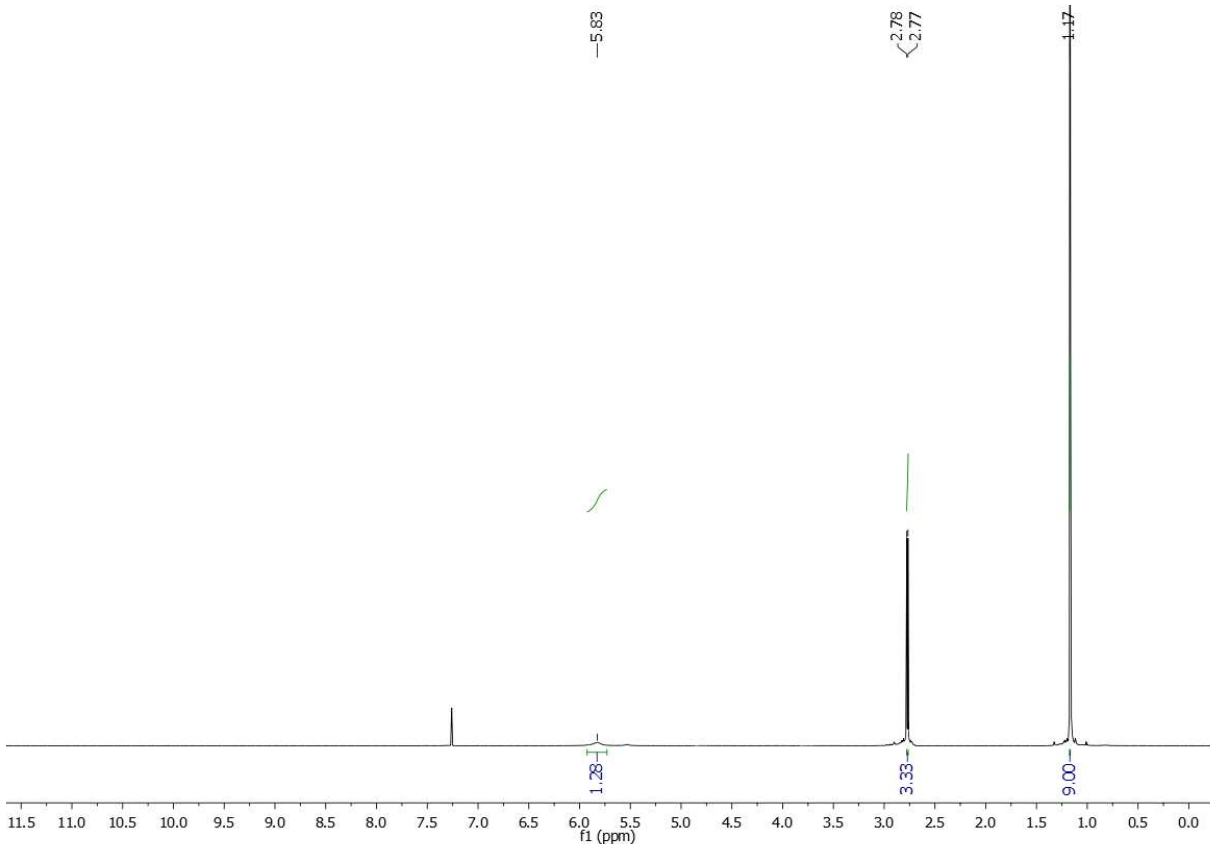
5.83
2.78
2.77
1.17
1.28
3.33
9.00
11.5 11.0 10.5 10.0 9.5 9.0 8.5 8.0 7.5 7.0 6.5 6.0 5.5 5.0 4.5 4.0 3.5 3.0 2.5 2.0 1.5 1.0 0.5 0.0
f1 (ppm)

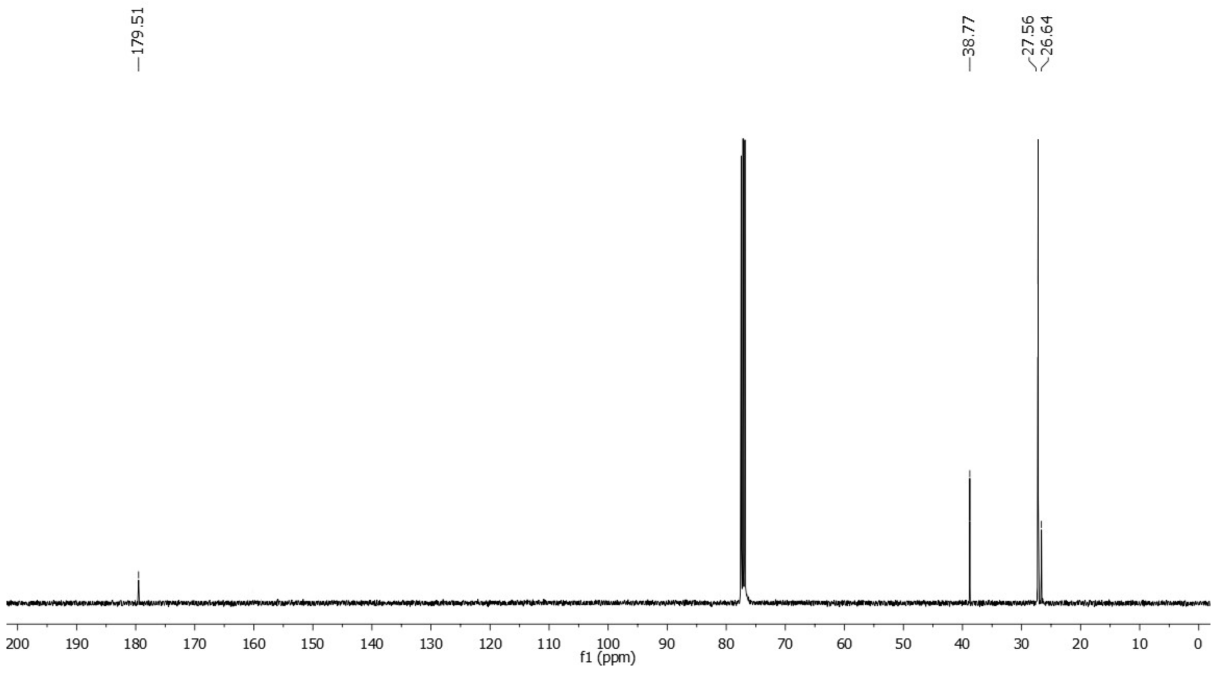
179.51
38.77
27.56
26.64
200 190 180 170 160 150 140 130 120 110 100 90 80 70 60 50 40 30 20 10 0
f1 (ppm)

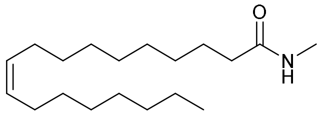
O
N
H

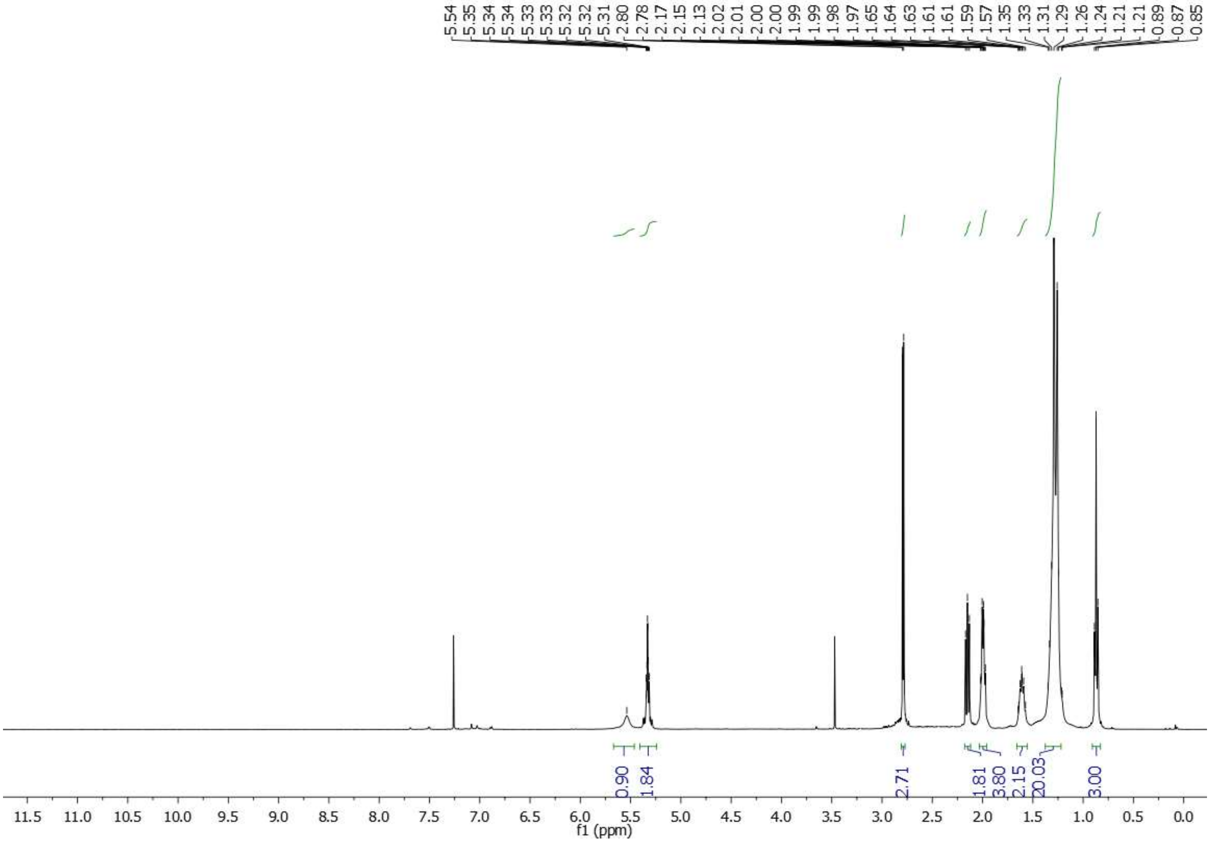
5.54
5.35
5.34
5.34
5.33
5.33
5.33
5.32
5.31
2.80
2.78
2.17
2.15
2.13
2.02
2.01
2.00
2.00
1.99
1.99
1.98
1.97
1.65
1.64
1.63
1.61
1.61
1.59
1.57
1.35
1.33
1.31
1.29
1.26
1.24
1.21
1.21
0.89
0.87
0.85
0.90
1.84
2.71
1.81
3.80
2.15
20.03
3.00
f1 (ppm)

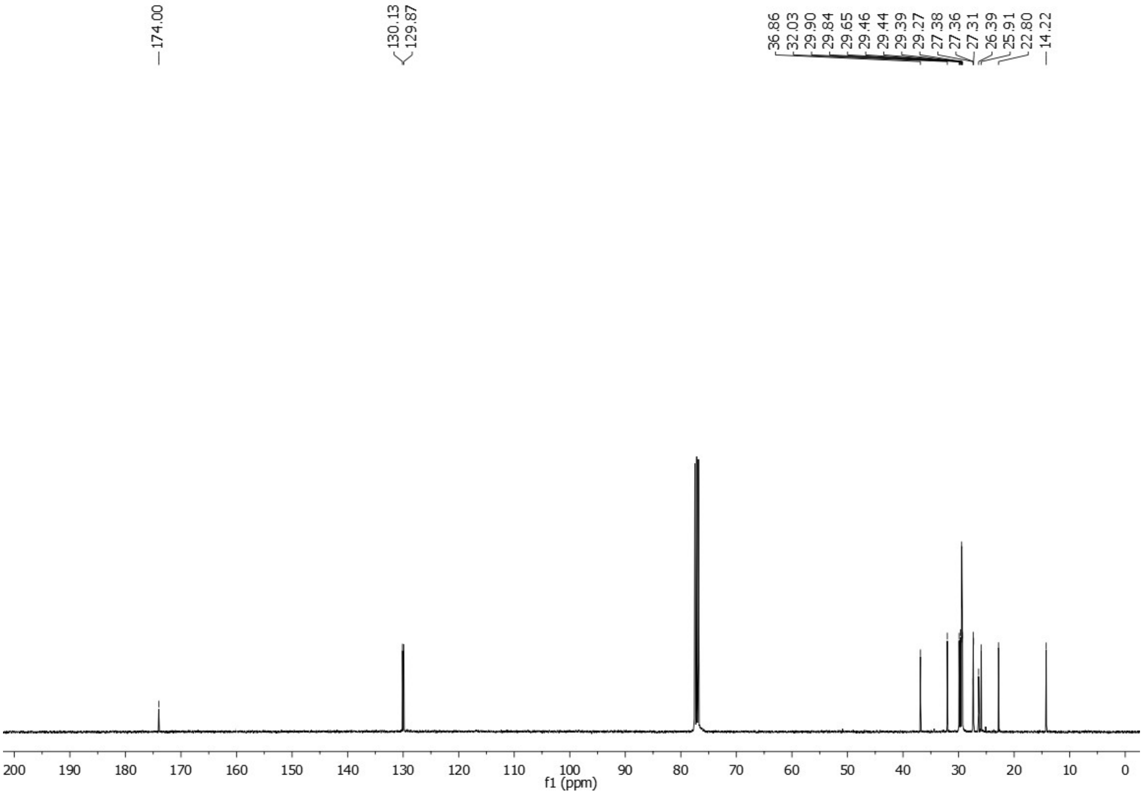
174.00
130.13
129.87
36.86
32.03
29.90
29.84
29.65
29.46
29.44
29.39
29.27
27.38
27.36
27.31
26.39
25.91
22.80
14.22
f1 (ppm)

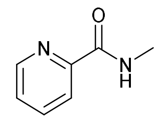

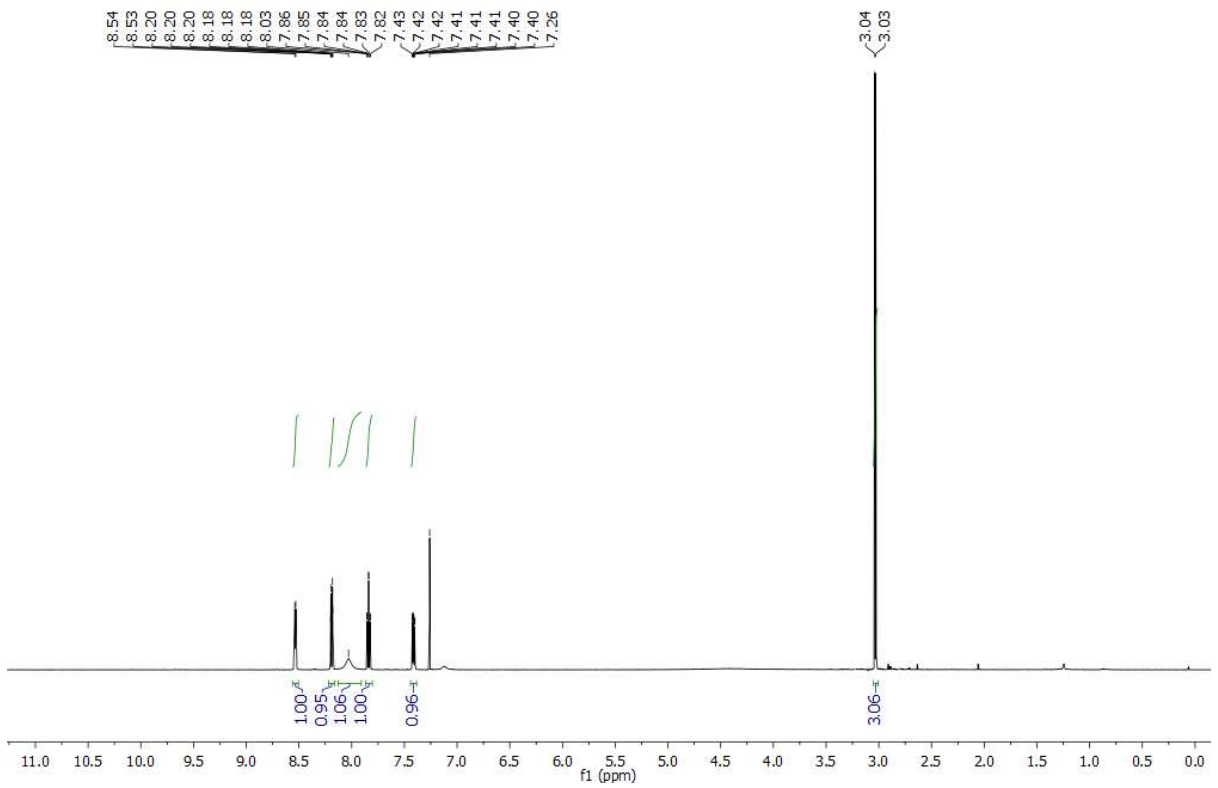
8.54
8.53
8.20
8.20
8.20
8.18
8.18
8.18
8.03
7.86
7.85
7.84
7.84
7.83
7.82
7.43
7.42
7.42
7.41
7.41
7.41
7.40
7.40
7.26
3.04
3.03
1.00
0.95
1.06
1.00
0.96
3.06
f1 (ppm)

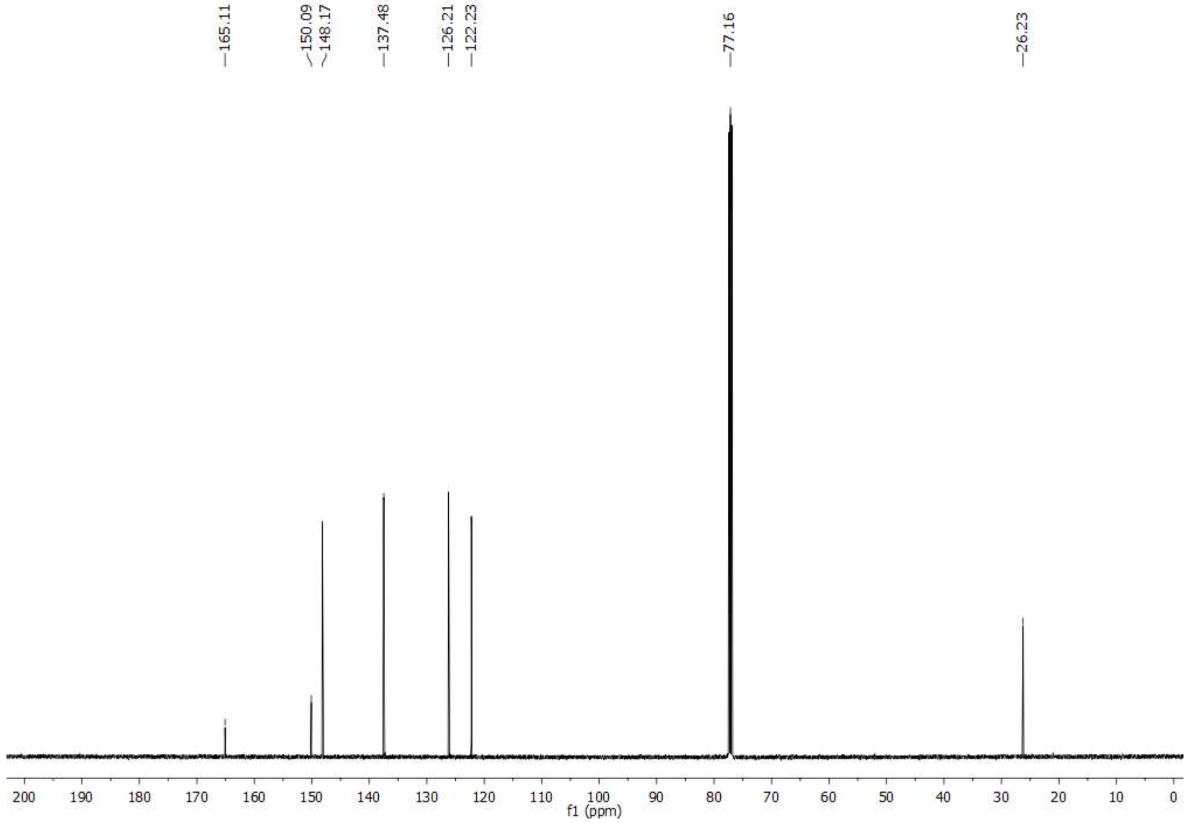
165.11
150.09
148.17
137.48
126.21
122.23
77.16
26.23
f1 (ppm)

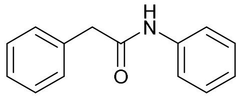
H
N
O

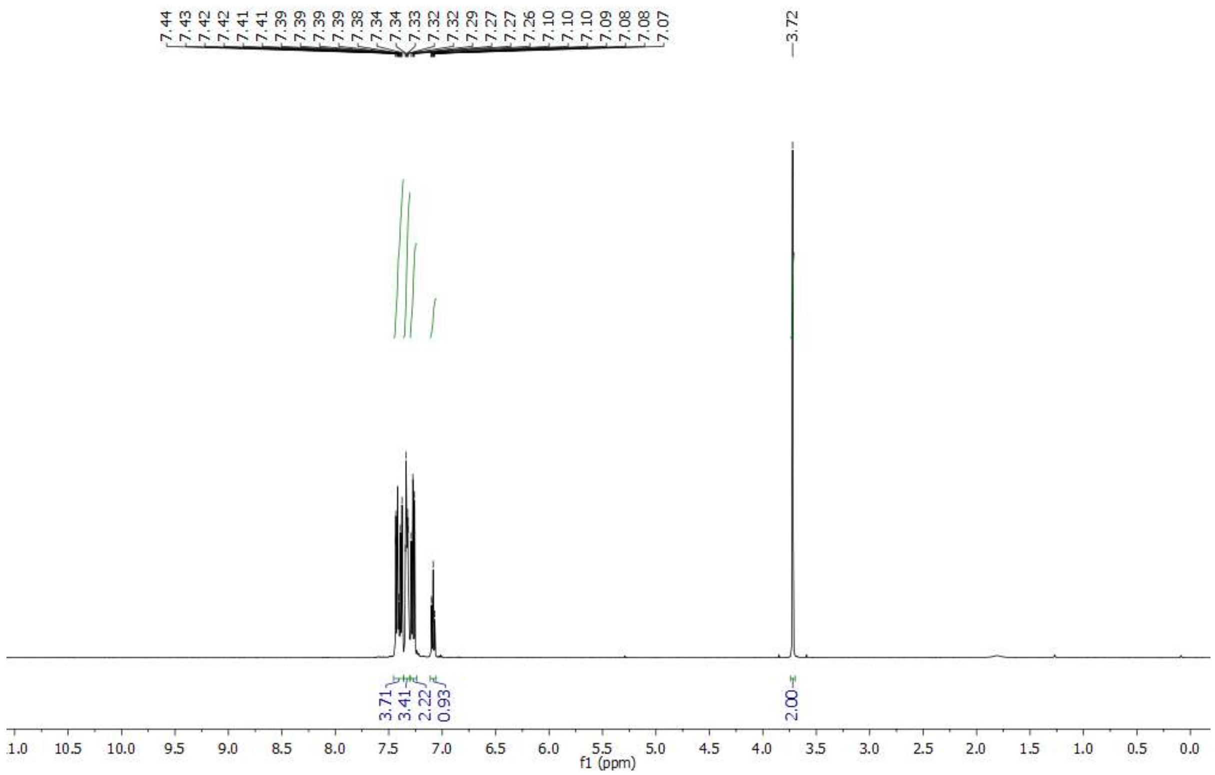
7.44
7.43
7.42
7.42
7.41
7.41
7.39
7.39
7.39
7.39
7.38
7.34
7.34
7.33
7.32
7.32
7.29
7.27
7.27
7.26
7.10
7.10
7.10
7.09
7.08
7.08
7.07
3.72
3.71
3.41
2.22
0.93
2.00
f1 (ppm)

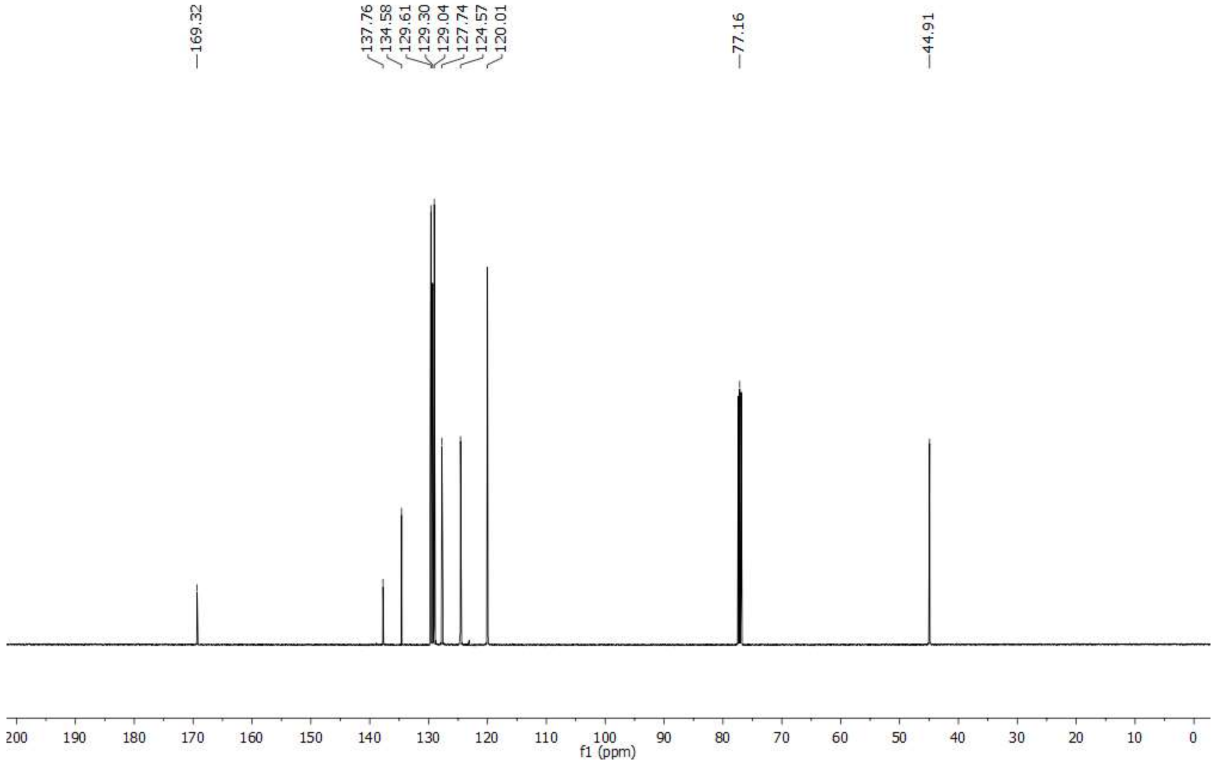
169.32
137.76
134.58
129.61
129.30
129.04
127.74
124.57
120.01
77.16
44.91
f1 (ppm)

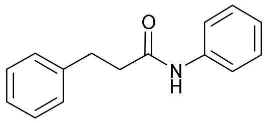

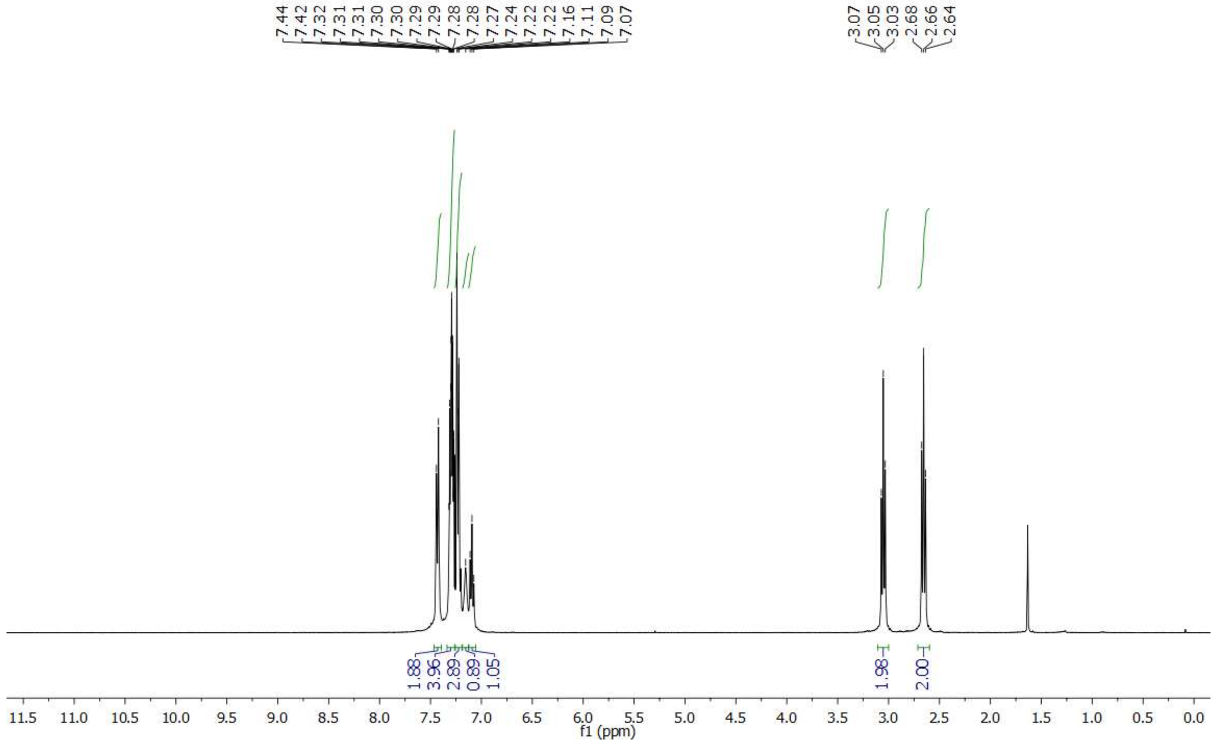
7.44
7.42
7.32
7.31
7.31
7.30
7.30
7.29
7.29
7.28
7.28
7.27
7.24
7.22
7.22
7.16
7.11
7.09
7.07
3.07
3.05
3.03
2.68
2.66
2.64
1.88
3.96
2.89
0.89
1.05
1.98
2.00
f1 (ppm)

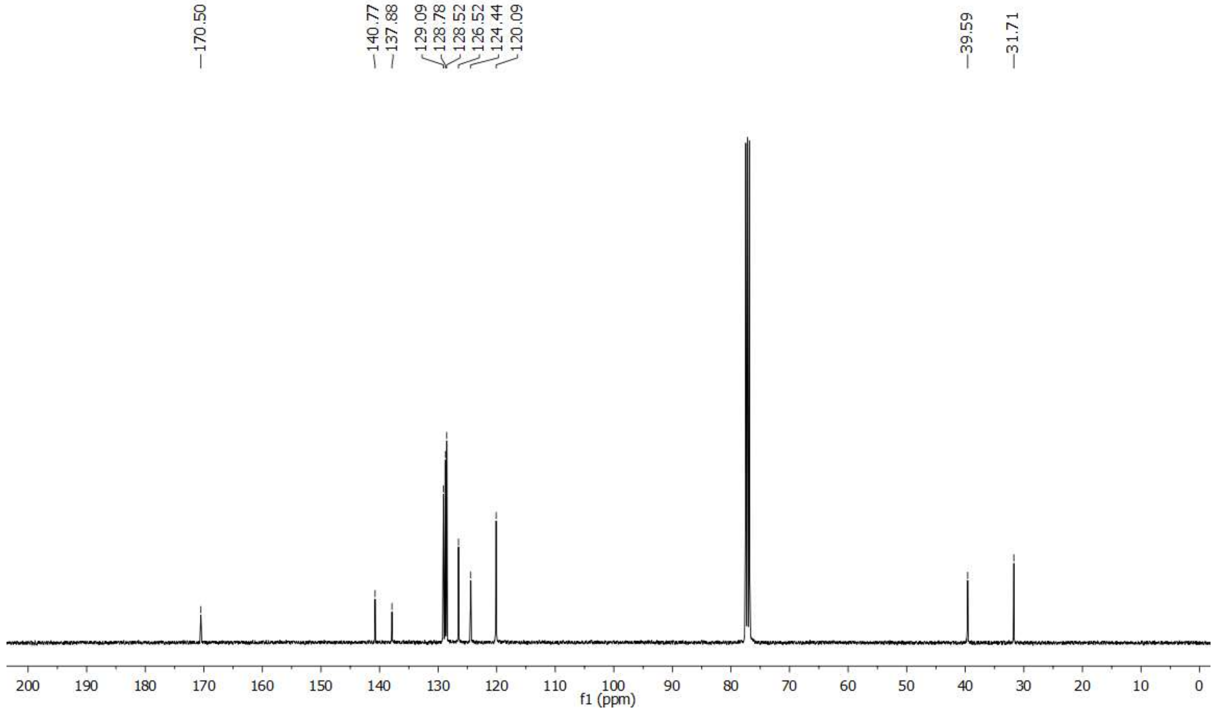
170.50
140.77
137.88
129.09
128.78
128.52
126.52
124.44
120.09
39.59
31.71
f1 (ppm)

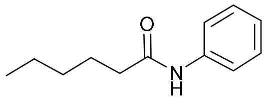

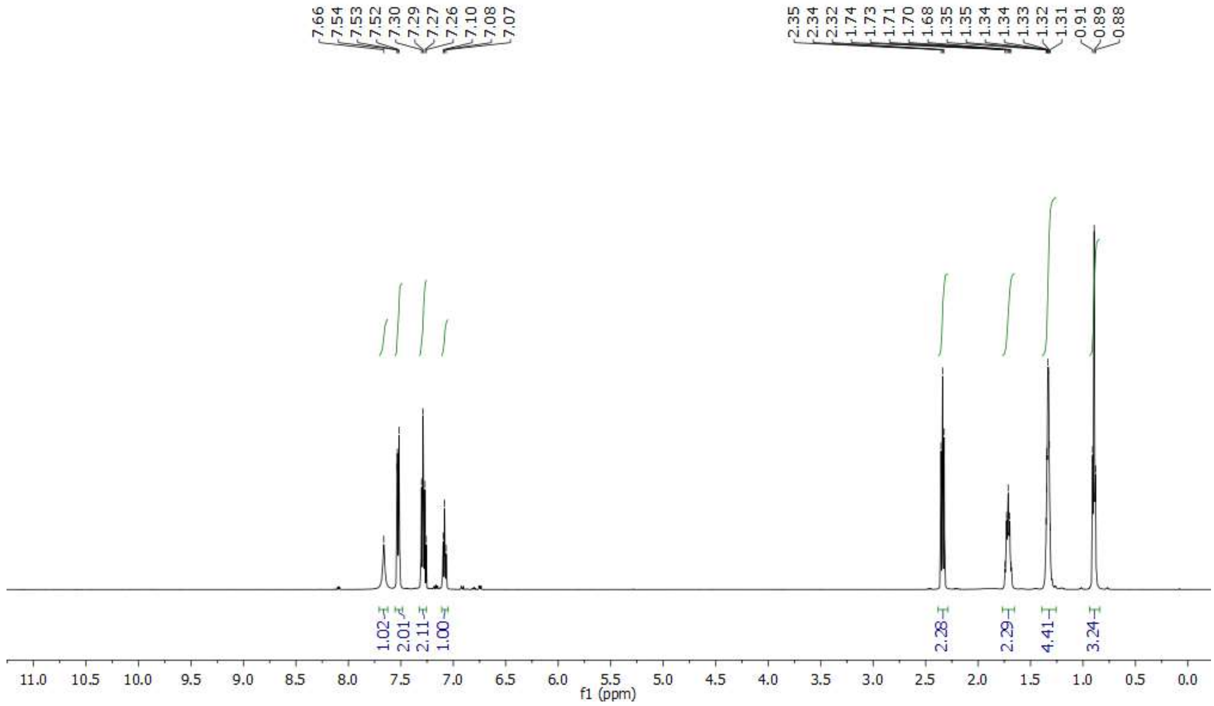
7.66
7.54
7.53
7.52
7.30
7.29
7.27
7.26
7.10
7.08
7.07
2.35
2.34
2.32
1.74
1.73
1.71
1.70
1.68
1.35
1.35
1.34
1.34
1.33
1.32
1.31
0.91
0.89
0.88
1.02
2.01
2.11
1.00
2.28
2.29
4.41
3.24
f1 (ppm)

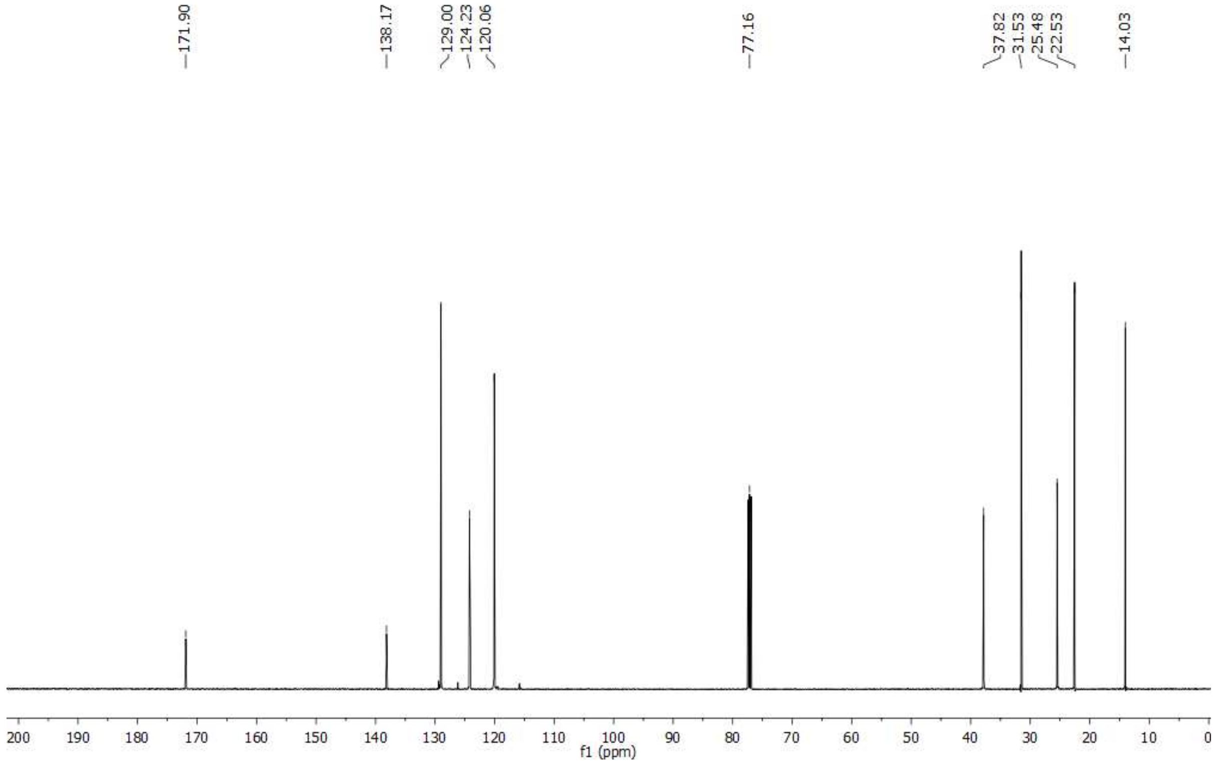
171.90
138.17
129.00
124.23
120.06
77.16
37.82
31.53
25.48
22.53
14.03
f1 (ppm)

11. References

1 P. Xie, C. Xia and H. Huang, *Org. Lett.*, 2013, **15**, 3370–3373.

2 D. Kaufmann, M. Bialer, J. A. Shimshoni, M. Devor and B. Yagen, *J. Med. Chem.*, 2009, **52**, 7236–7248.

3 J. D. Taylor, G. B. Trimitsis, T. Hudlicky and J. F. Wolfe, *J. Org. Chem.*, 1973, **38**, 1236–1238.

4 G. S. Skinner and C. B. Miller, *J. Am. Chem. Soc.*, 1953, **75**, 977–979.

5 C. L. Allen, C. Burel and J. M. J. Williams, *Tetrahedron Lett.*, 2010, **51**, 2724–2726.

6 H. Chen, W. Dai, Y. Chen, Q. Xu, J. Chen, L. Yu, Y. Zhao, M. Ye and Y. Pan, *Green Chem.*, 2014, **16**, 2136–2141.

7 H. Veisi, B. Maleki, M. Hamelian and S. S. Ashrafi, *RSC Adv.*, 2015, **5**, 6365–6371.

8 F. Tiemann, *Berichte der Dtsch. Chem. Gesellschaft*, 1891, **24**, 2877–2879.

9 G. Hahn and H. J. Schulz, *Berichte der Dtsch. Chem. Gesellschaft (A B Ser.)*, 1939, **72**, 1302–1308.

10 A. Yoshimura, K. R. Middleton, M. W. Luedtke, C. Zhu and V. V. Zhdankin, *J. Org. Chem.*, 2012, **77**, 11399–11404.

11 M. Kitamura, S. Miyagawa and T. Okauchi, *Tetrahedron Lett.*, 2011, **52**, 3158–3161.

12 B. Gutmann, T. N. Glasnov, T. Razzaq, W. Goessler, D. M. Roberge and C. O. Kappe, *Beilstein J. Org. Chem.*, 2011, **7**, 503–517.

13 H. Morimoto, R. Fujiwara, Y. Shimizu, K. Morisaki and T. Ohshima, *Org. Lett.*, 2014, **16**, 2018–2021.

14 K. Y. Koltunov, S. Walspurger and J. Sommer, *Eur. J. Org. Chem.*, 2004, 4039–4047.

15 F. R. Johnson and A. Robertson, *J. Chem. Soc.*, 1930, 21–26.

16 G. Dewynter, A. H. Hajri, L. Toupet and J.-L. Montero, *Synth. Commun.*, 2000, **30**, 2541–2548.

17 A. Khalafi-Nezhad, A. Parhami, M. N. Soltani Rad and A. Zarea, *Tetrahedron Lett.*, 2005, **46**, 6879–6882.

18 M. A. Ali and T. Punniyamurthy, *Adv. Synth. Catal.*, 2010, **352**, 288–292.

19 Y. Kita, Y. Nishii, A. Onoue and K. Mashima, *Adv. Synth. Catal.*, 2013, **355**, 3391–3395.

20 G. E. Philbrook, *J. Org. Chem.*, 1954, **19**, 623–625.

21 M. Z. Xu, W. S. Lee, M. J. Kim, D. S. Park, H. Yu, G. R. Tian, T. S. Jeong and H. Y. Park, *Bioorganic Med. Chem. Lett.*, 2004, **14**, 4277–4280.

22 H. Wu, C. J. Kelley, A. Pino-Figueroa, H. D. Vu and T. J. Maher, *Bioorg. Med. Chem.*, 2013, **21**, 5188–5197.

23 H. Li, B. Cheng, N. Boonnak and A. Padwa, *Tetrahedron*, 2011, **67**, 9829–9836.

24 G. E. Veitch, K. L. Bridgwood and S. V Ley, *Org. Lett.*, 2008, **10**, 3623–3625.

25 Y. Huang, T. Chen, Q. Li, Y. Zhou and S.-F. Yin, *Org. Biomol. Chem.*, 2015, **13**, 7289–7293.

26 M. A. Ali and T. Punniyamurthy, *Adv. Synth. Catal.*, 2010, **352**, 288–292.

27 G. B. Payne, *J. Org. Chem.*, 1961, **26**, 668–670.

28 X.-F. Wu, M. Sharif, J.-B. Feng, H. Neumann, A. Pews-Davtyan, P. Langer and M. Beller, *Green Chem.*, 2013, **15**, 1956–1961.

29 D. A. Shirley and M. D. Cameron, *J. Am. Chem. Soc.*, 1950, **72**, 2788–2789.

30 J. Jammot, R. Pascal and A. Commeyras, *Tetrahedron Lett.*, 1989, **30**, 563–564.

31 T. Kanda, A. Naraoka and H. Naka, *J. Am. Chem. Soc.*, 2019, **141**, 825–830.

32 D. G. Pintori and M. F. Greaney, *Org. Lett.*, 2011, **13**, 5713–5715.

33 L. G. Ulysse, Q. Yang, M. D. McLaws, D. K. Keefe, P. R. Guzzo and B. P. Haney, *Org. Process Res. Dev.*, 2010, **14**, 225–228.

34 Q. Yang, L. G. Ulysse, M. D. McLaws, D. K. Keefe, P. R. Guzzo and B. P. Haney, *Org. Synth.*, 2012, **89**, 44–54.

35 D. W. Slocum and C. A. Jennings, *J. Org. Chem.*, 1976, **41**, 3653–3664.

36 R. R. Nani and S. E. Reisman, *J. Am. Chem. Soc.*, 2013, **135**, 7304–7311.

37 S. Hirner and P. Somfai, *J. Org. Chem.*, 2009, **74**, 7798–7803.

38 G. Negri and C. Kascheres, *J. Heterocycl. Chem.*, 2001, **38**, 109–123.

39 S. Hanada, T. Ishida, Y. Motoyama and H. Nagashima, *J. Org. Chem.*, 2007, **72**, 7551–7559.

40 B. Zambroń, M. Masnyk, B. Furman, P. Kalicki and M. Chmielewski, *Tetrahedron*, 2010, **66**, 8974–8981.

41 M. So, T. Kotake, K. Matsuura, M. Inui and A. Kamimura, *J. Org. Chem.*, 2012, **77**, 4017–4028.

42 Q. Xia, X. Liu, Y. Zhang, C. Chen and W. Chen, *Org. Lett.*, 2013, **15**, 3326–3329.

43 Y. Kita, T. Higuchi and K. Mashima, *Chem. Commun.*, 2014, **50**, 11211–11213.

44 E. Alonso, D. J. Ramón and M. Yus, *Tetrahedron*, 1997, **53**, 14355–14368.

45 G. V. Zyryanov and D. M. Rudkevich, *Org. Lett.*, 2003, **5**, 1253–1256.

46 Eur. Pat., 3095444 (A1), 2016.

47 E. T. Roe, J. T. Scanlan and D. Swern, *J. Am. Chem. Soc.*, 1949, **71**, 2215–2218.

48 S. Cai, X. Yu and Z. Chen, *Spectrochim. Acta Part A Mol. Biomol. Spectrosc.*, 2006, **65**, 616–622.

49 T. B. Mete, A. Singh and R. G. Bhat, *Tetrahedron Lett.*, 2017, **58**, 4709–4712.

50 Y. Yan, Z. Zhang, Y. Wan, G. Zhang, N. Ma and Q. Liu, *J. Org. Chem.*, 2017, **82**, 7957–7963.

51 H. Yao, Y. Tang and K. Yamamoto, *Tetrahedron Lett.*, 2012, **53**, 5094–5098.

52 J. D. Williams, W. J. Kerr, S. G. Leach and D. M. Lindsay, *Angew. Chem. Int. Ed.*, 2018, **57**, 12126–12130.

53 S. I. Lee, S. U. Son and Y. K. Chung, *Chem. Commun.*, 2002, 1310–1311.

54 S. Jammi, S. Sakthivel, L. Rout, T. Mukherjee, S. Mandal, R. Mitra, P. Saha and T. Punniyamurthy, *J. Org. Chem.*, 2009, **74**, 1971–1976.
